# Recent Advances in the Synthesis, Optical Properties, and Applications of Fluorescent Silicon Carbide Quantum Dots

**DOI:** 10.1002/smsc.202500013

**Published:** 2025-06-28

**Authors:** Mahdi Hasanzadeh Azar, Jahanbakhsh Jahanzamin, Zimo Ji, Adrian Kitai, David Beke, Adam Gali

**Affiliations:** ^1^ Department of Mechanical and Mechatronics Engineering University of Waterloo Waterloo N2L 3G1 Ontario Canada; ^2^ Department of Engineering Physics McMaster Univeristy Hamilton L8S 4L8 Ontario Canada; ^3^ Department of Chemical and Biological Engineering University of Ottawa 161 Louis Pasteur Ottawa K1N 6N5 Ontario Canada; ^4^ Department of Mechanical and Aerospace Engineering Carleton University 1125 Colonel By Drive Ottawa K1S 5B6 Ontario Canada; ^5^ Department of Materials Science and Engineering McMaster University Hamilton L8S 4L8 Ontario Canada; ^6^ Kandó Kálmán Faculty of Electrical Engineering Óbuda University Bécsi út 94‐96 Budapest H‐1034 Hungary; ^7^ HUN‐REN Wigner Research Centre for Physics Institute for Solid State Physics and Optics PO. Box 49 Budapest H‐1525 Hungary; ^8^ Department of Atomic Physics Institute of Physics Budapest University of Technology and Economics Műegyetem rakpart 3 Budapest H‐1111 Hungary; ^9^ MTA‐WFK Lendület “Momentum” Semiconductor Nanostructures Research Group PO. Box 49 Budapest H‐1525 Hungary

**Keywords:** fluorescent quantum dots, optical properties, optoelectronic and biomedical and energy applications, silicon carbide, surface modifications

## Abstract

Earth‐abundant, fluorescent silicon carbide (SiC) quantum dots (QDs) have recently attracted remarkable attention on account of their long‐term chemical and optical stability and impressive biocompatibility. However, there has been a long‐standing debate among researchers concerning whether radiative recombination in SiC QDs is governed by quantum confinement effects or by surface‐related states. Herein, the underlying mechanism responsible for the photoluminescence observed in SiC QDs is elucidated. Significant progress made through the development of advanced strategies for synthesizing ultrasmall SiC QDs and modifying their surfaces with functional groups, conjugated molecules, and protective shells is discussed. Subsequently, the potential for engineered SiC QDs to be applied to a range of sectors, including energy (photocatalytic‐based CO_2_ reduction systems), electronics/optoelectronics (electroluminescent white light‐emitting diodes, nonlinear optics, and quantum sensing), and biomedicine (cell imaging and biosensors), is reviewed. Finally, this review is summarized with some forward‐looking challenges and prospects.

## Graphical abstract

Fluorescent silicon carbide quantum dots with excellent biocompatibility and chemical stability are gaining increasing attention. This review resolves the long‐standing debate on their emission origin, demonstrating the dominant role of surface states in radiative recombination. It further highlights recent progress in synthesis strategies, surface engineering for enhanced photoluminescence quantum yield, and applications in energy, biosensing/bioimaging, and optoelectronics.

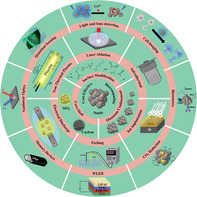

## 1 Introduction

In the last decades, one of the most commercially important wide bandgap semiconductors is silicon carbide (SiC).^[^ ^1^, ^2^, ^3^ ^]^ Due to the strong covalent bond between the Si and C atoms, SiC is considered one of the stiffest materials with high thermal and chemical stability.^[^ ^4^ ^]^ In addition, it benefits from high electric field breakdown (2 × 10^6^ V cm^−1^) and high electron velocity saturation (2 × 10^7^ cm s^−1^).^[^ ^5^ ^]^ Due to these outstanding features, SiC has been applied to a broad range of applications, including turbine blades, diesel engine parts, nuclear reactor walls, abrasives, Schottky diodes, and FET/MOSFET transistors.^[^ ^6^ ^]^ The SiC electronic device market is growing at a cumulative annual growth rate of over 24% and is projected to reach a market value of almost US $ 6 billion by 2027.^[^ ^7^, ^8^ ^]^

Despite its many merits, SiC polytypes all suffer from challenging optoelectronic properties originating from their indirect bandgaps.^[^ ^9^ ^]^ In this case, to generate an electron–hole pair by photon absorption or to emit a photon from electron–hole pair recombination, the contribution of one or more phonons is required to achieve conservation of wavenumber *k* or momentum *p* during the indirect gap transition, significantly decreasing the probability of radiative recombination.^[^ ^10^ ^]^

To enhance radiative recombination in SiC, various approaches have been adopted. One of the effective methods is introducing optically active defects in the SiC matrix, including co‐dopants, surface defects, and stacking faults.^[^ ^11^, ^12^, ^13^, ^14^, ^15^, ^16^, ^17^, ^18^, ^19^ ^]^ Another approach to enable radiative recombination is etching the surface of SiC to make a fluorescent porous structure with surface defects. This porous SiC nanostructure, normally prepared by electrochemical anodization etching, demonstrates photon emission across various wavelengths in the visible spectrum,^[^ ^20^, ^21^, ^22^ ^]^ which may also be dependent on temperature.^[^ ^9^, ^23^ ^]^ Among all strategies, forming quantum dots (QDs) by reducing the size of SiC in all three dimensions to below exciton Bohr diameter has been proposed as a key mechanism that significantly enhances the probability of radiative recombination.^[^ ^24^, ^25^ ^]^ However, there are two main challenges.

First, in terms of optical properties, understanding and modeling the origin of SiC QD emission is challenging. Although the quantum confinement effect (QCE) was believed to underlie the key optical properties in SiC QDs, the surface states effect was introduced as another factor controlling the optical properties of ultrasmall QDs with high surface‐to‐volume ratios. This provided a great opportunity to manipulate surface chemistry by various functional groups, organic molecules, and shells for tuning the emission wavelength and photoluminescence quantum yield (PLQY) to be used for optoelectronic and biomedical applications.^[^ ^26^, ^27^, ^28^, ^29^ ^]^

Second, although many attempts have been made to synthesize SiC QDs, most of the commonly used processes such as ball milling, chemical vapor deposition (CVD), sol–gel, and carbothermal have been incapable of preparing small sizes of high‐quality fluorescent SiC QDs.^[^ ^30^, ^31^ ^]^ Recently, a broad spectrum of sophisticated top‐down (or bottom‐up) and ex situ (or in situ) synthesis protocols have been introduced and proven effective in synthesizing ultrasmall fluorescent QDs.^[^ ^32^, ^33^ ^]^ All of these designed methods have their advantages, disadvantages, and challenges in preparing QDs with various morphological, structural, and optical properties.

Despite these challenges, SiC QDs have distinct advantages over other inorganic semiconductor QDs. SiC benefits from the low cost and the abundance of its elements, silicon, and carbon. Although both Si and SiC have indirect bandgap energies, SiC QDs offer superior chemical stability and biocompatibility, particularly compared to halide‐capped silicon QDs. Additionally, achieving blue emission in the 450–470 nm range from Si (*E*_g_ = 1.12 eV) requires extremely small and precisely controlled QDs (diameter (*D*) < 2 nm), which poses significant challenges for large‐scale production.^[^ ^34^ ^]^ In contrast, the wider bandgap of SiC (2.3 eV for cubic phase) enables more efficient generation of blue emission in larger QDs. Lead perovskites,^[^ ^35^, ^36^, ^37^, ^38^ ^]^ CdSe,^[^ ^39^ ^]^ and PbS QDs^[^ ^40^ ^]^ reached high values of PLQY and improved the performance of optoelectronic devices; however, they are toxic and unstable in many environments. Compared to them, SiC QDs are biocompatible, photostable, chemically inert, and long‐term stable in water for several months, enabling them to be used in biomedical applications.^[^ ^41^ ^]^ As an electronic/optoelectronic and energy material, SiC QDs and similarly nanostructured SiC materials have demonstrated some preliminary but intriguing results for potential applications in light‐emitting diodes (LEDs), memory cells, UV detectors, qubits, bio‐implanted sensors, and photocatalytic CO_2_ reduction strategies.^[^ ^42^, ^43^, ^44^ ^]^

Although a few review papers have been published on specific aspects of SiC nanomaterials, no comprehensive review paper on fluorescent SiC QDs has been published. In this article, recent advances in the synthesis, optical properties, and applications of fluorescent SiC nanocrystals are extensively reviewed in detail.

## 2 Structural Properties of SiC

**Figure 1:**
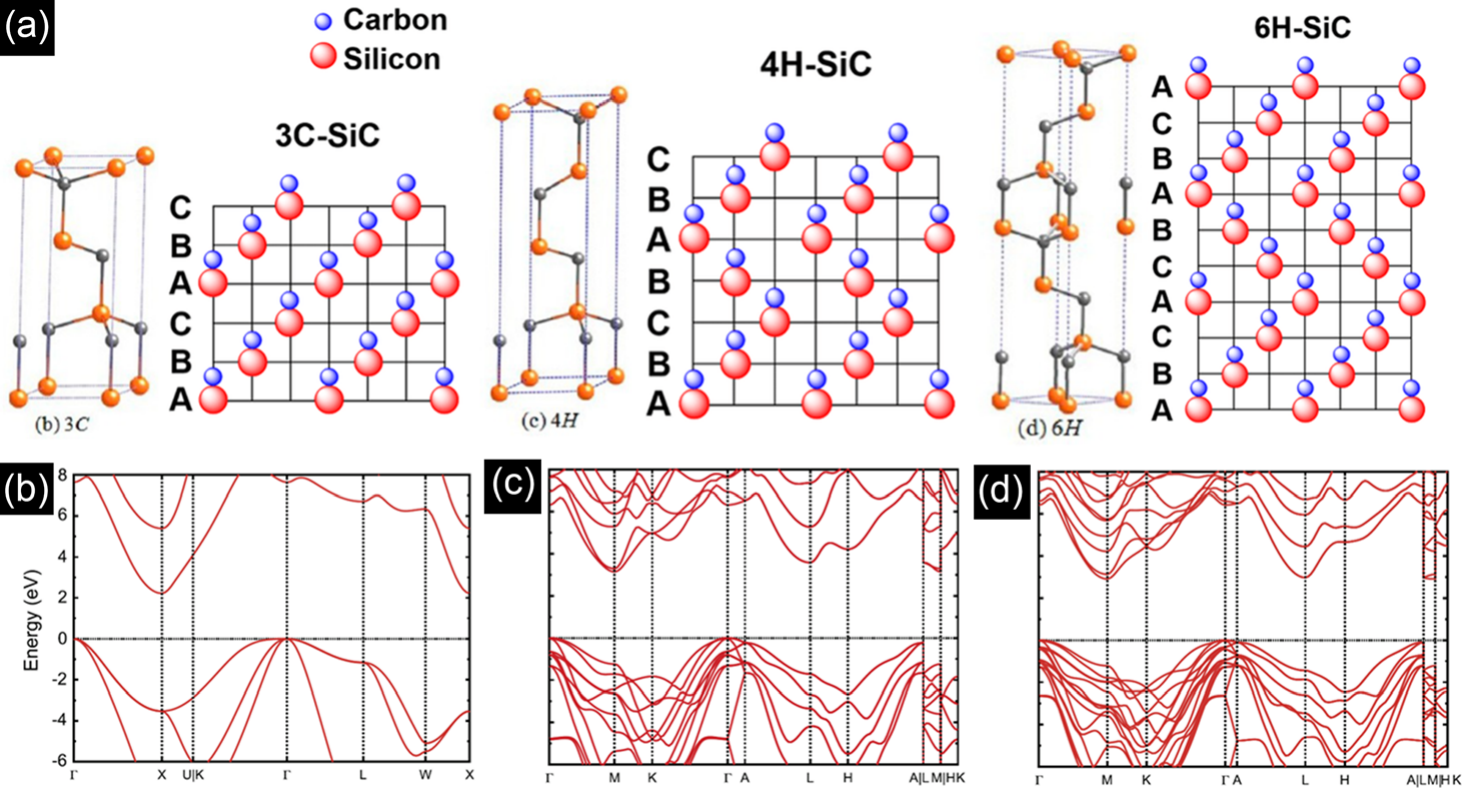
a) The primitive hexagonal unit cells and stacking sequence of 3C‐, 4H‐, and 6H‐SiC. Reproduced with permission.^[^ ^252^ ^]^ Copyright 2013, American Chemical Society. Band structures of b) 3C, c) 4H, and d) 6H SiC. Reproduced with permission.^[^ ^253^ ^]^ Copyright 2020, AIP Publishing.

Strong covalent Si—C bonds in s*p* ^3^ hybridized orbitals endow SiC with a high sublimation temperature of ≈2700 °C and high stiffness.^[^ ^45^ ^]^ SiC has over 250 layer stacking polytypes. Among these, the most commonly observed polytypes formed in bulk crystal growth of SiC are 3C, 6H, and 4H, defining first the number of layers in the unit cell, followed by a letter recording the symmetry of the lattice (C: cubic, H: hexagonal).^[^ ^46^ ^]^ **Figure** **1**a and **Table** **1** show the primitive unit cells, the arrangement of atomic layers, and various properties of SiC polytypes. Shown are the 3C‐SiC (β‐SiC), the ABCACB 6H‐SiC, and the ABCB 4H‐SiC (α‐SiC) arrangements. Among all, 3C‐SiC has the largest fracture toughness, hardness, cohesive energy, and low‐temperature stability.

**Table 1:** Physical, optical, and electrical properties of different SiC polytypes.

Property	3C‐SiC	4H‐SiC	6H‐SiC
Space group	F‐43 m (216)	P6_3_mc (186)	P6_3_mc (186)
Lattice constants [A^°^]	*a* = 4.3579	*a* = 3.0817 *c* = 10.0791	*a* = 3.0817 *c* = 15.1173
Stacking sequence	ABC	ABCB	ABCACB
Bandgap [eV]a)	2.36	3.23	3.02
Intrinsic carrier concentration [cm^−3^]	1.5 × 10^−1^	5 × 10^−9^	1.6 × 10^−6^
Electron mobility [cm^2^ Vs^−1^]	900	800	370
Hole mobility [cm^2^ Vs^‐^]	40	120	80
Electron affinity [eV]	3.8	3.1	3.3
Saturated electron velocity [10^7^ cm s^−1^]	2	2	2
Breakdown field *E*_B_ [MV cm^−1^]	1.5	2.3	2.2

a) Room temperature values.

## 3 Optical Properties of SiC

Figure 1b–d shows the electronic band dispersion of SiC polytypes, revealing indirect bandgaps ranging from 2.3 eV for 3C‐SiC to 3.23 eV for 4H‐SiC. Due to the momentum difference between valence band maximum (VBM) and conduction band minimum (CBM), the probability of radiative recombination is low since phonon participation is necessary.^[^ ^9^ ^]^ To overcome this challenge, several strategies can be adopted, including doping, etching, defect introduction, and size reduction.

### 3.1 Donor–Acceptor Pair Recombination

**Figure 2:**
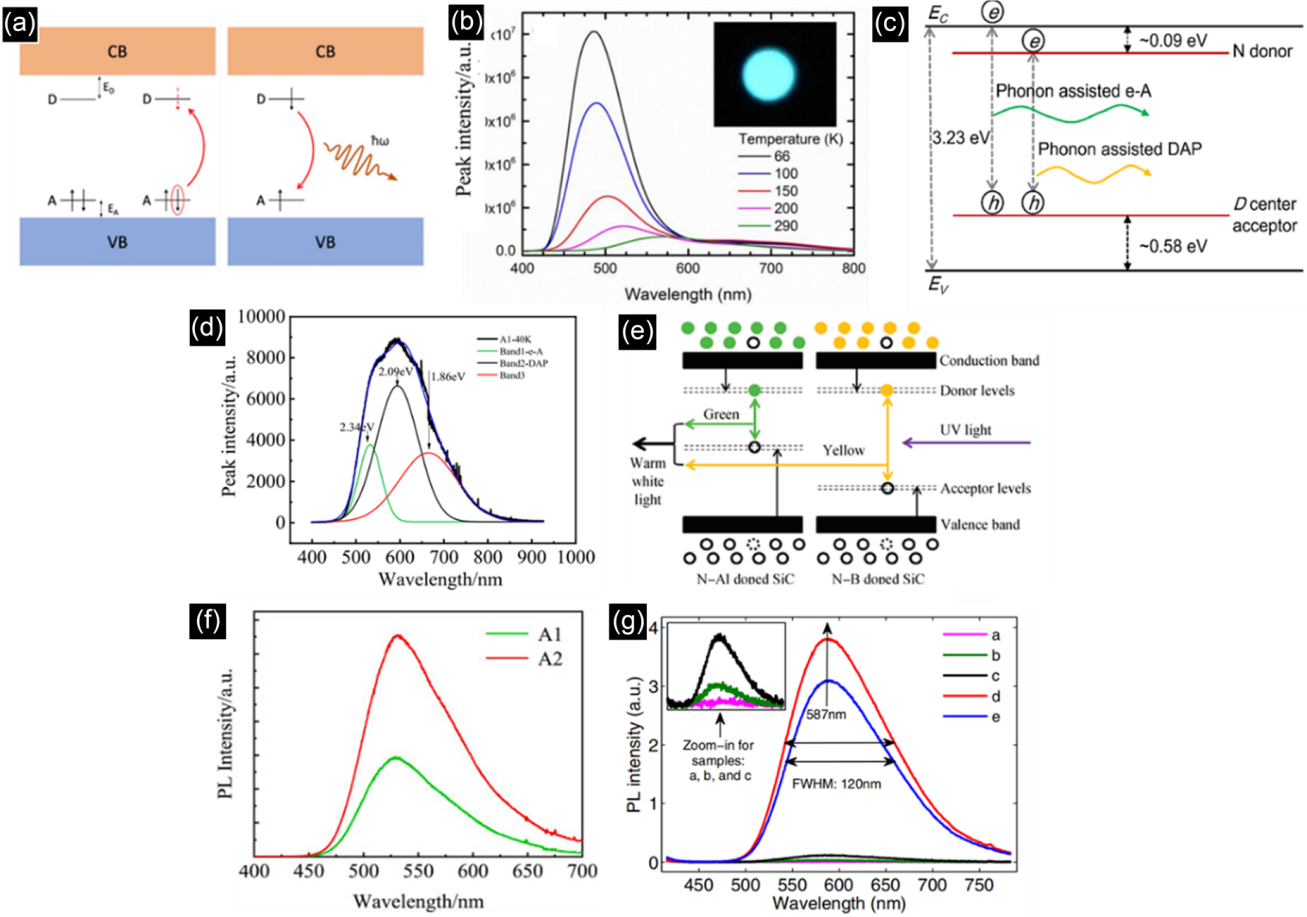
a) Mechanism of donor–acceptor recombination. Reproduced with permission.^[^ ^47^ ^]^ Copyright 2024, Springer Nature. b) PL spectra with N‐Al doped 6H‐SiC at a series of temperatures. Reproduced with permission.^[^ ^49^ ^]^ Copyright 2024, DTU Orbit. c) Illustration of DAP and e‐A recombination within the band structure. Reproduced with permission.^[^ ^14^ ^]^ Copyright 2019, IOP Publishing. d) Fitting PL spectra of co‐doped 4H‐SiC at different temperatures. Reproduced with permission.^[^ ^48^ ^]^ Copyright 2022, Elsevier. e) Mechanism of white light emission by combining two DAPs in 4H‐SiC. Reproduced with permission.^[^ ^51^ ^]^ Copyright 2015, IOP Publishing. f) PL spectra of B‐N doped 6H‐SiC with two boron concentrations: 1.65 × 10^18^ cm^−3^ in A1 and 4.14 × 10^18^ cm^−3^ in A2. Reproduced with permission.^[^ ^48^ ^]^ Copyright 2022, Elsevier. g) PL spectra of B‐N doped 6H‐SiC with series of B and N concentrations (a: *N*_A_ = 8.0 × 10^18^ cm^−3^ and *N*_D_ = 0.04 × 10^18^ cm^−3^, b: *N*_A_ = 6.9 × 10^18^ cm^−3^ and *N*_D_ = 3.2 × 10^18^ cm^−3^, c: *N*_A_ = 6.9 × 10^18^ cm^−3^ and *N*_D_ = 6.0 × 10^18^ cm^−3^, d: *N*_A_ = 4.4 × 10^18^ cm^−3^ and *N*_D_ = 9.0 × 10^18^ cm^−3^, and e: *N*_A_ = 5.2 × 10^18^ cm^−3^ and *N*_D_ = 9.2 × 10^18^ cm^−3^). Reproduced with permission.^[^ ^52^ ^]^ Copyright 2019, IOP Publishing.

Donor and acceptor dopants in SiC can mitigate the adverse effect of the indirect bandgap by inducing donor–acceptor pair (DAP) recombination. Electrons may be excited from the acceptor level to the donor level. In the emission process, the energy difference between these two levels may determine the emission wavelength as indicated in **Figure** **2**a.^[^ ^47^ ^]^ Besides, there are other recombination processes to control the optical properties. As seen in Figure 2b,c, free‐to‐acceptor (e‐A) recombination has been found at 40, 80, and 300 K, respectively, and an additional recombination band (marked as band 3 in Figure 2d) can be observed at low temperatures, which is neither e‐A nor DAP recombination.^[^ ^14^ ^]^ This indicates the importance of temperature on DAP recombination.^[^ ^48^ ^]^

The optical properties of DAP SiC depend on the energy gap of the SiC polytype as well as donors and acceptors. As illustrated in Figure 2e, nitrogen (N)‐aluminum (Al) and N‐boron (B) doped 4H‐SiC, providing green and yellow DAP fluorescence centers, respectively, can be combined to produce white light.^[^ ^49^, ^50^, ^51^ ^]^ Other elements, such as phosphorus (P) and arsenic (As) (donors), and gallium (Ga) and indium (In) (acceptors), can be doped into SiC. Highly efficient DAP recombination has not been reported.

The concentration of doping is one of the key factors to discuss and has been reported to tune the photoluminescence (PL) intensity and peak shift. As exhibited in Figure 2f, a change in boron concentration from 1.65 to 4.14 × 10^18^ cm^−3^ doubles emission intensity.^[^ ^48^ ^]^ Raising the dopant concentration lowers the mean distance between donors and acceptors, enhancing localization and overlap probability of electron and hole wavefunctions. Moreover, the PL results in Figure 2g show that the higher concentration of donors in n‐type 4H‐SiC (samples d and e) achieves much higher intensity than a higher concentration of acceptors in p‐type 4H‐SiC (samples a, b, and c).^[^ ^52^ ^]^ This may be understood from the position of the Fermi level, which rises in more highly n‐type material, thereby favoring the electron population of the donor level that is the required condition at the onset of DAP emission.^[^ ^53^ ^]^

### 3.2 Defects and Impurities

**Figure 3:**
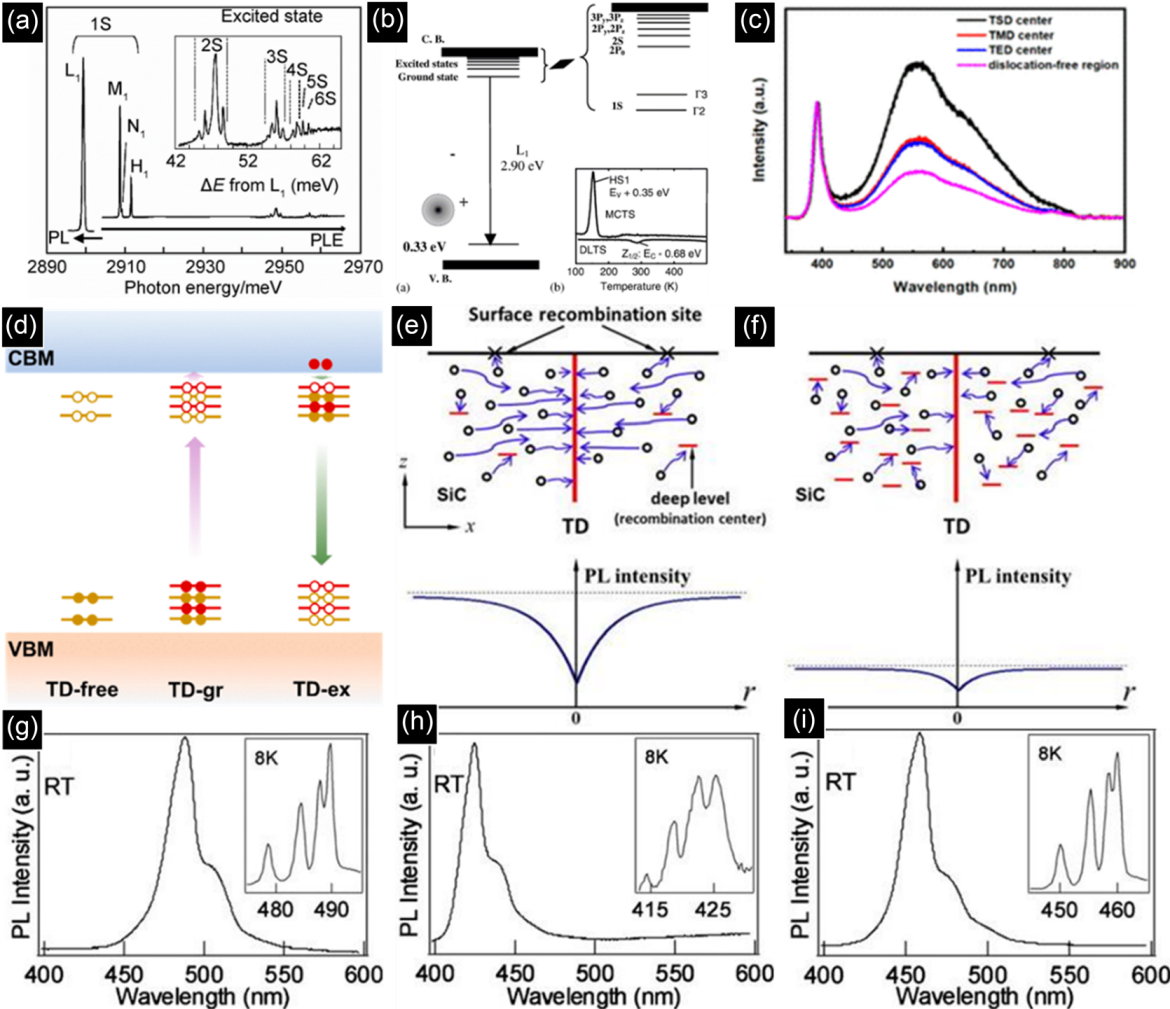
a) PL spectra of D_1_ defects in SiC. b) Recombination mechanism of D_1_ defects. Reproduced with permission.^[^ ^19^ ^]^ Copyright 2003, Elsevier. c) PL spectra for each center of threading dislocations. d) Band structure illustration for threading dislocations. Reproduced with permission.^[^ ^56^ ^]^ Copyright 2022, American Chemical Society. e,f) Recombination related to threading dislocation with deep levels in low and high concentrations. Reproduced with permission.^[^ ^57^ ^]^ Copyright 2011, AIP Publishing. g–i) PL spectra of Frenkel defects in intrinsic, extrinsic, and multiple‐layer SF in 4H‐SiC. Reproduced with permission.^[^ ^58^ ^]^ Copyright 2010, AIP Publishing.

Color centers can also be formed by introducing native defects and impurities. Compared with DAP, fluorescent defects are isolated quantum systems that emit light through electron transitions within localized energy states in the fundamental bandgap.^[^ ^54^ ^]^ There is a detailed review dedicated solely to color centers in SiC.^[^ ^55^ ^]^ In particular, wide bandgap SiC polytypes host numerous color centers. Native defects, such as the divacancy center (VV), silicon‐vacancy (*V*_si_), and antisite‐vacancy pair (*V*_c_ *C*_si_), are known emitters as well as impurity‐related defects, i.e., the nitrogen‐vacancy defect (NV), vanadium, titanium, chromium, molybdenum, and erbium impurities in which spin states are involved in the fluorescence, which can comprise quantum bits when the defects can be isolated. Other intrinsic color centers, such as the isoelectronic D_1_ PL center, which is induced by ion implantation or ion bombardment and has a relatively strong PL signal from cryogenic to room temperatures, are shown in **Figure** **3**a. A recombination mechanism is shown in Figure 3b.^[^ ^19^ ^]^

Higher‐dimensional defects can also contribute to the optical properties of SiC. The threading dislocation is also a common defect, which has been shown to have enhanced nonradiative recombination under measurement of micro‐PL spectra in Figure 3c, and the mechanism of threading dislocation has been explained as in Figure 3d.^[^ ^56^ ^]^ Such defects can decrease the total luminescence intensity and may quench the photoluminescence process. It is shown that there are two competitive processes of recombination during the diffusion of carriers: one is the recombination at threading dislocations, and the other is the recombination at deep levels (Figure 3e,f).^[^ ^57^ ^]^

Stacking faults (SFs) have an observable effect on PL. SFs can provide a fluorescence pathway that yields additional peaks in the PL spectra, as shown in Figure 3g–i.^[^ ^58^ ^]^ SFs can also stabilize the charge states of point defects,^[^ ^59^, ^60^ ^]^ allowing robust room temperature operation, especially targeted toward quantum information processing, where spin‐initialization fidelity is very important.

### 3.3 Porous Structures

**Figure 4:**
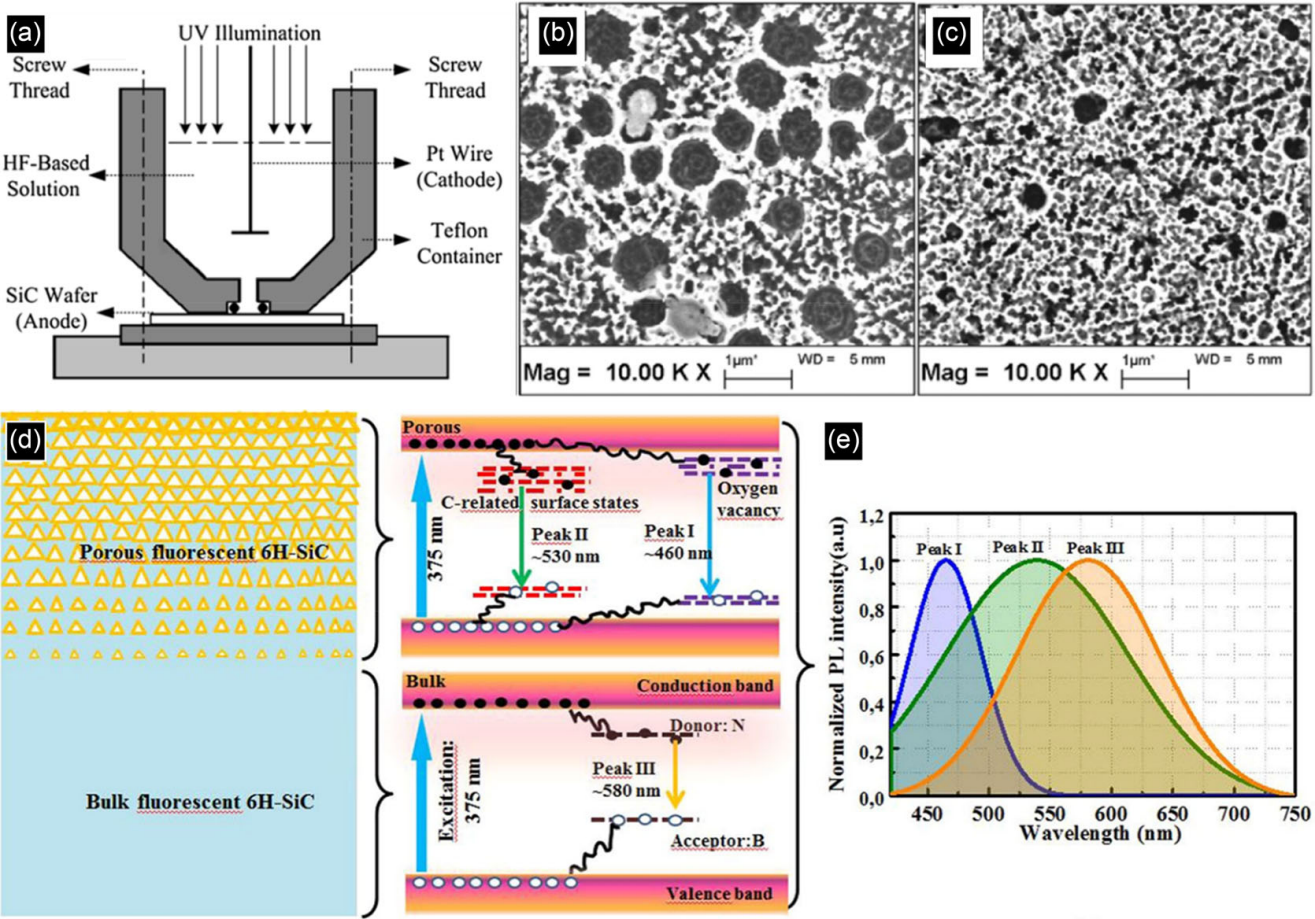
a) Preparation method of porous 6H‐SiC. B,c) Microstructure of porous 6H‐SiC with different pore sizes. Reproduced with permission.^[^ ^62^ ^]^ Copyright 2013, IOP Publishing. d) Structure of 6H‐SiC with bulk substrate and porous layer, as well as the mechanism of each part of the samples within the band structure. e) PL spectra of the prepared 6H‐SiC sample with three peaks corresponding to different mechanisms. Reproduced with permission.^[^ ^66^ ^]^ Copyright 2017, Springer Nature.

As first observed in Si,^[^ ^61^ ^]^ fluorescent porous SiC, produced via electrochemical etching in HF, shows great potential for blue and white light LEDs (**Figure** **4**a).^[^ ^62^ ^]^ It is seen that electrochemical etching parameters such as HF concentration, voltage, and current can affect the porous structure and impact optical properties. For instance, as illustrated in Figure 4b,c, SiC with a range of pore sizes and porosities are produced by different supplied current densities.^[^ ^23^ ^]^

Porous SiC can have quite different optical properties compared to normal bulk SiC, including PL wavelength and intensity. To explain the optical properties of porous SiC, several distinct models have been proposed.^[^ ^63^ ^]^ One theory is quantum confinement, in which visible light emission is seen from the recombination of electrons and holes between discrete energy levels within confined SiC regions bonded by pores. This may have behavior analogous to SiC NCs.^[^ ^64^ ^]^ Another theory supports PL of porous SiC generated from surface defects or oxidized surface layers.^[^ ^20^ ^]^ For example, a fluorescence mechanism may be due to surface chemical states of the atoms and surface moieties composed of SiC and impurities such as oxygen or nitrogen.^[^ ^65^ ^]^ Until now, there has been no general agreement on the mechanism of fluorescence in porous SiC.

A range of optoelectronic and sensor devices may be enabled. A blue LED has been fabricated from indium‐tin‐oxide (ITO)/porous SiC.^[^ ^65^ ^]^ Porous SiC could also be used as a color conversion material for LEDs.^[^ ^20^ ^]^ For instance, porous SiC has been produced on the surface of N‐B co‐doped 6H‐SiC, which can emit light with different wavelengths excited by UV light and can further mix with the emitting light from the bulk substrate SiC to achieve white light (Figure 4d,e).^[^ ^66^ ^]^

### 3.4 Nanostructures

With the development of nanotechnology, nanostructured (two‐dimensional (2D), one‐dimensional (1D), and zero‐dimensional (0D)) materials have received much attention because of their distinguishable properties.^[^ ^67^ ^]^

**Figure 5:**
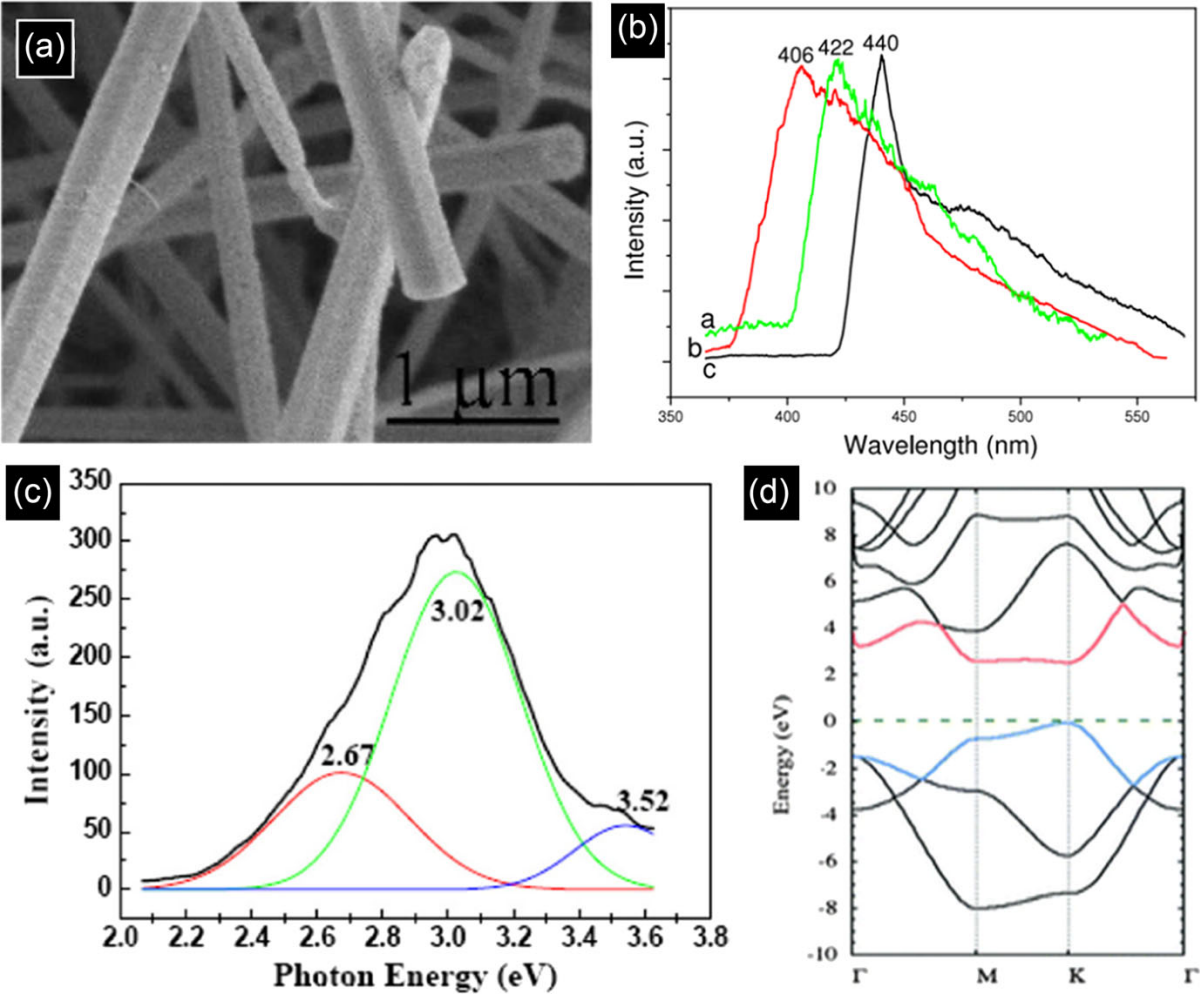
a) 3C‐SiC nanowires with nanocylinder morphology. b) PL spectra of each nanostructure. Reproduced with permission.^[^ ^83^ ^]^ Copyright 2008, IOP Publishing. c) PL spectra of 6H‐SiC nanowires. Reproduced with permission.^[^ ^84^ ^]^ Copyright 2008, IOP Publishing. d) Computational band structure of 2D SiC. Reproduced with permission.^[^ ^47^ ^]^ Copyright 2024, Springer Nature.

Theoretical calculations have predicted various fascinating physical properties,^[^ ^68^, ^69^, ^70^ ^]^ including direct bandgap for 2D SiC as seen in **Figure** **5**d, resulting from quantum confinement.^[^ ^47^ ^]^ SiC with a 3D covalently bonded network, formed by s*p* ^3^ hybridized bonds between Si and C, cannot serve as the parent of a 2D counterpart for mechanical exfoliation, giving limited success for the realization of 2D SiC. Recent studies, however, demonstrated successful liquid exfoliation of SiC in isopropyl alcohol or N‐methyl‐2‐pyrrolidone.^[^ ^71^ ^]^ The bottom‐up growth of 2D SiC has also been demonstrated by various researchers.^[^ ^72^, ^73^ ^]^ Only Chabi et al. reported PL spectra of solvent‐exfoliated SiC nanosheets with a fluorescence maximum of 480 nm (2.58 eV). The synthesis of 2D SiC is in need of confirmation by other researchers, however preliminary results are promising. Furthermore, density functional theory calculations imply that color centers in 2D SiC can be engineered for quantum technologies.^[^ ^74^ ^]^

SiC nanowires have been developed extensively and can be grown by a gas‐phase‐based process. Normally, polymer pyrolysis is applied, i.e., polyureasilazane.^[^ ^75^, ^76^, ^77^ ^]^ The formation of nanowires is typically described using the vapor–liquid–solid (VLS) and vapor–solid (VS) methods. The key distinction between these two processes lies in the use of a catalyst in the VLS method, whereas the VS method does not involve one. Other methods, i.e., carbothermal reduction,^[^ ^78^ ^]^ chemical CVD,^[^ ^79^, ^80^ ^]^ thermal evaporation, and hydrothermal synthesis,^[^ ^81^, ^82^ ^]^ have been used as well. Luminescence properties have been explored from 3C‐SiC SiC nanowires shown in Figure 5a,b ^[^ ^83^ ^]^ and 6H‐SiC nanowires shown in Figure 5c.^[^ ^84^ ^]^ The PL spectra of SiC nanowires (NWs) tend to be broad and features multiple components. The peak maxima of these spectra can range from 290 to 800 nm, influenced by the synthesis method and the final structure of the NWs. Researchers often attribute the optical properties of SiC NWs, along with other nanostructures, to the QCE. However, these NWs typically have diameters above 10 nm, too large to exhibit strong QCE. Nevertheless, it is conceivable that the NWs contain smaller segments or crystallites where QCE could occur. More likely, the surface states of the NWs play a crucial role in determining their optical properties.

**Figure 6:**
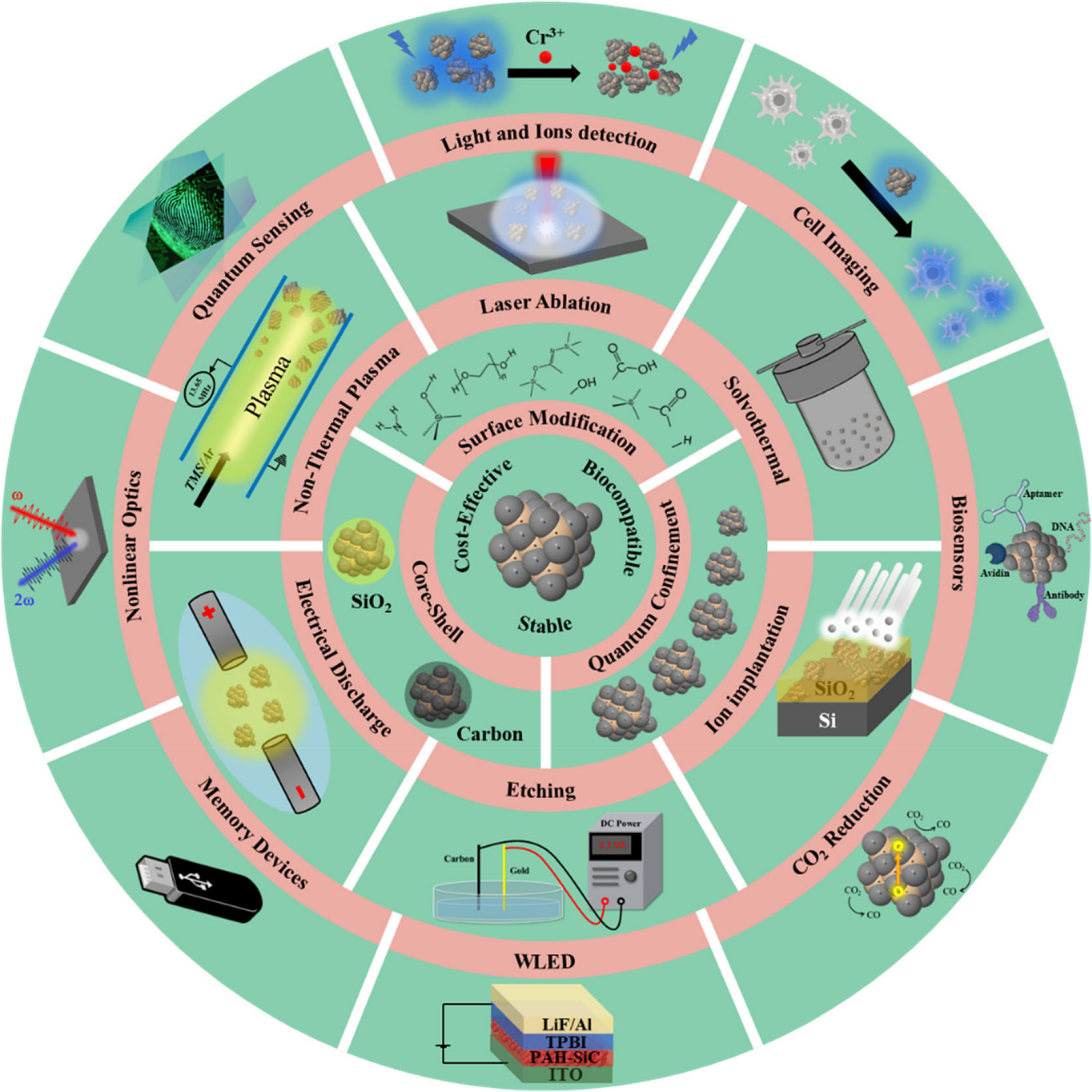
Schematic diagram of a broad spectrum of developments in synthesis, optical properties, and applications of SiC QDs.

Bulk electronic and optical properties of SiC may be dramatically modified and improved by forming 0D SiC structures. This is currently an active field of research and development and is the focus of this review paper. **Figure** **6** illustrates effective approaches used to synthesize SiC QDs, improve their optical properties, and utilize them for various applications. Synthesis methods of SiC QDs are discussed in detail in Section 4, and in Sections 5 and 6, their optical properties and applications are presented.

## 4 Synthesis Methods of SiC QDs

To synthesize SiC QDs, both top‐down and bottom‐up methods are employed. In top‐down methods, the main challenge is providing a powerful driving force for breaking the strong covalent bonds in SiC wafers or powders. Other challenges inherent in the top‐down approach include undesired phase transformations between SiC polytypes as well as the formation of defects and disorder in the crystal structure. In contrast, although the bottom‐up processes benefit from facile fabrication processes, finding liquid‐based silicon‐carbon precursors with a precise stoichiometric ratio between Si and C has remained an unsolved problem.

In the last decade, most of the adopted top‐down and bottom‐up methods, including ball milling,^[^ ^85^ ^]^ sol–gel,^[^ ^86^ ^]^ microwave sintering,^[^ ^87^ ^]^ CVD,^[^ ^88^ ^]^ carbothermal reduction,^[^ ^89^, ^90^ ^]^ high‐energy ball milling,^[^ ^91^ ^]^ and liquid phase sintering^[^ ^92^ ^]^ have proven to be incapable of controlling the composition while achieving the size of SiC NCs to less than the exciton Bohr diameter (less than 5 nm for 3C‐SiC).^[^ ^93^ ^]^ Shortcomings in the ability of these somewhat traditional methods to synthesize NCs with high crystallinity and uniform particle size distribution are clearly evident. In addition, these known methods suffer from high preparation costs, long processing times, environmentally harmful precursors, and low product purity. For instance, carbothermic reduction requires high temperatures and long reaction time, coarsening the SiC particles, while CVD uses hazardous SiH_4_, CH_4_, and SiCl_4_ precursors.^[^ ^94^ ^]^

Therefore, researchers have recently strived to introduce a wide range of innovative and more efficient and effective processes, which are capable of preparing a stable suspension of ultrasmall SiC NCs, as presented in the following sections. **Table** **2** describes the details of these processes.

**Table 2:** The list of processes used for the synthesis of SiC QDs.

Synthesis method	Materials or media	Phase of SiC QDs	Size [nm]	Conditions	Reference
Chemical etching	1) SiC NPs Diameter: 100–500 nm 2) HNO_3_ and HF (v/v = 1:3)	3C	2.5	1) Etching for 1 h 2) Ultrasonication for 25 min	[95]
–	1) 3C‐SiC powder 2) HF/HNO_3_ mixture (40% HF: 65% HNO_3_ (v/v = 3:1) 3) KOH and HCl	3C	0.5–8.5	1) Etching at 85 °C for 6 h 2) Ultrasonication in water 3) pH tuning by preparing SiC QDs suspension including KOH and HCl	[187]
–	1) 3C‐SiC powder (1–10 μm) 2) HF:HNO_3_	3C	1–5	Etching 2 h at 100 °C	[178]
Electrochemical etching	1) n‐type polycrystalline 3C‐SiC wafer 2) HF/ethanol (HF:C_2_H_5_OH = 2:1) 3) DI Water, n‐heptane, dichloromethane	3C	1–4	1) Etching at current density of 30 mA cm^−2^ under UV‐assistance for 60 min. 2) Ultrasonication	[201]
–	1) 4H‐SiC substrate (1 × 2 cm) 2) Platinum as counter electrode 3) Teflon cell as working electrode 4) HF/ethanol	4H	–	1) Etching for 2–3 h (current density = 100 mA cm^−2^) 2) Ultrasonication in DI water for 1 h	[196]
–	1) 3C‐SiC polycrystalline wafer 2) HF/ethanol	3C	1.25–3.5	1) Etching for 3 h under UV irradiation at a current density of 25 mA cm^−2^	[99]
Nonthermal plasma	1) 1.4 sccm SiH_4_ gas 2) 275 sccm Ar gas 3) 150 sccm C_2_H_2_ gas	3C	4.2	1) Generated rf power of 50 W for SiH_4_ and Ar mixture 2) Generated rf power of 150 W for carburized with C_2_H_2_	[106]
	1) HMDS 2) H_2_/Ar plasma gas	3C	3–13	1) Arc current: 0.4 A 2) Flow rate of Ar: 1 slm and H_2_: 10 slm 3) Mass flow of HMDS: 0.4 g/min	[113]
	1) TMS 2) Ar bubbling flow	3C	1.5–5.3	1) Radio frequency (RF) power: 13.56 MHz 2) Total gas flow rate: 1000 sccm 3) Applied RF power: 100 W	[117]
	1) Polycrystalline 3C‐SiC 2) HF‐ethanol (46% HF:ethanol =2:1)	3C	1–6	1) Etching for 60 min in HF‐ethanol at a current density of 40 mA cm^−2^ under halogen lamp 2) Ultrasonication in water for 15 min	[113]
Electrical discharge in liquid	1) Pure silicon, carbon, and tungsten 2) Cyclohexane (CHX) and tetramethylsilane (TMS)	–	≈10	1) Constructing electrodes with Si, C and W with pin configuration 1) Constructing system with CHX and TMS dielectric liquids 2) Applying voltage for spark discharge for 10 min at the repetition rate of 50 Hz	[123]
Ion implantation	1) bulk‐Si substrate 2) SiO_2_ coating 3) C+ ions	3C‐, 8H‐, 6H‐, and 4H‐SiC	2–6	1) Implanting hot carbon ions into bulk Si substrate 2) Post‐annealing with N_2_	[134]
Molten salt electrochemical	1) SiO_2_ powder (15 nm) 2) Carbon powder (10 nm) 3) 15 wt% polyvinyl butyral (PVB)	C	8–14	1) Mixing SiO_2_ and C powder with PVB h 2) Wrapping the pellets into electrodes 3) Constructing the electrolysis cell with prepared cathode, graphite anode and dried CaCl_2_ 4) Applying voltage of 3 V for electrolysis reaction 5) Cooling the halted cathodic products with rate of 2 °C min^−1^ and drying at 100 °C	[138]
Electron beam irradiation	1) 1 g Trimethoxy[3‐(phenylamino)propyl]silane 2) 18 g TX‐100 3) 2 g [BMim]PF_6_ 4) 2 g [OMim]PF_6_	3C and H	7–14	1) Microemulsions preparation with [BMim]PF_6_, water and TX‐100 at a mass ratio of 2:80:18 3) Exposing packaged microemulsions to a 10 MeV electron beam with doses of 50, 80, and 100 kGy	[142]
Laser ablation	1) Nondoped 4H and 6H wafer with thickness of 0.5 mm 2) Acetone	3C 4H	10 30–80	Type of laser: ps Pulse duration: ≈3 ps Wavelength: 800 nm Repetition rate: 1 kHz Scan speed: 100 μm s^−1^ Laser flux: 9.97 J cm^−2^	[148]
	1) Silicon NPs and C NPs 2) Ethanol	3C	15–20	Type of laser: Unfocused ns Nd: YAG Pulse duration: 10 ns Wavelength: 532 nm Repetition rate: 10 Hz Laser flux: 200–600 mJ cm^−2^ Ablation time: 30 min	[150]
	1) Polycrystalline 6H–SiC 2) DI water	3C	≈2	Type laser: ns Pulse duration: 10 ns Wavelength: 248 nm Repetition rate: 10 Hz Laser flux: 320 mJ/pulse Ablation time: 2 h Distance between 6H–SiC and DI water: 8 mm	[149]
	1) 4H–SiC single crystal 2) DI‐water	4H	2.84	Type laser: Femtosecond Pulse duration: 35 femtosecond Wavelength: 800 nm Repetition rate: 1 kHz Scan speed: 0.5 mm s^−1^ Laser flux: 3.5 mJ^−1^ Ablation time: 90 min Distance between 6H–SiC and DI water: 3 mm	[42]
	1) Silicon wafer 2) Ethanol	3C	4	Type laser: ns Nd: YAG laser Pulse duration: 10 ns Wavelength: 1064 nm Repetition rate: 10 Hz Laser flux: 100 mJ/pulse Ablation time: 2 h The spot size of laser beam on target: 2 mm	[153]
	1) 10 mg of SiC powder 2) 30 mL of double distilled water	6H	18–40	Type laser: unfocused ns Nd: YAG Pulse duration: 10 ns Wavelength: 1064 nm Repetition rate: 10 Hz Scan speed: Laser flux: 600 mJ Ablation time: 30 min	[32]
	1) Silicon target 2) 3 mL of ethanol	4H	55–78	Type laser: Nd: YAG laser Pulse duration: 7 ns Wavelength: 532 nm Repetition rate: 1 Hz Laser flux: (1.5–5) J cm^−2^ Ablation time: 15 min The spot size of laser beam on target: 2 mm	[151]
	1) Polished 3C–SiC Polycrystalline with thickness of 3 mm 2) DI water	Amorphous	≈10	Type laser: ns Pulse duration: 10 ns Wavelength: 248 nm Repetition rate: 10 Hz Laser flux: 320 mJ/pulse Ablation time: 2 h	[170]
Electrical discharge + Laser ablation	1) Si and graphite electrodes 2) Ethanol	SiC NPs	2.3	1) Electrical discharge Voltage pulses: 11 kV The peak current: 8 A Pulse duration: 10 μs Electrodes distance: less than 1 mm 2) Laser ablation Type laser: ns Nd:YAG laser Pulse duration: 10 ns Wavelength: 532 nm Repetition rate: 10 Hz Laser flux: 230–600 mJ cm^−2^ Ablation time: 10 min	[125]
Electron irradiation/Laser ablation	1) n‐type 4H‐SiC 2) Milli‐Q water	4H	40–50	1) Electron irradiation: 2 MeV 2) Laser ablation Type laser: femtosecond Pulse duration: 250 femtosecond Repetition rate: 50 kHz Scan speed: 1 mm s^−1^ Laser flux: 1.65 W	[196]
Hydrothermal	1) SiC powders 2) Water	2D 6H QDs	Lateral size: 3 Thickness: 0.5–0.8	1) Reaction time: 12 h 2) Temperature: 140 °C	[158]
Hydrothermal	1) 60 mg SiC powder 2) 30 mL DI water 3) Ammonia 4) 60 mg BSA	SiC@BSA NPs	14	1) Reaction time: 12 h 2) Temperature: 140 3) Dialysis for 3 days 4) Filtration	[159]
Chemical etching/Hydrothermal	1) SiC microcrystallites 2) HF‐HNO_3_ (v/v 3:1)	–	0.67	1) Etching at 100 °C for 1 h. 2) Ultrasonication in water for 20 min 3) Centrifugation 4) Hydrothermal at 180 °C for 10 h	[168]

### 4.1 Top‐down Methods

#### 4.1.1 Chemical or Electrochemical Etching Method

One of the most frequently used methods for the synthesis of ultrafine SiC NCs is chemical or electrochemical etching. In the former, the SiC bulk or powders are exposed to a mixture of nitric and hydrofluoric acids (*V*_HNO3_:*V*_HF_ is mostly 1:3) for a specific duration of time (varied from 1 to 6 h).^[^ ^95^ ^]^ Then, ultrasonication is used to prepare a colloidal suspension of SiC NCs. In the case of bulk samples, an additional energy source in the form of electric current is applied to the bulk SiC under UV irradiation or at high temperatures in HF solution, paving the way for SiC NC formation. Both electroless and electrochemical etching involve electrochemical redox reactions, occurring with or without an applied bias, in reverse order. The following equations represent typical chemical reactions that can be carried out during etching^[^ ^95^ ^]^

(1) $${\text{SiC}+\text{2HNO}}_{3}+{\text{2H}}_{2}\text{O }\to {\text{ 2HNO}}_{2}{\text{ + 4OH}}^{‐}+{\text{SiC}}^{\text{4+}}\;$$

(2) $${\text{4OH}}^{-}+{\text{ SiC}}^{\text{4+}}\;\to {\text{SiO}}_{2}+{\text{CO}}_{2}+{\text{2H}}_{2}$$

(3) $${\text{SiO}}_{2}+\text{ 6HF }\to H_{2}{\text{SiF}}_{6}{\text{ + 2H}}_{2}O$$

However, the porous etching of semiconductors is much more complex and usually happens without any oxide formation.^[^ ^96^ ^]^

The chemical etching of SiC diverges from electrochemical etching in its selectivity for the 3C‐SiC polytype, which alone can be etched using an HF:HNO_3_ solution. Researchers attributed this polytype selectivity to a complex redox reaction involving the injection of both electrons and holes into the semiconductor and called it no‐photon exciton generation chemistry.^[^ ^97^ ^]^ Building on this theoretical framework, a specific etching protocol for 6H‐SiC was developed and successfully applied to achieve porous etching.^[^ ^98^ ^]^

**Figure 7:**
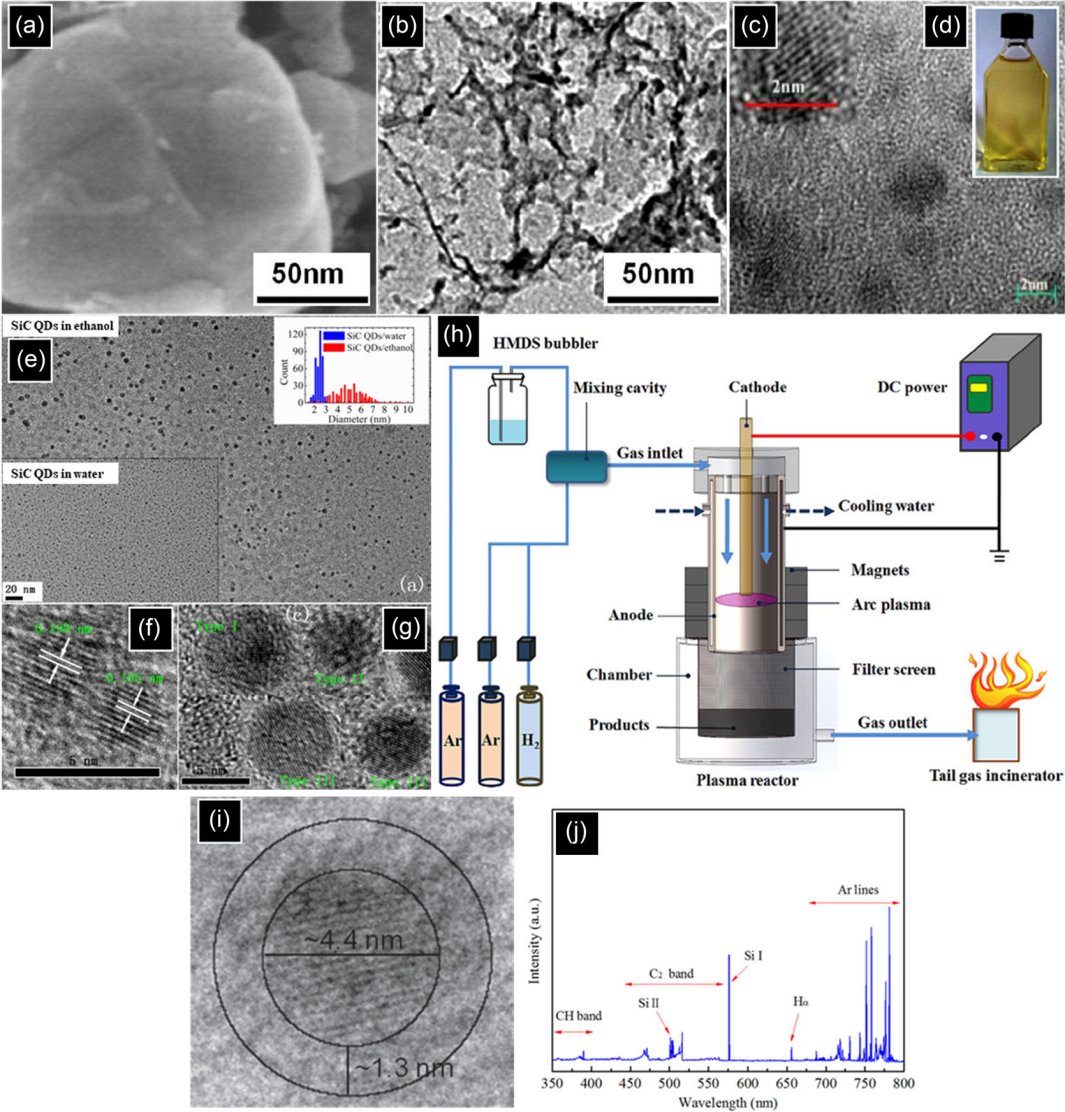
3C‐SiC powders a) before and b) after etching. c) HRTEM and d) photographs of 3C‐SiC NCs aqueous colloidal suspension. Reproduced with permission.^[^ ^95^ ^]^ Copyright 2014, Scientific Research Publishing. e) TEM images and the size distributions of the colloidal suspensions of 3C‐SiC QDs in ethanol and water. HRTEM images of the SiC QDs in f) water and g) ethanol. Reproduced with permission.^[^ ^103^ ^]^ Copyright 2014, AIP Publishing. h) Schematic diagram of the non‐thermal process design. Reproduced with permission.^[^ ^115^ ^]^ Copyright 2021, American Ceramic Society. i) TEM image of a 4.4 nm carbon‐coated 3C‐SiC NC. Reproduced with permission.^[^ ^106^ ^]^ Copyright 2015, IOP Publishing. j) Emission spectrum of arc plasma Reproduced with permission.^[^ ^113^ ^]^ Copyright 2021, Elsevier.

During the electroless etching process, the surface of semi‐spherical SiC powders is corroded, which produces numerous defects and surface distortions resulting in a nanoporous form (**Figure** **7**a,b). By taking advantage of ultrasonication, which produces intense waves, bubbles, and micro‐jets in suspension, the hollow porous particles are broken into smaller pieces.^[^ ^99^ ^]^ As a result, a yellowish suspension is observed, containing 2.5 nm ultrasmall SiC NCs, which can be clearly seen in Figure 7c,d.^[^ ^95^ ^]^ In some cases, it was reported that porous SiC failed to emit light, highlighting the critical role of the ultrasonication treatment in producing fluorescent SiC NCs with various PL emission peaks (400–520 nm). This emission corresponds to either the QCE or surface states, which will be discussed in the next section.^[^ ^100^ ^]^ In addition to ultrasonication processes, dry grinding of the porous structure is also capable of preparing a stable colloidal suspension of SiC NCs below 4 nm.^[^ ^101^ ^]^ In terms of substrate, polycrystalline SiC is preferred over SiC single crystal as it generates smaller NCs. As the volume concentration of grain boundaries increases, the etching process is enhanced. Grain boundaries are defined as low packing density interfaces between two or more grains, which facilitate the corrosion process and result in smaller NCs.^[^ ^100^ ^]^ In terms of surface chemistry and stability of SiC NCs, the high Zeta potential value of ‐29.8 mV from the SiC colloidal suspension is a consequence of Si‐COOH, C‐OH, and Si‐OH dissociation after dispersion of SiC NCs in polar solvents, forming negative electric charges on the surface of NCs, ensuring the long‐term stabilization of NCs.^[^ ^11^, ^93^, ^102^ ^]^

One of the most important factors that influences the final morphology and structural properties of NCs is the solvent used during ultrasonication. Figure 7e shows that the average size and size distribution of SiC NCs ultrasonicated in ethanol (5.0 and 1–10 nm, respectively) are higher than that of the SiC QDs ultrasonicated in water (2.5 and 1–4 nm, respectively).^[^ ^103^ ^]^ In terms of morphology and crystallinity, water‐based ultrasonication produces SiC single crystals, showing lattice spacings of 0.195 nm ((210) plane) and 0.199 nm ((105) plane), related to cubic and hexagonal phases, respectively (Figure 7f). In contrast, ethanol‐based ultrasonication forms a combination of recrystallized single crystal (type III) and an aggregate of 3C‐SiC QDs with different orientations (type I) and compositions (type II) (Figure 7g). Due to the higher polarity of water than ethanol, the suspended SiC QDs in water are highly hydrophilic, being covered with various oxygen‐containing groups, which act as a deterrent against agglomeration.^[^ ^103^ ^]^

Although chemical etching is considered a rapid synthesis method for the preparation of small SiC QDs, it has some drawbacks. First, HF is extremely toxic that can bring about serious health issues, including skin burns, tissue necrosis, and even cancer.^[^ ^104^, ^105^ ^]^ Second, subsequent treatments such as centrifugation and dialysis are required to prepare NCs with a uniform size distribution and remove the etchants from the surface.^[^ ^106^ ^]^ Third, various salts such as NaF and Na_2_SiF_6_ may be deposited on the surface of SiC QDs during the electrochemical etching process, causing an adverse impact on the luminescence efficiency of solid state QD‐based optoelectronic devices.^[^ ^107^ ^]^ Moreover, the initial SiC powders may undergo phase transformation from 4H or 6H to 3C due to the ultrasonication process. For instance, while the phase of the initial precursor was 6H‐SiC, PL results revealed the phase transformation from hexagonal to cubic as the emission tail approached 2.2 eV, which is almost equal to the 3C‐SiC bandgap energy. This phase change is related to ultrasonication waves, providing the system with enough driving force to overcome the phase transformation energy barrier between different polytypes.^[^ ^103^ ^]^

### 4.2 Bottom‐up Methods

#### 4.2.1 Nonthermal Plasma Method

The plasma synthesis method offers a very promising means for the production of a wide range of NCs.^[^ ^108^, ^109^, ^110^, ^111^ ^]^ This ligand‐free process has many advantages, including cost‐effectiveness, scalability, and short processing time. There are two types of plasmas, namely thermal and nonthermal. In the thermal plasma, the precursors are thermally decomposed as the gas temperature significantly increases to high levels above 4000 K.^[^ ^112^ ^]^ As a result, the majority of formed NCs undergo agglomeration, broadening the size distribution of NCs. This phenomenon mainly stems from the lack of ability of the thermal plasma process to produce well‐separated, charged NCs. However, in the case of nonthermal plasma, while the temperature of the generated plasma electrons reaches above ≈10 000 K, the temperature of heavy gas species remains low (less than 3000 K).^[^ ^113^ ^]^ In this case, the hot electrons collide with molecules, dissociate and ionize gaseous precursors, and produce highly reactive radicals and ions for the nucleation and growth of NCs. Moreover, following the exothermic reaction between the radicals and the surface of NCs, intense heat is generated, which improves the crystallinity of NCs by elevating the surface temperature. Compared to the case of the thermal plasma, negatively charged NCs are formed in non‐thermal plasmas, which lessens the agglomeration and tightens the size distribution.^[^ ^114^ ^]^

The schematic diagram shows the design of the nonthermal arc plasma process in Figure 7h. The main aspect is the plasma reactor, comprised of coaxial electrodes and several annular magnets surrounding the outer electrode. The magnets apply an axial magnetic field and rotate the arc plasma around the inner electrode (100–500 Hz). The precursors and plasma gases are transferred to the reactor by a bubbler, controlling the flow and concentration of precursors in the plasma. Finally, an electrical power supply provides the volume gas with a constant (DC) or time‐varying (radio or microwave frequency) electric field. The process is initiated by purging Ar into the plasma region and applying the electric field through the gas at atmospheric or low pressure, resulting in strong electric discharge between the inner (cathode) and outer (anode) electrodes. After reaching steady state, the precursors flow into the plasma zone, followed by decomposition, nucleation, NC growth, and eventually deposition of NCs onto a collection chamber, Si wafer, or filter screen, and the rest of the gases are released through the gas outlet.^[^ ^114^ ^]^

Recently, the nonthermal plasma process has been a key area of research focus for the synthesis of well‐crystallized fluorescent SiC NCs. To achieve this, various parameters were identified and optimized. Among all, plasma reactor design and materials selection play the most important roles. For instance, as is seen in Figure 7i, by the separate injection of the mixtures of SiH_4_/Ar and C_2_H_2_/Ar gases into upstream and downstream plasma regions of a plasma reactor, respectively, SiC NCs were synthesized with a size of 4.2 nm coated with hydrogenated amorphous carbon (a‐C:H) with a thickness of ≈1.3 nm.^[^ ^106^ ^]^ When either plasma region was turned off and gases were co‐introduced, no 3C‐SiC powder was observed, demonstrating the importance of design in successful NC generation. Based on the proposed mechanism, crystalline Si NCs are formed in the upstream plasma region, followed by the complete carburization of NCs in the downstream plasma region, resulting in 3C‐SiC NC formation. Another attractive approach is to employ a precursor that simultaneously contains both carbon and silicon atoms. Hexamethyldisilane (HMDS) has been introduced as one of the promising liquid‐based silicon/carbon containing candidates to produce near‐spherical 3C‐SiC NCs)8 nm in diameter) under atmospheric pressure.^[^ ^113^ ^]^ As nonthermal plasma is a bottom‐up process, Si and C bond to prepare 3C‐SiC as the most stable polytype. In another paper, it was revealed that tetramethylsilane (TMS) has the ability to synthesize smaller QDs with a size range of 1–5 nm at atmospheric pressure.^[^ ^113^ ^]^ The strong XPS peaks of Si 2*p* at 100.1 eV and 101.0 eV, related to low‐ and high‐coordinated Si‐C bonds, confirmed the formation of SiC NCs. In addition, in terms of surface chemistry, a strong peak at 99.2 eV showed that QDs were mostly terminated by Si‐H, and the oxidation was substantially inhibited as evidenced by the weak intensities of O‐Si‐C and Si‐O bonds at 101.6 and 102.4 eV, respectively.^[^ ^113^ ^]^

**Figure 8:**
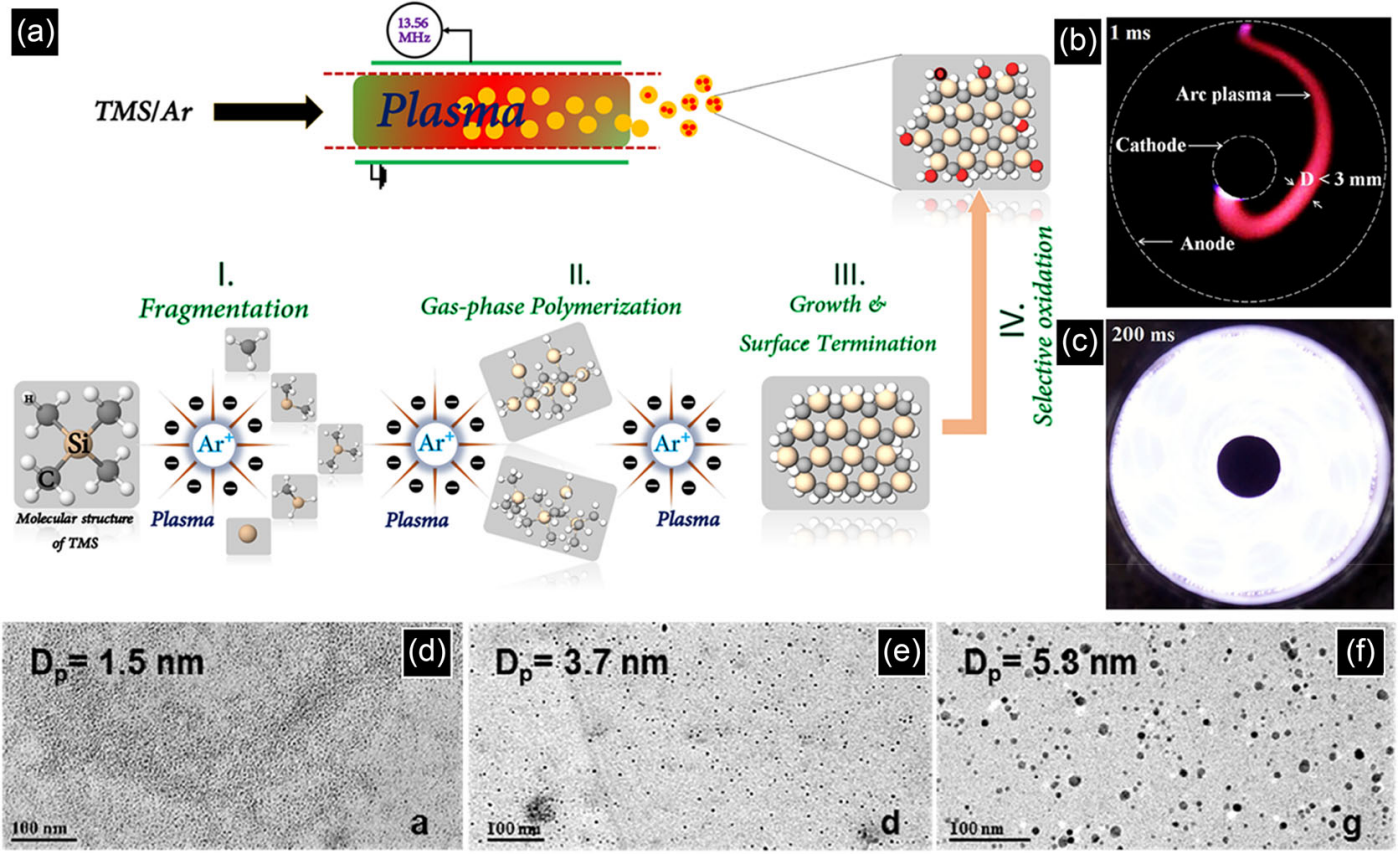
a) Schematic diagram showing the synthesis process of 3C‐SiC NCs via nonthermal plasma. Reproduced with permission.^[^ ^29^ ^]^ Copyright 2021, American Chemical Society. b,c) The effect of exposure time on the arc plasma shape. Reproduced with permission.^[^ ^115^ ^]^ Copyright 2021, American Ceramic Society. TEM images of 3C‐SiC NCs prepared at precursor flow rates of d) 0.4 sccm, e) 2.4 sccm, and f) 5 sccm. Reproduced with permission.^[^ ^117^ ^]^ Copyright 2016, Royal Society of Chemistry.

The mechanism of SiC QDs formation from Si and C‐containing precursors can be described in **Figure** **8**a. First, the interactions between hot electrons and precursor molecules lead to excitation, dissociation, and ionization, which generate a variety of reactive radicals.^[^ ^115^ ^]^ This can be supported by the emission spectra of the plasma when HMDS and Ar gases were used. Figure 7j shows the presence of CH/C_2_ molecules, Si/H atoms, excited Ar atoms, and hot electrons, indicating the decomposition of HMDS and Ar transformation.^[^ ^115^ ^]^ Therefore, neutral precursors like TMS lose their methyl groups and form numerous TMS fragments, followed by the gas‐phase polymerization, nucleation, and growth of a Si−C network with H terminations on the carbon atoms.^[^ ^116^ ^]^ The formation of Si—C bond is preferable as this reaction has a lower Gibbs free energy compared to that of C to C.^[^ ^113^ ^]^ Finally, the required crystallization energy is provided by bombarding and selectively heating NC surfaces to hundreds of kelvin higher than the surrounding gas‐phase by hot electrons during the growth.^[^ ^29^ ^]^ In the final step, in some cases, surface Si atoms undergo oxidation due to the weak bonding between Si atoms and methyl groups. It is worth mentioning that if the plasma fails to fully dissociate TMS and atomize Si, instead of preparing a Si—C bond network, Si—C—C—Si networks are formed, changing the band structure and chemical composition of SiC.^[^ ^117^ ^]^

Atmospheric plasma processes could also be used for the synthesis of doped SiC NCs. For instance, by using Ar, HMDS, and N_2_ as the plasma (or carrier), silicon/carbon, and nitrogen sources, respectively, N‐doped SiC QDs were prepared, as confirmed by the appearance of Si—N and C—N bonds in FTIR tests. Apart from the outstanding impact of N_2_ on optical properties, simply adding N_2_ to Ar resulted in NCs size enlargement from 5–20 to 30–200 nm, affecting the structural properties.^[^ ^118^ ^]^

The crystallinity of SiC NCs can be tuned by changing the synthesis parameters. For instance, although the microwave plasma method was capable of producing 5 nm SiC nanoparticles (NPs) from a TMS precursor, X‐ray diffraction (XRD) results revealed that the phase of the SiC product was amorphous.^[^ ^118^ ^]^ Failing to prepare crystalline SiC NCs makes the process complex and energy consuming, as further treatment at high temperatures (at 800 °C for 30 min) is required to modify the crystallinity of the products. One of the ways to address this problem is to change the arc current. When the arc current was 0.1 A, a broad diffuse ring in the SAED pattern was observed; however, increasing the arc current to 0.3 A led to the appearance of concentric rings, showing the formation of amorphous and crystalline SiC NPs, respectively.^[^ ^119^ ^]^ In the aspect of gases, H_2_ injection was also found effective in enhancing the crystallinity of the SiC as the XRD diffraction peaks of SiC NCs were narrowed and the intensities increased.^[^ ^115^ ^]^ However, the introduction of N_2_ to the chamber weakened the 3C‐SiC XRD diffraction peaks, showing a decrease in β‐SiC crystallinity.^[^ ^118^ ^]^

To change the size of QDs, the relevant parameters should be tuned. One of the most determining factors is the precursor's residence time in the plasma region, altering the plasma shape. As is seen in Figure 8b,c, when the arc plasma exposure time changes from 200 to 1 ms, the plasma region transforms from a disk shape to a slender structure. The shorter exposure time of arc plasma decreases the plasma treatment area and lowers the growth driving force, which decreases the size of NCs.^[^ ^113^ ^]^ Another important parameter is the flow rate of precursors into the bubbler. By increasing the flow rate of TMS through the bubbler from 0.4 to 5.0 sccm, not only does the average size of NCs increase from 1.5 to 5.3 nm, but also the size distribution becomes larger (Figure 8d,e,f). The lower the flow rate, the lower the concentration of TMS, minifying the produced NCs.^[^ ^117^ ^]^ On one hand, this improves the optical properties of NCs since the measured PLQY for the deposited NCs with sizes of 1.5, 3.7, and 5.3 nm were 13.2%, 8.4%, and 2.6%, respectively. On the other hand, the larger NCs showed a broader emission band, covering almost the entire visible region that can be used for white‐light emission in solid‐state lighting.^[^ ^117^ ^]^ Purging H_2_ into the plasma region was also found to be effective in size tuning. For instance, although the average size of SiC NCs was measured as 8.3 nm in the absence of H_2_, the particle sizes decreased to 7.1 and 5.6 nm when the H_2_ flow rate increased to 3 and 6 slm, respectively. In addition, compared to the costly microwave plasma process, arc plasma benefits from inexpensive components and also a shorter plasma plume, decreasing the residence time of precursors in the plasma region, and achieving a smaller size of QDs.^[^ ^115^ ^]^

In contrast to the etching process, the surface of QDs formed by nonthermal plasma is generally covered with various coatings, especially carbon. The main reason behind this observation corresponds to the high carbon/silicon ratio of HMDS with respect to SiC. Specifically, after the consumption of the available Si atoms, the excess carbon is distributed on the surface of SiC cores, forming a core–shell structure of 3C‐SiC/graphite (carbon layers).^[^ ^106^, ^113^ ^]^

**Figure 9:**
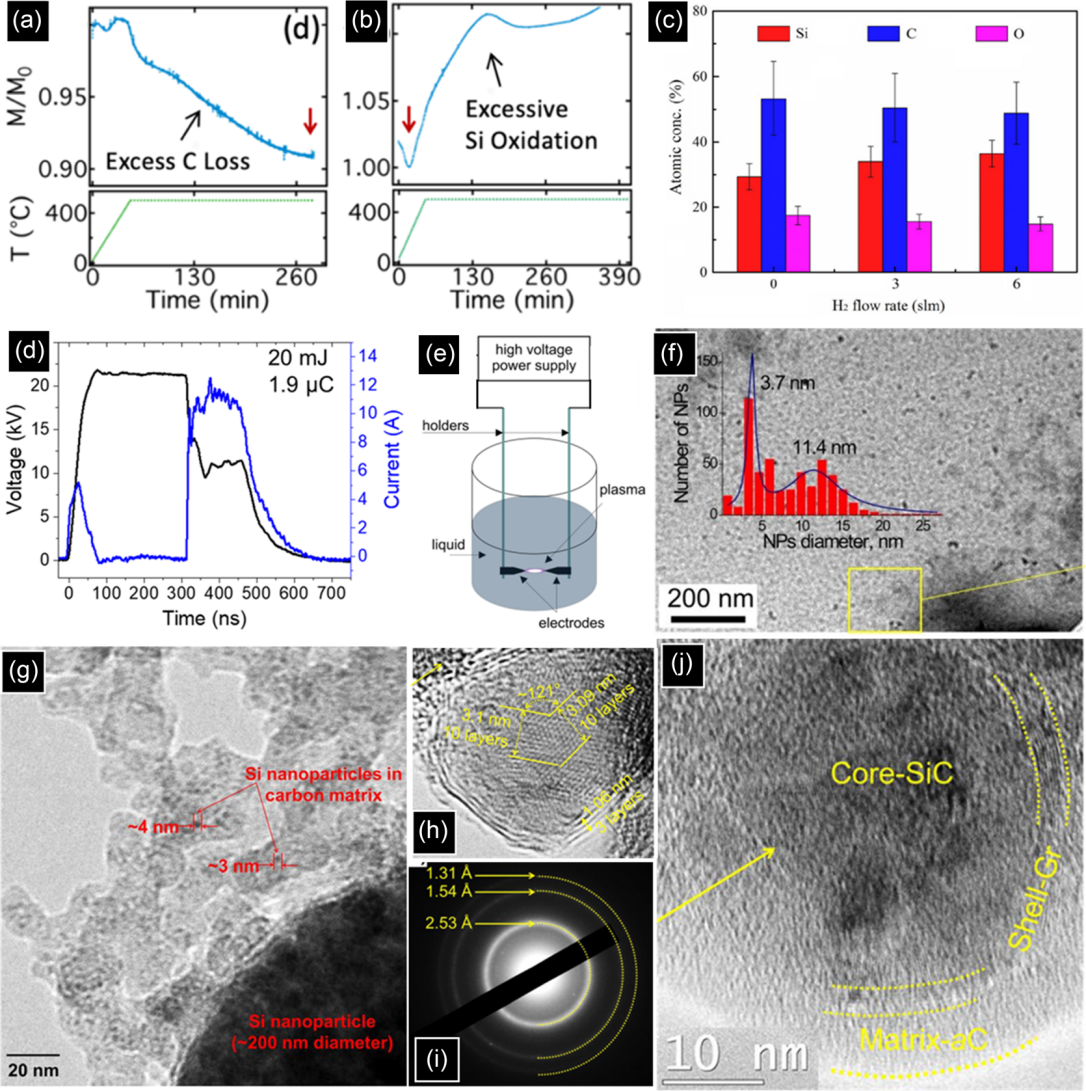
TGA of 3C‐SiC NCs a) baked for excess carbon removal and b) baked for silicon oxidation. Reproduced with permission.^[^ ^120^ ^]^ Copyright 2022, American Chemical Society. c) Atomic concentrations of Si, C, and O of 3C‐SiC NCs in the presence and absence of H_2_. Reproduced with permission.^[^ ^115^ ^]^ Copyright 2021, American Ceramic Society. d) The voltage and current waveforms for one discharge at 22 kV/500 ns. Reproduced with permission.^[^ ^124^ ^]^ Copyright 2021, Springer Nature. e) Schematic diagram of electrical discharge setup for synthesis of SiC NCs. f) TEM image of 3C‐SiC NCs formed by the electrical discharge process. Reproduced with permission.^[^ ^125^ ^]^ Copyright 2022, Springer Nature. TEM images of particles formed by discharge between g) Si—C electrodes in CHX at 22 kV/500 ns h) C—C electrodes in TMS at 22 kV/500 ns, i) SAED pattern of NPs formed by discharge between C—C electrodes in TMS at 22 kV/500 ns, and j) C—C electrodes in TMS at 8 kV/100 ns. Reproduced with permission.^[^ ^124^ ^]^ Copyright 2021, Springer Nature.

To modify the surface coatings of SiC NCs, a few approaches can be adopted. One of the most effective ways is thermal treatment at high temperatures in an air atmosphere. It was revealed that by varying the temperature to 400, 800, and 1200 °C, C‐coated SiC NCs were transformed into SiC core/carbon‐SiO_2_ shell, SiC core/thicker SiO_2_ shell, and SiO_2_, respectively. According to high‐resolution Si 2*p* spectra, Si—C bonds are dominant in untreated samples; however, by increasing the temperature to 1200 °C, the Si—O bonding peak is intensified and the Si—C bonding peak is significantly diminished, confirming the formation of oxidized NPs.^[^ ^113^ ^]^ In another survey, baking time was optimized to protect the SiC NCs against oxidation.^[^ ^120^ ^]^ To monitor residual carbon shell removal, the samples were annealed at 500 °C in air via thermogravimetric analysis (TGA). As is seen in **Figure** **9**a, the TGA mass loss is stopped when the mass reaches the lowest amount to prohibit further oxidation (Figure 9b), even though there is a possibility of the formation of a very thin oxide layer (0.5 nm).^[^ ^120^ ^]^ Another shell removal strategy is purging hydrogen in the plasma region to not only reduce carbon but also oxygen. As is seen in Figure 9c, in the absence of H_2_, the silicon to carbon atomic ratio is 0.55. By increasing the H_2_ flow rate to 6 slm, this atomic ratio increased to 0.75. In addition, both carbon (C—C/C═C) and oxygen contents decrease, showing H_2_ is capable of removing the carbon layer as well as diminishing the surface oxidation of SiC NCs.^[^ ^115^ ^]^ In another study, mass spectroscopy was employed to discover the mechanism behind the positive effect of H_2_ gas.^[^ ^121^ ^]^ It was revealed that in the absence of H_2_, TMS is decomposed into methyl and hydrogen radicals (CH_3_^•^ and H^•^), followed by ethane, a small amount of methane, and hydrogen gases. However, after purging H_2_ gas, the pressure of methane significantly increased, and hydrogen‐rich products such as C_2_H_4_ and C_2_H_2_ were detected. This was related to abundant and highly reactive H radicals originating from the H_2_ gas, attacking TMS to reduce the carbon by the formation of various reaction product gases. Therefore, this substantial consumption of carbon in TMS leads to a lower C/Si atomic ratio in the SiC NCs.^[^ ^121^ ^]^

#### 4.2.2 Electrical Discharge in Liquid Method

Electrical discharge in liquid is another rapid and simplified fabrication process that has been capable of synthesizing a variety of semiconductor NCs, especially SiC.^[^ ^122^ ^]^ As schematically shown in Figure 9e, the setup is comprised of two facing Si and C electrodes with a controllable specific distance (typically less than 1 mm) immersed in a dielectric liquid (solvent). The required driving force for spark discharges in the dielectric liquid is provided with high voltage pulses (above 10 kV) with variable frequencies and widths.^[^ ^123^ ^]^ Figure 9d shows the voltage and current waveforms for only one discharge. As a result of rapid voltage change, two current peaks are observed during the rising and falling periods. When the voltage abruptly drops from ≈22 to ≈11 kV, a current peak of ≈12 A appears and results in an injected charge of ≈1.9 μC with an energy of ≈20 mJ.^[^ ^124^ ^]^ This induces the evaporation of electrode surface atoms as well as liquid dissociation, making a cloud of reactive species between the electrodes. Due to the presence of elevated temperature and high pressure in the plasma, the plume of evaporated Si and C atoms interact with each other, followed by nucleation and growth. The growth is stopped when the particles move out from the plume into the liquid. For instance, by taking advantage of electrical discharge between the immersed Si and graphite electrodes in ethanol, near‐spherical SiC NCs were prepared. The XRD peaks at 35.7° and 60.4° indicated the formation of 3C‐SiC NCs by this bottom‐up method. Apart from the SiC peaks, XRD detected other peaks related to cubic Si and hexagonal graphite phases, showing one of the disadvantages of this process in the preparation of pure SiC QDs.^[^ ^125^ ^]^ Moreover, as is seen in Figure 9f, there are two size distributions of 3.7 and 11.4 nm, indicating the incapability of this method to synthesize monodisperse SiC NCs.

To address this problem, among various parameters, including voltage, discharge gap width, and electric pulse frequency, influencing the structural and chemical properties of NCs, materials selection plays the most important role. In terms of solvents, although water induces NC oxidation, liquid hydrocarbons like ethanol protect the NCs against oxidation. In another study, the effect of electrode materials (graphite and silicon) and dielectric liquids (cyclohexane (CHX) and TMS) on the structural and compositional properties of NCs was assessed.^[^ ^124^ ^]^ When Si—C electrodes in CHX at 22 kV/500 ns (voltage pulse amplitude/pulse time) were used, large Si particles (200 nm) and very small Si particles (4 nm) embedded in an amorphous carbon matrix were formed (Figure 9g). In the case of Si—Si electrodes in CHX at 22 kV/500 ns, in addition to large Si particles in a shell of carbon (200–300 nm), core–matrix structures comprised of 10–20 nm Si NCs in a film of C were produced. The absence of the SiC and formation of Si NCs in these cases were related to the short time scale of the very fast discharge, failing to prepare a stable plasma of Si and C constituents. However, after discharges in TMS between two C electrodes at 22 kV/500 ns, large particles disappeared, and only 10 nm NPs encapsulated in a graphite shell were formed (Figure 9h). According to the SAED pattern in Figure 9 **i**, the interplanar distances are attributed to (111), (022), and (113) planes of SiC, showing the formation of pure SiC NCs. Apart from the solvent and precursors, the width and amplitude of the voltage peak also have great potential to modify the structural properties of SiC NCs. Although applying a lower voltage (8 kV instead of 22 kV) and a shorter pulse width (100 ns instead of 500 ns) are still capable of producing SiC NCs, based on EDX spectra, they decrease the intensity ratio of Si/C peaks from 2.75 to 0.55, resulting in a carbon‐rich SiC core with a graphitic shell of 6–8 monolayers embedded in an amorphous carbon film (Figure 9j).^[^ ^124^ ^]^

Researchers also used other designs to synthesize SiC NCs. For instance, instead of using Si and carbon electrodes, a cylindrical insulating quartz tube is reported whose outer surface is tightly surrounded by copper wire as the external electrode, and heating is via an electric soldering iron core that forms the inner electrode. Various precursors, including (3‐aminopropyl trimethoxysilane) APTMS, ultrapure water, H_2_O_2_, and oxygen gas, were transferred to the quartz container, followed by applying an input voltage of 50 V for 1.5 h. When the discharge breaks through the medium, oxygen molecules are electrolyzed into oxygen atoms, and as a result of a reaction between the oxygen molecules and atoms, O_3_ molecules are generated. Then, H_2_O_2_ and O_3_ were consumed and formed numerous ^•^OH (hydroxyl radicals) under alkaline conditions which play the most important role in the SiC NCs generation (Equation (4)**–**(11)). Then, the generated ^•^OH first reacts with methanol (CH_3_OH), produced from the hydrolysis of APTMS, followed by an attack on the silane's organic chain to produce SiC NPs with an average diameter of 20 nm. XPS showed that by taking advantage of APTMS, the surface of NCs was passivated with a variety of advantageous functional groups, including N═O, N—H, C═O, and C—O, enabling a water‐soluble product.^[^ ^126^ ^]^

(4) $$O_{2}\text{ + e}\to \text{2O + e}$$

(5) $$O_{2}\text{ + O}\to O_{3}$$

(6)

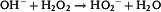



(7)

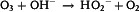



(8)

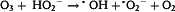



(9)

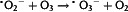



(10)

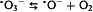



(11)

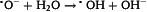




#### 4.2.3 Ions Implantation Method

SiC synthesis methods are generally ex situ, meaning that the resulting NCs require further deposition techniques such as drop casting, spin coating, and centrifugation for the fabrication of solid‐state optoelectronics.^[^ ^127^, ^128^, ^129^ ^]^ In contrast, ion implantation is considered an in situ process as it is capable of growing SiC QDs in one step inside the substrate. For instance, 2 nm SiC QDs with a density of 2 × 10^12^ cm^−2^ were formed inside SiO_2_ by double ion implantation of Si^+^ (dose: 6 × 10^16^ cm^−2^ with acceleration energy (AE) of 60 keV)/C^+^ (dose: 4 × 10^16^ cm^−2^ with AE of 25 keV) at temperatures between 200–400 °C. Despite several advantages, the QDs suffer from low crystallization, creating numerous dangling bonds in the form of vacancies inside SiC QD and at the SiO_2_/SiC QD interface, adversely influencing the optical properties. To address this problem, various parameters were assessed and optimized. The first factor was N_2_ treatment at high temperatures between 900 and 1100 °C. Based on the atom probe tomography (APT) results of plane view distributions of Si (blue dots), C (yellow dots), and N atoms (red dots) of SiC‐QD at the 80 nm depth from the oxide surface, N_2_ annealing was more effective in increasing the PL intensity of QDs compared to argon annealing, due to the reduction of dangling bond density by passivating N atoms at SiO_2_/QD interfaces and inside the QD.^[^ ^130^ ^]^ Increasing N_2_ annealing time up to 30 min also leads to PL intensity improvement. However, PL intensity takes a downward trend after longer N_2_ annealing, corresponding to Si—C bond decomposition.^[^ ^131^ ^]^ The substrate bandgap energy also plays an important role. For instance, the PL quantum efficiency of SiC QDs formed in silica was ≈2.5 times greater than that of SiC QDs in Si.^[^ ^132^ ^]^ This is mainly because the bandgap energy of SiC QDs is remarkably higher than that of Si but lower than SiO_2_, resulting in a higher QCE in the SiC/SiO_2_ heterojunction that forms.^[^ ^133^ ^]^ The diameter (*D*) and surface density (*N*_D_) of implanted SiC NCs can also tune the luminescence intensity. To investigate this, C^+^ ions were introduced into bulk silicon having (110), (111), and (100) orientations with surface densities (*N*_S_) of 5.65, 6.92, and 8, respectively. It was revealed that *D* and *N*_D_ decreased with increasing *N*_S_ as the number of C‐ions converting to SiC QDs in deeper areas is reduced. As a result, this improves the PL emission, probably related to the crystal quality improvement by reduction of dislocation concentration in SiC‐dot in smaller *D* conditions.^[^ ^134^ ^]^

An interesting synthesis route that results in similar embedded SiC nanocrystals is the reaction of Si with CO.^[^ ^135^, ^136^ ^]^ When a Si wafer is used as a Si source, SiC nanocrystals with facet‐dependent size and morphology are formed on the Si surface. However, no PL is reported from these NPs.

#### 4.2.4 Molten Salt Electrochemical Method

**Figure 10:**
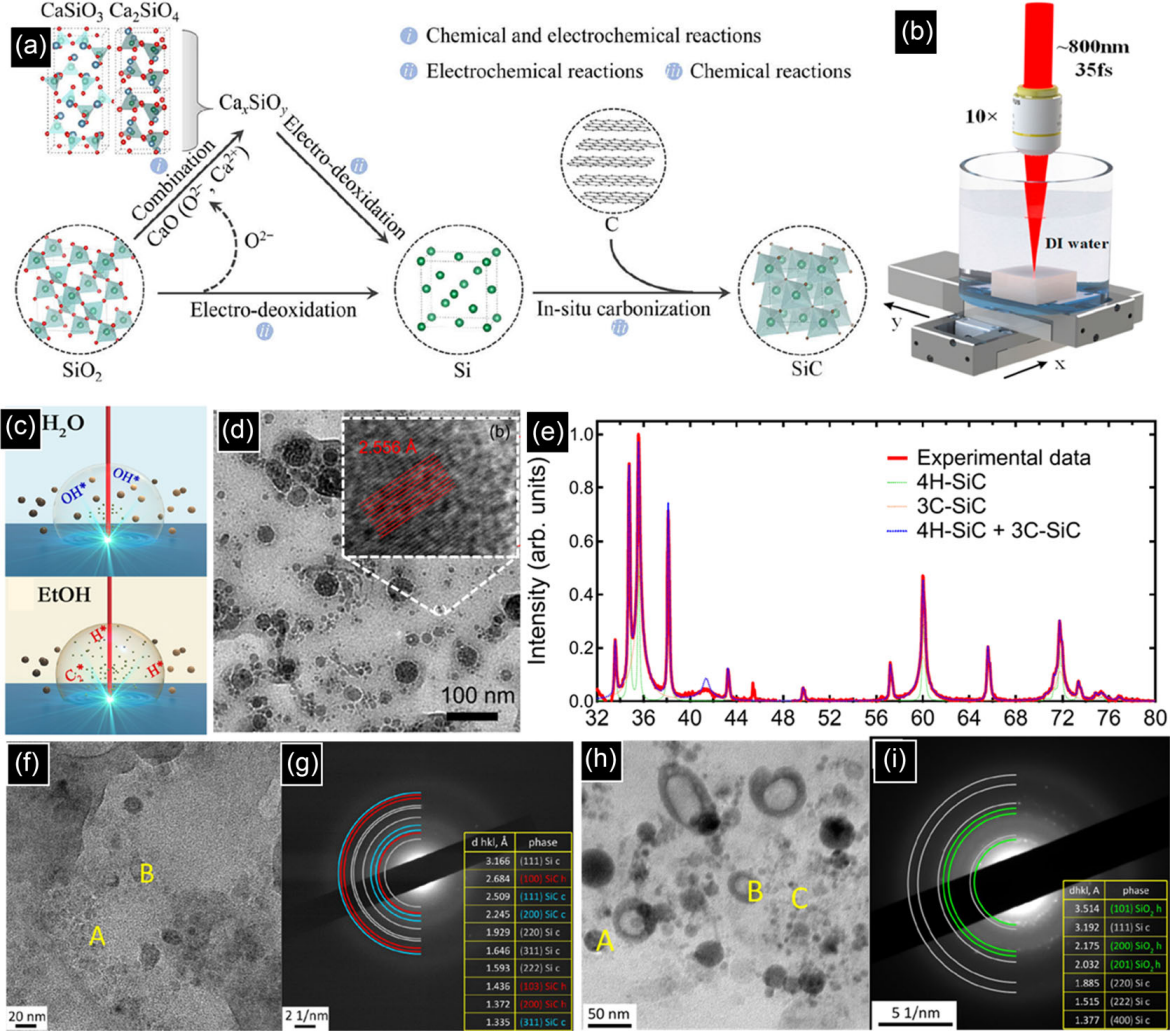
a) Schematic diagram of molten salt electrochemical mechanism for 3C‐SiC NPs formation. b) Experimental setup for the PLA in liquid. Reproduced with permission.^[^ ^140^ ^]^ Copyright 2022, Elsevier. c) Schematic diagram of the ablation process at the interface of 6 H‐SiC and different solvents. Reproduced with permission.^[^ ^152^ ^]^ Copyright 2015, American Chemical Society. d) TEM image of NCs prepared by ablating the 4 H‐SiC substrate with the corresponding lattice spacing. e) XRD spectra for the SiC NCs formed by laser ablating of a 4 H‐SiC substrate with a fluence of 1.67 J cm^−2^. Reproduced with permission.^[^ ^148^ ^]^ Copyright 2019, AIP Publishing. f) TEM image of NCs prepared by nanosecond laser irradiation. g) SAED pattern of the selected region. h) TEM image of NCs prepared by femtosecond laser irradiation. i) SAED pattern of the selected region. Reproduced with permission.^[^ ^150^ ^]^ Copyright 2022, Springer Nature.

Molten salt electrolysis is an effective process that has been capable of producing metal/alloy/semiconductor materials from their metal oxides.^[^ ^137^ ^]^ This process benefits from low temperatures (700−900 °C) and low‐cost oxides as raw materials, making the process more energy‐saving. By applying a constant potential of 3.0 V between a Ni‐wrapped SiO_2_/C cathode and graphite anode in a CaCl_2_ electrolyte placed in an electrolytic furnace at 900 °C, fluorescent β‐SiC NPs with a strong emission peak at 410 nm were formed. In some cases, an emission peak was also observed at 534.2 nm, related to the dislocations, extended defects, and irregular structures of SiC.^[^ ^138^, ^139^ ^]^ The mechanism of SiC QDs formation can be explained as follows. As is schematically shown in **Figure** **10**a, due to the spontaneous reaction between SiO_2_ and CaO, Ca_*x*_SiO_*y*_ is formed, followed by Si formation as a result of the electrochemical deoxidation of SiO_2_ and Ca_*x*_SiO_*y*_. Then, an in situ carbonization reaction between the resultant Si and C produces SiC NPs.

One of the main advantages of liquid molten salt is its high polarity and surface tension, preventing the particles from agglomeration.^[^ ^140^ ^]^ In addition, it was revealed that elevating the temperature from 1250 to 1350 °C increases the PL intensity. This was attributed to the lower bandgap energy of NPs formed at higher temperatures, enhancing the concentration of photoexcited carriers.^[^ ^138^ ^]^

#### 4.2.5 Electron Beam Irradiation Method

Electron beam irradiation of ionic liquid‐based microemulsions is a simple, fast, and safe process for the preparation of stable NCs.^[^ ^141^ ^]^ In addition, this process is capable of tuning the size of NCs. Very recently, SiC NCs were produced by applying various doses of a high‐energy electron beam (10 MeV) to a microemulsion comprised of 1‐butyl‐3‐methylimidazolium hexafluorophosphate ([BMim]PF_6_), trimethoxy[3(phenylamino)propyl] silane, water, and TX‐100. Observed Raman and FTIR peaks at 973 and 734 cm^−1^, related to longitudinal optical (LO) and transverse optical (TO) of SiC, respectively, confirmed the formation of SiC NCs. HRTEM also revealed that this bottom‐up process produced a cubic phase, as the measured lattice fringe was attributed to the (111) plane of β‐SiC. Similar to other methods, this process enables the surface passivation of SiC NCs with a variety of functional groups, including C═O, C—O, and Si—O—Si groups.^[^ ^142^ ^]^

#### 4.2.6 Pyrolysis Method

By taking advantage of the pyrolysis of dodecamethylhexasilinane tablets (2.5–3 mm thick and 6 mm in diameter) at high pressure and temperature (800 °C), tiny 3C‐SiC NCs less than 2 nm in diameter were formed. It was revealed that the processing temperature should be carefully optimized, as elevating the temperature to 1400 °C increased the size of NCs to 500 nm due to the recrystallization driven by a decrease in the surface energy of the particles.^[^ ^143^ ^]^

The last three synthesis methods were recently introduced, requiring more investigations to be optimized.

### 4.3 Top‐down and Bottom‐up Methods

#### 4.3.1 Pulsed Laser Ablation Method

Pulsed laser ablation (PLA) is one of the most effective methods for the large‐scale production of SiC NPs.^[^ ^144^, ^145^, ^146^ ^]^ This process, which benefits from a facile experimental setup and safe chemical precursors, is capable of producing very tiny NPs with a uniform size distribution. Moreover, compared to electrochemical etching, laser ablation was found to be more effective in producing a highly stable colloidal suspension of SiC NCs,^[^ ^93^ ^]^ confirmed by a higher zeta potential value at −46 mV than −29.8 mV, stemming from numerous surface‐functionalizing carboxylate anions (CO_2_^−^) on the surface of SiC NCs.^[^ ^147^ ^]^ As shown in Figure 10b, the PLA setup is comprised of a pulsed laser system, a glass beaker, solvent, and a SiC target.^[^ ^140^ ^]^ In terms of solvent selection, similar to the electrical discharge process, ethanol is preferred. The SiC target can possess different polytypes (3C, 4H, and 6H), various morphologies from bulk to powders, and different types of dopants. The process is carried out by sweeping a focused or unfocused laser beam across the specific area of the target to ablate the material from the surface. After a specific growth duration, the color of the solvent changes, mainly becoming brownish, showing the NP formation in the solvent. Finally, a fresh and purified colloidal suspension of NPs may be obtained, typically using centrifugation. PLA can be considered a bottom‐up or a top‐down method. When NP preparation requires nucleation and growth steps, the process is called bottom‐up. In this case, laser energy is transmitted to the SiC lattice through both electron‐electron collisions involving the thermalization of electrons with a time constant of ≈ 500 femtoseconds and electron‐phonon collisions with a time constant of ≈10 ps. The result is a high‐temperature and pressure plasma at ≈6000 K and 1 GPa, respectively. The plasma is composed of ions, atoms, and molecules. When the plasma expands, the surrounding liquid compresses and cools the plasma by transmitting the energy from the lattice to the surrounding solvent by phonon−phonon coupling, with a time constant of ≈100 ps. At this moment, due to the rapid cooling, very small SiC QDs are nucleated and grown. In contrast to the above bottom‐up process, when the fragments are directly detached from the target and the initial target phase is preserved, the process is properly considered a top‐down process.^[^ ^140^ ^]^

The mechanism and characteristics of NCs formed by top‐down and bottom‐up processes can be explained in more detail in the following investigation. When a picosecond laser was used to ablate a 4H‐SiC bulk target, two size ranges of small and large NPs with different compositions were formed.^[^ ^148^ ^]^ HRTEM showed that smaller particles less than 10 nm had a spherical shape (Figure 10d). However, the larger particles with diameters more than 30 nm had an angular morphology. Being spherical is one of the characteristics of particles grown by a bottom‐up procedure, as they prefer to have the lowest surface‐to‐volume ratio, while the irregular shape is a representative of being a fragment from the target, showing the top‐down process. More importantly, based on XRD results in Figure 10e, in addition to the peaks at 33.6° and 43.3°, attributed to (100) and (103) planes of 4H‐SiC peaks, there are other two peaks at 35.6° and 60.0°, corresponded to either (004) and (110) planes of 4H‐SiC or (110) and (220) planes of 3C‐SiC. While the large particles have a hexagonal crystal structure, the smaller particles, less than 10 nm, were proven to be 3C‐SiC by two observations in XRD. First, the intensity ratios between these peaks and the peak of 4H‐SiC at 43.3° were not consistent with the theoretical intensity ratios of 4H‐SiC. In addition, XRD results showed that the line widths of these two peaks are broader, indicating that the formed particles experienced large lattice distortions. This was quantitatively confirmed by fitting the XRD results, as the lattice distortion of the smaller NPs was 0.7%, which was 0.6% higher than that of the larger particles. This larger distortion is a consequence of the rapid growth of spherical QDs from the dissociated ions and atoms in the bottom‐up process. As 3C‐SiC is the most thermodynamically stable polytype, the rearrangement of atoms in the form of the cubic phase is more favorable in the bottom‐up method. In another survey, PL tests revealed that 2‐5.5 nm NCs formed by ns‐laser irradiating a polycrystalline 6H‐SiC substrate in deionized water (DI water) were 3C‐SiC. When the suspension was illuminated under the excitation wavelength range of 260–420 nm, the PL peaks were located at 413–495 nm (3.00–2.51 eV), very close to the bandgap of bulk 6H–SiC. However, due to the QCE, the emission wavelength of 6H‐SiC should have appeared at 3.83 eV.^[^ ^149^ ^]^ To confirm the phase was 3C‐phase, the effective mass/Rydberg approximation was used, showing the relation between the bandgap energy and QDs size as follows

(12) $$E_{g}\text{(QD) = }E_{g}\text{(bulk) + }\frac{h^{2}}{8\mu r^{2}}\;-\frac{1{\text{.8e}}^{2}}{4\pi \varepsilon_{0}\varepsilon_{r}}$$

By applying the values of 3C–SiC bulk bandgap energy (2.2 eV), reduced mass of exciton (*μ*), Planck's constant (*h*), permittivity of vacuum (*ε*_0_), size‐dependent dielectric constant (*ε*_r_), and fitted PL peak position of 395 nm (3.14 eV) at excitation wavelength of 320 nm into the above equation, the radius was calculated as 1.32 nm, which was similar to the radius of QDs observed in TEM, showing the 3C‐SiC QDs were formed from 6H‐SiC substrate.

To achieve the formation of pure 4H or 6H‐SiC QDs without 3C‐SiC QDs, the type of laser plays an important role. Despite the nanosecond and picosecond pulsed lasers, which form 3C‐SiC QDs or a mixture of cubic and hexagonal structures, Guo et al.,^[^ ^150^ ^]^ succeeded in synthesizing 4H SiC NPs from a target with a 4H structure by using a femtosecond laser system in deionized water. Femtosecond pulse lasers facilitate the direct ionization of the target and greatly reduce the thermal effect, resulting in the removal of the transformation probability from 6H and 4H SiC to 3C–SiC. In contrast to this, for nanosecond or even picosecond pulse duration lasers, the pulse duration is longer than the electron–phonon coupling time, and the main processes that occur in the particle are thermal ones, including particle melting and the associated phase transitions.

In place of a bulk SiC target, PLA may also be carried out from a target comprising a mixture of nanometer grain‐size C and Si powders. In this case, it was revealed that the synthesis of SiC NCs is more dependent on the type of laser. The TEM image of a sample prepared by a 532 nm ns pulsed laser, ablating a target containing 6 nm Si and 4 nm C NPs, exhibited two ranges of particles, including near‐spherical cubic SiC NCs with a diameter of 10–15 nm (type A) and hollow particles (type B) with both cubic silicon and hexagonal SiC phases (Figure 10f,g). In the case of femtosecond laser ablation in Figure 10h,i, in addition to type A and B particles, small spherical particles (type C) were found. However, in this case, type A particles are mainly polycrystalline cubic Si, and the type B hollow particles are mainly cubic Si and cubic silicon dioxide. The SiC phase was found in separate small particles (type C). The C 1s XPS spectrum of the ns‐laser‐treated sample exhibited that the contribution of C—C and C—Si is significant. In the case of femtosecond laser treatment, not only is the contribution of the C—Si (283.65 eV) remarkably lower, but also oxidation states such as C—O (285.75 eV) and O—C═O (288.83 eV) exist. To explain these observations pulse duration of the lasers was compared with the calculated times required for heat exchange and reaching the quasi‐stationary temperature distribution inside the particle *t*_NP_ (2.84 × 10^−13^ s) and in the surrounding medium *t*_T_ (2.80 × 10^−10^ s). These values are much shorter than the ns pulse duration (10^−9^ s), therefore, the main process in nanosecond laser treatment is the thermal heating of the suspended particles, and the quasi‐stationary conditions for heat exchange between a particle and a surrounding liquid are applicable. However, the duration of femtosecond pulses is shorter, therefore, absorption of a femtosecond laser pulse creates many hot electrons which may excite the nanoparticle‐ethanol interface system and induce chemical reactions at the interface of Si and C NPs on a timescale up to several hundreds of femtoseconds, giving rise to the formation of silicon oxides.^[^ ^150^ ^]^ Similar to above, laser ablating of a silicon wafer in ethanol can also result in the formation of a mixture of β‐SiC and Si NCs. As these processes fail to synthesize pure SiC NCs, further modification is essential. One of the effective ways to remove unwanted particles, especially Si NPs, is an etching process via an aqueous suspension containing HF (4 wt%) and H_2_O_2_ (6 wt%). SAED results confirmed that despite Si and β‐SiC coexistence in unmodified samples, only a cubic β‐SiC phase remained in the etched powders, indicating the complete removal of Si after the etching process.^[^ ^150^ ^]^

**Figure 11:**
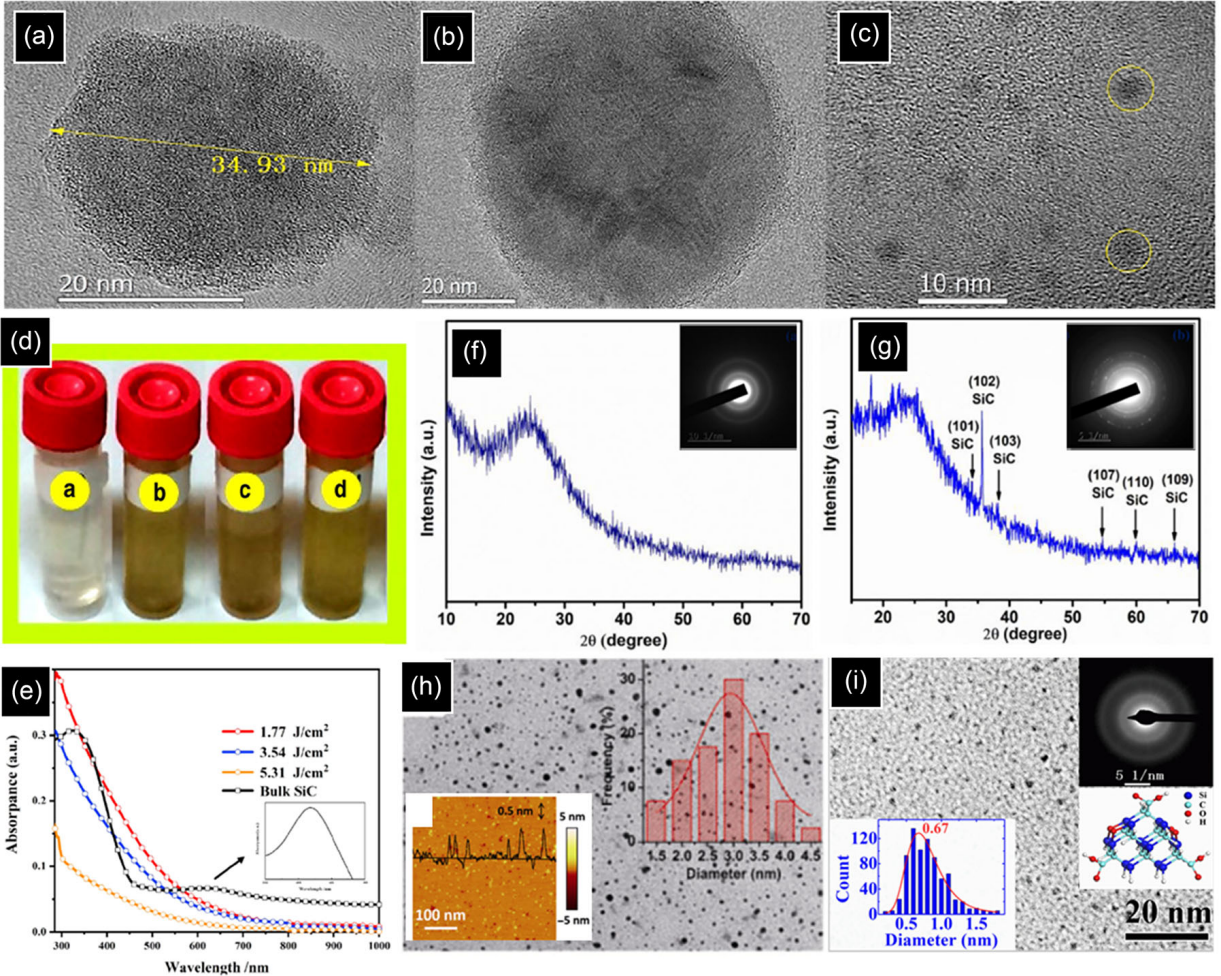
TEM images of 4H‐SiC NCs prepared under various laser fluxes of a) 5.31, b) 3.54 c) and 1.77 J cm^−2^. Reproduced with permission.^[^ ^42^ ^]^ Copyright 2022, Elsevier. d) 4H‐SiC NCs colloidal suspensions prepared at different laser fluences (increase of fluence from left to right). Reproduced with permission.^[^ ^151^ ^]^ Copyright 2018, Springer Nature. e) UV–vis spectra of SiC bulk and NCs prepared by various laser fluences. Reproduced with permission.^[^ ^42^ ^]^ Copyright 2022, Elsevier. XRD pattern of 6H‐SiC NPs produced in f) water and g) ethanol with their corresponding SAED patterns. Reproduced with permission.^[^ ^42^ ^]^ Copyright 2022, Elsevier. h) TEM and AFM images of 2D SiC QDs. Reproduced with permission.^[^ ^158^ ^]^ Copyright 2018, Springer Nature. i) TEM image and SAED pattern of 3C‐SiC NCs formed by the combination of etching and hydrothermal processes. Reproduced with permission.^[^ ^168^ ^]^ Copyright 2016, AIP Publishing.

Apart from the type of laser, the power of the laser remarkably influences the final characteristics of the NCs. As is seen in **Figure** **11**a–c, the high value of femtosecond laser flux (5.31 J cm^−2^) formed single and polycrystalline NCs with uneven and large particles above 20 nm.^[^ ^42^ ^]^ However, decreasing the power to values around 1.77 J cm^−2^ was effective in producing only spherical single‐crystal NCs with the smallest size distribution (1.5–4.5 nm). This trend was also seen in another survey as SiC NPs prepared by nanosecond laser ablation of the silicon target in ethanol. While the laser flux of 1.5 J cm^−2^ created spherical SiC NCs with a size distribution of 5–15 nm, the laser flux of 5 J cm^−2^ formed pillar particles with an average size of 55 nm which underwent agglomerations. In addition, by increasing the laser flux, the suspension color of SiC NPs gradually changed from light brown yellow to dark brown yellow, showing the higher concentration of suspended NPs in ethanol Figure 11d. The laser with a higher flux value penetrates deeper inside the Si target and produces higher concentrations of ablated ions and molecules, forming larger NCs with higher concentrations.^[^ ^149^ ^]^ The change in the size of NCs is directly linked to the optical properties of NCs. Based on the UV–vis curves of the formed SiC NCs by different laser powers in Figure 11e, the lower size of QDs due to the lower power of the laser, a higher surface plasmon resonance, increasing the light absorption characteristics (especially in the UV region).^[^ ^42^ ^]^ This QD size reduction increases bandgap energy and increases the QCE or the probability of radiative recombination.^[^ ^151^ ^]^

The solvent of laser ablation also impacts the size and crystallinity of SiC NCs, as the rearrangement of vaporized atoms and molecules is controlled by the surrounding medium. It was observed that by laser ablating the SiC substrate in ethanol, 2 nm fluorescent SiC QDs can be formed, while laser ablation in water produces non‐fluorescent large particles of amorphous SiC.^[^ ^152^ ^]^ This observation can be supported by another study, as the XRD pattern of NCs formed by laser irradiation of micron‐sized SiC powders in ethanol showed the diffraction peaks of 6H‐SiC, while the NPs in water failed to show any peak related to crystalline SiC (Figure 11f,g). These results were also verified by SAED patterns as NCs in ethanol possess bright spots along the ring; however, there are no bright spots for the NPs in the water medium.^[^ ^32^ ^]^ NPs formed in water underwent severe oxidation as a result of the reaction with the generated hydroxyl radicals from water molecule dissociation. However, in the case of ethanol, reducing intermediates such as C* and H* formed in the plasma protected the NPs from oxidation (Figure 10c). In another survey, β‐SiC QDs were formed by laser ablation of N‐type crystalline silicon wafer in ethanol and ethanol/Toluene (volume ratio of 7:1). SAED results showed that although the NCs formed in both solvents had β‐SiC rings, the rings of Si in ethanol/Toluene were lower, indicating the existence of a small amount of Si. This shows the direct effect of carbon‐abundant media on the purity enhancement of SiC NPs.^[^ ^153^ ^]^

In addition to the solvent, targets and pretreatments have a great impact on the formation of amorphous SiC NPs. For instance, laser ablating a yellow polished polycrystalline 3C‐SiC instead of a dark 6H–SiC polycrystalline wafer with a rough surface is more capable of producing ultrafine amorphous SiC NPs. In terms of surface modification, it is clear that the polished and slightly yellow surface reflects much more energy from the laser and absorbs less energy, resulting in a plasma with a smaller diameter and density.^[^ ^154^ ^]^

In addition to the direct synthesis of SiC NPs, the laser ablation process can be employed as a post‐treatment to further modify the size distribution. For example, SiC NPs formed by electrical discharge in a liquid may then be laser ablated. In one study, SiC NPs formed between immersed Si and C electrodes in ethanol possessed two size distributions with average diameters of 3.7 and 11.4 nm.^[^ ^125^ ^]^ Subsequent laser ablation was found effective in not only decreasing the average particle diameter to 2.3 nm but also in narrowing the size distribution by the fragmentation of the larger particles. This was confirmed by the calculation of bandgap energy, which increased by 0.5 eV and reached 3.0–3.6 eV after modification by laser ablation.^[^ ^125^ ^]^

#### 4.3.2 Hydrothermal Synthesis Method

The hydrothermal method has been broadly used as an effective process to prepare a broad spectrum of QDs from 2D materials, including BN,^[^ ^155^, ^156^ ^]^ graphene,^[^ ^157^ ^]^ and MXene;^[^ ^28^ ^]^ however, it has only recently been investigated for the preparation of SiC QDs. As with the laser ablation process, hydrothermal synthesis can be considered either a top‐down or bottom‐up method. Compared to an etching process, which uses hazardous HF, and laser ablation or plasma‐assisted methods, which are more complex, this process benefits from a safe aqueous solvent and simple equipment. A typical hydrothermal setup includes a stainless‐steel autoclave with a Teflon liner, solvent, target powders, and pH controllers. In the case of top‐down synthesis, powders are used as the initial precursors. To prepare the QDs, the autoclave is placed in an oven and, by taking advantage of high temperature (up to 250 °C) and pressure, large precursor SiC particles are broken down and gradually become smaller. It is evident that the efficiency, surface chemistry, functional groups, and dimensions of the obtained NCs are controlled by the effects of many parameters including temperature, filling factor (suspension/reactor volume ratio), processing duration, concentration, and solvent.

By making use of the hydrothermal method, 2D SiC QDs were prepared through the hydrothermal treatment of micron‐sized SiC particles in aqueous ammonia (pH = 9). By increasing the hydrothermal time from 8 h, which is sufficient for the formation of SiC nanosheets, to 12 h, ultrathin SiC QDs with a lateral particle size of 3 nm and a thickness of 0.5–0.8 nm were formed (Figure 11h). An XRD pattern showed that the hydrothermal process had no negative effect on SiC phase transformation as the SiC NCs preserved the initial 6H phase, demonstrating the top‐down process.^[^ ^158^ ^]^ The 2D‐SiC particles were found to be stable in aqueous solution, and the authors demonstrated an easy surface modification through BSA decoration. Although the absorption peak of pure SiC NCs with pure BSA before the hydrothermal process was located at 300 nm, the SiC@BSA NPs absorption peak appeared at a redshifted value of ≈320 nm, stemming from the nanoparticle‐protein conjugation.^[^ ^159^ ^]^ However, when DNA was used, the maximum absorption peak of DNA‐SiC NCs shifted to lower wavelengths (245 nm), which revealed that DNA was successfully attached to the SiC surface.^[^ ^160^ ^]^ These molecules are mainly responsible for modifying the optical properties and enhancing the performance of biosensor applications. However, the top‐down method is less attractive as it is incapable of controlling the size of NCs, and it therefore produces low PLQY SiC NCs, which adversely influence PL performance in the optoelectronic applications.

Compared to top‐down hydrothermal synthesis, the bottom‐up hydrothermal method uses Si and C‐containing compounds, with size controllability, ease of use, and high PLQY, which would be a breakthrough in the SiC QDs area. We are not aware of reports, however, that claim that hydrothermal synthesis was successful for SiC NCs formation. There is one exception, which declares that 30–40 nm Silicon Carbon NCs were prepared from 3‐aminopropyltrimethoxysilane (APTMS). Although XPS of C 1s and Si 2p curves indicated several bonds, including C—C, C—H, C—N, C═O, Si—O—Si, and Si—O—C, no Si—C bond appeared.^[^ ^161^ ^]^ It is interesting to note that it has remained a controversial issue, as several papers have used a similar procedure and precursors; however, the products were named silicon QDs (Si QDs) or silicon‐doped carbon QDs (Si‐CQDs).^[^ ^162^, ^163^, ^164^, ^165^ ^]^ Some solvothermal methods reported the successful formation of SiC nanorods and nanowires^[^ ^81^, ^166^, ^167^ ^]^ but these nanostructures are much larger than the QDs.

The hydrothermal method is found to be effective when coupled with other synthesis processes to tune the size of NPs. For instance, a hydrothermal‐assisted etching method enabled the preparation of SiC QDs smaller than 1.5 nm with an average size of 0.67 nm. After chemical etching of microcrystalline SiC in HF/HNO_3_, followed by ultrasonication of the resultant porous SiC in water, the colloidal solution was hydrothermally treated at 180 °C for 2 h. As is seen in Figure 11i, the SAED pattern of the SiC NPs shows blurry rings, demonstrating the amorphous structure of QDs. When the size of particles is about a few angstroms, it transforms from a nanocrystal into a molecule‐like cluster. These NPs are sufficiently small so that the polytype is not a meaningful description of the structure. Moreover, these tiny NPs can benefit from a strong QCE as their diameter is significantly lower than the exciton Bohr diameter of SiC (1.4–4 nm), enhancing their optical properties.^[^ ^168^ ^]^

In terms of production yield, placing the mixture of SiC powder and HF/HNO_3_ in either a closed or open reaction chamber can lead to distinct SiC NPs. It is revealed that the closed system had SiC QDs with not only higher yield production (about 1–1.5 mg) but also lower average size (about 3 nm). This can stem from the design of the closed system as the produced gases (CO_2_ and H_2_) in the etching process are confined in the system, increasing the pressure as well as the solubility of gases in the acids. Therefore, instead of forming bubbles on the surface of QDs, which staunch the formed pores, the bubbles can diffuse away from the surface more easily and form smaller pores, which results in a lower size of QDs in the ultrasonication process.^[^ ^169^ ^]^

## 5 Optical Properties of SiC QDs

SiC was used to manufacture the first commercially available blue LEDs before it was replaced by nitride‐based III‐V semiconductors in the early 1990s. The obtained external efficiency from SiC‐based LEDs was too low due to their indirect bandgap energy. Although there is no momentum difference in direct bandgap semiconductors dominating the radiative recombination and photon emission, the VBM and CBM of indirect semiconductors appear at different points in k‐space, requiring phonon involvement for recombination and thus favoring nonradiative over radiative processes. In the past decade, SiC QDs have been introduced to overcome this challenge. In QDs, the excitons, or the bound electron–hole pairs, are confined in all three dimensions, specifically when the particle size is lower than approximately twice the Bohr radius, inducing the radiative recombination. Another mechanism of emission is surface state recombination. When the QDs are extremely small, most of the atoms are present on the surface of the QD, and due to the presence of various functional groups, the electronic structure and emission can be tuned. In this section, we aim to elucidate the underlying mechanisms of radiative recombination in SiC QDs and explore how this emission can be enhanced through various techniques and optimizations (**Table** **3**).

**Table 3:** The optical properties of SiC QDs.

QDs	*D* [nm]	Study (S or E)	Emission mechanism	Functional groups or molecules	*E*_exc_ [eV or nm]	*E*_m_ [eV or nm]	PLQY [%]	*τ* [ns]	Reference
3C‐SiC	2 & 2.5–5.5	Ea)	QCE and SS	–	260–300 (SS) 300–420 nm (QCE)	413–495 nm	–	–	[149]
3C‐SiC	1–4	E	SS	Oxidized surface group	300–350 nm	400–600 nm	–	–	[178]
3C‐SiC	1–4	S and E	SS	Carboxyl	300–500 nm	370–570 nm	0.3	4.5	[174]
3C‐SiC	1–4	S and E	SS	Hydroxyl	300–500 nm	350–560 nm	0.4	8.1	[174]
3C‐SiC	1–8	E	QCE and SS	–	324–368 nm	380–680 nm	–	1.63	[255]
4H‐SiC	1–8	E	QCE and SS	–	297–365 nm	380–620 nm	–	0.89	[255]
6H‐SiC	1–8	E	QCE and SS	–	288–365 nm	400–610 nm	–	1.66	[255]
6H‐SiC	3.2–9.7	E	QCE and SS	SiO_2_ passivation	360–440 nm	440–560 nm	0.58	6.4	[209]
3C‐SiC	≈14	E	SS	BSA terminated	320 nm	350–500 nm	–	–	[159]
3C	5–20	E	SS	Oxidation	340–440 nm	450–510 nm	–	–	[118]
3C	≈3	E	QCE	PEG terminated	365 nm	370–450 nm	7.95	2590	[158]
3C‐SiC	7–14	E	QCE	–	260–480 nm	360–520 nm	≈4–12.4	0.7–1.28	[142]
SiC	12–30	E	QCE and SS	Carboxyl	340–460 nm	380–450 nm	6.1	1.37	[126]
3C‐SiC	1.5	S	SS	H‐terminated	*E*_g_ = 5.97 eV	–	–	–	[29]
3C‐SiC	1.5	S	SS	10%Si1═O‐terminated and 90%Si‐H‐terminated	*E*_g_ = 4.03 eV	–	–	–	[29]
SiC	0.67	E	QCE and SS	Oxygen containing	4.1 eV	3.3 eV	–	–	[168]
SiC	0.67	E	QCE and SS	Oxygen containing	3.8 eV	3.0 eV	–	–	[168]
SiC	0.67	E	QCE and SS	Si—Si bond	4.8 eV	N	–	–	[168]
3C‐SiC	1.83	E	QCE and SS	HO…C═O	326 nm (SS)	438 nm (SS)	–	–	[187]
3C‐SiC	–	S and E	SS	Acid anhydride	320 nm	410 & 450 nm	–	–	[188]
SiC	1–4	E	QCE and SS	COOH‐terminated	320 nm	450 nm	–	–	[189]
SiC	1–4	E	QCE and SS	H‐terminated	–	380 nm			[189]
SiC	1–4	E	QCE and SS	OH‐terminated	–	435 nm			[189]
SiC	1–4	E	QCE and SS	Si—O‐terminated	–	410 nm			[189]
3C‐SiC, H‐SiC	3.2–9.7	E	SS	C═O	327 nm	429 nm	0.22	15 ns	–
3C‐SiC, H‐SiC	3.2–9.7	E	SS	Unknown	365 nm	517 nm	0.13	–	[179]
3C‐SiC, H‐SiC	3.2–9.7	E	SS	Si═O	503 nm	560 nm	0.16	70 ns	[179]
3C‐SiC	2D SiC QDs *D* = 5–10 nm *t* = 1 nm	E	SS	1) Extra C and O 2) Clean surface		420 nm 420 nm	10–15 60	4–5 ns	[192]
3C‐SiC	1–4	E	–	NH_2_ terminated	360 nm	450–550 nm	–	–	[190]
4H‐SiC	1–2	S	SS	OH‐, F‐, H‐ terminated	320–360 nm	–	17	–	[186]
C‐SiC	2.3	E	SS	Si—C—O	340 nm	450–550 nm	–	–	[125]
6H‐SiC	10–250	E	SS	Si vacancies (*V*_Si_)	800 nm	850–1000 nm	–	–	[195]
4H‐SiC	40–50	E	SS	Si vacancies (*V*_Si_)	720 nm	850–950 nm	–	3.3 ns	[196]

a) *D* = average size (nm), *E* = experimental, *S* = simulation, SS = surface states, QCE = quantum confinement effect, *E*_exc_ = excitation wavelength (nm), *E*_m_ = emission wavelength (nm), *τ* = carrier lifetime, *N* = not mentioned, *t* = thickness

### 5.1 Possible Mechanisms of Radiative Recombination in SiC QDs

The QCE, defined as the spatial confinement of the photogenerated electrons and holes within QDs, was first introduced as a justification for the higher PL intensity of 3C‐SiC QDs (more than 1000x) than that of SiC polycrystalline wafers.^[^ ^93^ ^]^ However, understanding the details of the origin of emissions is complex.

Excitation‐independent or excitation‐dependent PL behavior has been widely used to attribute the emission of SiC QDs to surface states or QCE.^[^ ^170^ ^]^ For smaller SiC QDs (*D* = 2 nm), the PL peak wavelength is not or only slightly shifted with increasing excitation wavelength (excitation‐independent PL behavior).^[^ ^103^ ^]^ This is because as the size of SiC NCs decreases from 8 to 2 nm, the surface/volume atom ratio increases from 16 to 65%. Therefore, the majority of the atoms are present on the surface, and surface states control the optical properties.^[^ ^149^ ^]^ For larger SiC QDs (e.g., 4H‐SiC, *D* = 3.5 nm), by increasing the excitation wavelength (280 to 460 nm), the PL peak wavelengths continuously increase.^[^ ^171^ ^]^ The appearance of emissions of QDs solutions also supports this; when excitation wavelength alters from 320 to 400 and 450 nm, the color of solutions changes from blue (450 nm) to blue‐green (510 nm) and green‐yellow (545 nm), respectively.^[^ ^153^, ^172^ ^]^ While almost all researchers believe that PL in smaller SiC QDs is mainly controlled by surface states, there is a disagreement about the origin of emission in larger QDs. Some studies attribute the excitation‐dependent PL to QCE. In this case, they believe there is a size distribution of QDs in a colloidal solution, and increasing the excitation wavelength lowers the number of excited QDs with higher bandgap energies, resulting in PL peak redshift, and the properties of bulk SiC are approached.^[^ ^173^ ^]^ In contrast, others suggest it may arise from defects, abundant surface‐related emission centers, and multiple distributions of monodisperse QDs with excitation independent PL behavior, rather than from QCE. We first begin with the studies that support QCE as the main mechanism.

**Figure 12:**
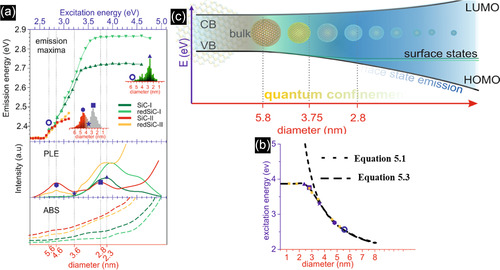
a) PL maxima, PLE, and absorption spectra of 3C‐SiC NCs with different sizes and functional groups. b) The relation between size and excitation energy. c) Schematic diagram showing the relation between bandgap and particle size. Reproduced with permission.^[^ ^174^ ^]^ Copyright 2018, American Chemical Society.

To explore the mechanism of emissions in smaller and larger SiC NCs, the PL peak maxima positions, (PLE), and absorption spectra of SiC NCs with varying sizes and different surface terminations, including carboxyl‐terminated SiC‐I (mean *D* = 2.8 nm (size distribution = 0.8−4.2 nm)) and SiC‐II (mean *D* = 4.1 nm, size distribution = 0.8−6.4 nm)), and hydroxylated‐terminated QDs designated redSiC‐I and redSiC‐II were compared.^[^ ^174^ ^]^ As is seen in **Figure** **12**a, by increasing the excitation energy from 2.3 to 2.95 eV for SiC‐II and redSiC‐II, and 2.7 to 2.95 eV for SiC‐I and redSiC‐I, the positions of the PL peak maxima experienced a blueshift along a consistent trendline. This excitation‐dependent PL behavior was related to the QCE. Above 2.95 eV to 3.3−3.5 eV, a transition between the QCE and surface state recombination was proposed as these curves begin to separate. As excitation energy increases, increasing differences develop between the peak positions for SiC‐I and redSiC‐I, demonstrating an increasing contribution of surface state recombination. Above 3.3 and 3.5 eV, only surface states control the fluorescence properties as the curves become flattened. Regarding the PLE results, the emission intensities of SiC‐I and redSiC‐I decrease above 4.5 eV excitation energy; however, the PLE intensities of SiC‐II and redSiC‐II increase as the excitation energy increases, implying that larger particles have continuous excitation spectra, similar to direct bandgap semiconductor QDs, while small particles show narrow excitation spectra, the same as fluorescent organic molecules.^[^ ^174^ ^]^ To quantitatively recognize the boundaries between various phenomena, different formulas have been established. In a simplified form, the theoretically and experimentally determined fitting Equation (13) and Equation (14), respectively, represent the relation between size and bandgap energy of 3C‐SiC NCs based on QCE.^[^ ^168^ ^]^

(13) $$E_{g}\text{(QD) = 2}\text{.03 + }\frac{4\text{.2}}{r^{2}}$$

(14) $$E_{g}\text{(QD) = 2}\text{.2 + }\frac{1\text{.95}}{d^{0.97}}$$

The effective mass approximation adequately describes the size dependence for particle sizes above 3.5 nm; however, it fails to be accurate for the smaller particles due in part to the increase in the share of surface contribution below a certain size, making the effective bandgap constant. Thus, a modified fitting formula of effective mass approximation should be used for the 3C‐SiC NCs as follows

(15) $$E_{g}\text{(QD) = 3}\text{.87 }–\text{ (2}\text{.03 + }\frac{4\text{.2}}{r^{2}}{\text{) e}}^{-{\left(\frac{\text{2×2}\text{.85}}{r}\right)}^{2}}$$

**Figure 13:**
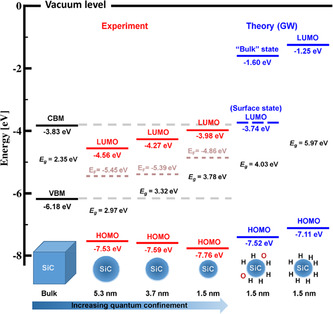
Energy band diagrams of bulk 3C‐SiC, experimental SiC NCs with various sizes, and theoretical calculations of H‐terminated and co‐H and partial Si═O terminated 3C‐SiC NC (1.5 nm). Reproduced with permission.^[^ ^29^ ^]^ Copyright 2020, American Chemical Society.

**Figure 14:**
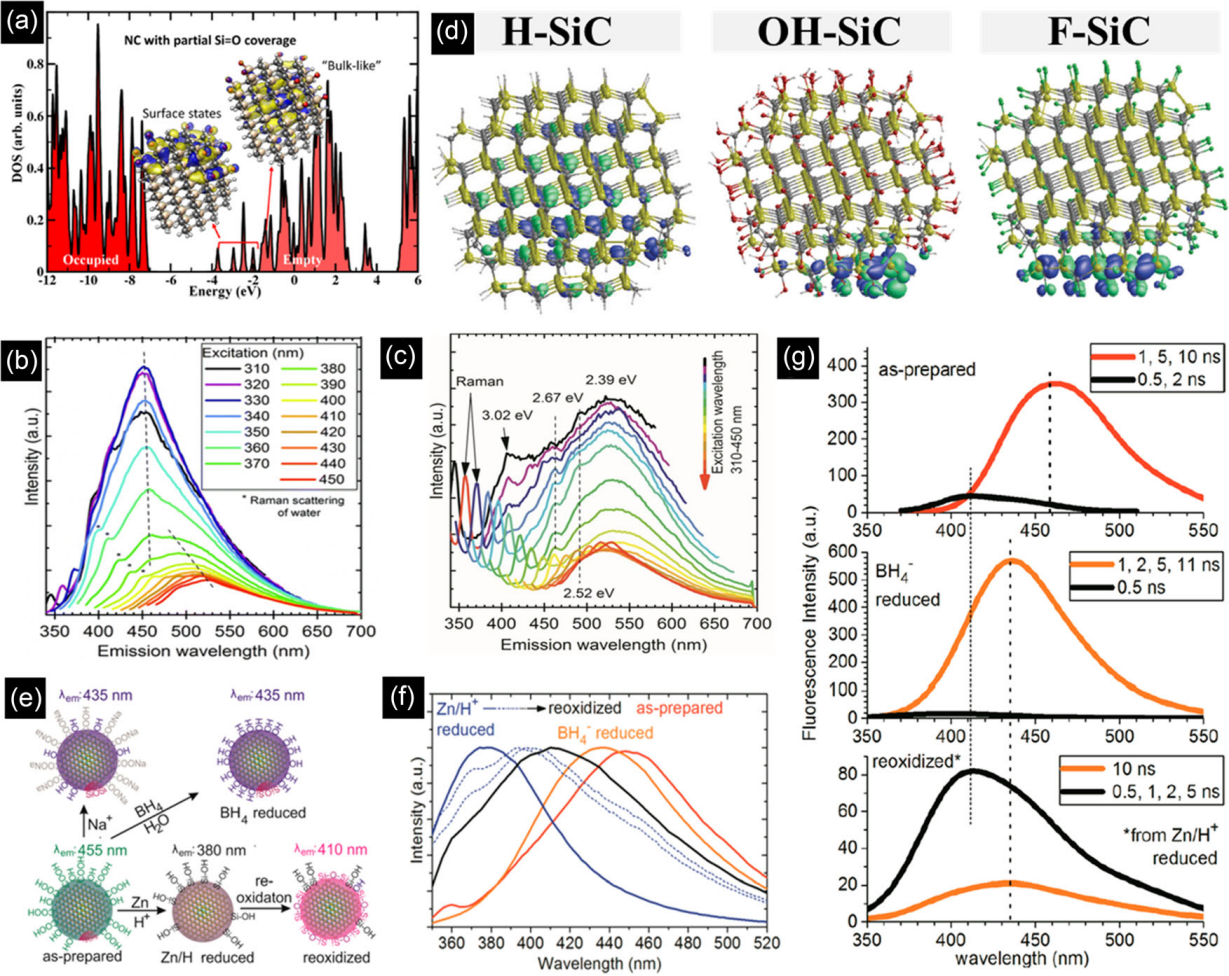
a) Calculated DFT of the 3C‐SiC NC with oxygen termination on double bond sites. Reproduced with permission.^[^ ^29^ ^]^ Copyright 2020, American Chemical Society. b) PL spectra of 3C‐SiC NCs aqueous suspension. Reproduced with permission.^[^ ^178^ ^]^ Copyright 2015, Royal Society of Chemistry. c) PL spectra of Sample II that underwent aggregation and size enlargement. Reproduced with permission.^[^ ^178^ ^]^ Copyright 2015, Royal Society of Chemistry. d) HOMOs of the 2 nm 3C‐SiC QDs terminated by H, OH, F. Reproduced with permission.^[^ ^186^ ^]^ Copyright 2016, Royal Society of Chemistry. e) Illustration diagram and f) PL spectra of different surface‐terminated SiC NCs. g) Decay‐associated spectra (DAS) of as‐prepared and reduced samples. Reproduced with permission.^[^ ^189^ ^]^ Copyright 2016, American Chemical Society.

As is seen in Figure 12b,c, researchers believe that ultrasmall NCs less than 2.85 nm show molecular behavior and surface states control the radiative recombination. They think there is a transition between the QCE and surface state recombination for the NCs between 2.8 and 3.75 nm. For larger particles less than 5.8 nm, and near to Bohr diameter of 3C‐SiC (2.0 nm), QCE and the effective mass approximation are said to be dominant and valid.^[^ ^117^ ^]^ Further extrapolation also predicts that the QCE disappears above a size of 5.8 nm.^[^ ^174^ ^]^ Different programs were also adopted to calculate the bandgap of SiC NCs, like DFT and time‐dependent DFT (TDDFT) methods.^[^ ^168^ ^]^ For NCs smaller than 1.5 nm, the DFT curve was similar to the experimental results, while for the NCs larger than 2.0 nm, the TDDFT curve matched the experimental results. This difference between the experimental and simulations was justified by the fact that SiC NCs are assumed to be hydrogen‐passivated in simulations; however, the surface of SiC NCs is full of oxygen‐containing groups in ambient conditions. This was confirmed by determining the highest occupied molecular orbital (HOMO), lowest unoccupied molecular orbital (LUMO), and Fermi level of SiC QDs relative to the vacuum energy using ultraviolet photoelectron spectroscopy, UV–vis and Tauc plot, and Kelvin probe measurements, respectively.^[^ ^29^, ^175^, ^176^, ^177^ ^]^ **Figure** **13** illustrates the experimental and theoretical (“Theory‐GW”) energy band diagrams of different SiC morphologies. Based on QCE fundamentals, the lower the size of NCs, the higher the bandgap energy, the more the HOMO energy is shifted down, the more the LUMO energy is shifted up relative to the bulk SiC band edges. Apart from the bandgap widening confirmation, on one side, all the LUMO energy levels of NCs are lower than the bulk SiC conduction band (CB), and, on the other side, the difference between the SiC bulk VB and SiC NCs HOMO energy levels is unexpectedly high.^[^ ^29^ ^]^ Based on the first‐principles calculations by means of Perdew–Burke–Ernzerhof (PBE) DFT functional + G_0_W_0_ level, the bandgap energy of H‐terminated 1.5 nm SiC NCs is 5.97 eV. In this case, the LUMO is shifted up (−1.25 eV relative to the vacuum level) compared to the bulk sample (−3.83 eV), and the HOMO level is shifted down (located at −7.11 eV) with only a small energy difference relative to the VB of a bulk sample (−6.18 eV)^[^ ^29^ ^]^ This can be confirmed by another DFT study since not only the bandgap energy was reduced from 6.9 to 5.4 eV by the enlargement of non‐terminated QDs from 0.6 to 1.2 nm, but also the HOMO and LUMO levels changed from −6.7 eV to −5.3 eV and from 0.16 eV to −0.749 eV, respectively. Further calculations for —H and O co‐terminated SiC NCs (10% oxygen passivation) revealed that Si═O bonds form surface states inside the HOMO−LUMO gap (**Figure** **14**a), shifting down the LUMO level and effectively reducing the NCs bandgap.^[^ ^29^ ^]^

As mentioned earlier, although there is a strong consensus among researchers that surface states control the excitation‐independent PL band of SiC QDs smaller than 3 nm, the origin of excitation‐dependent PL band of SiC NCs remains a topic of debate. The first group argued that this behavior corresponds to a combination of surface states and QCE in larger QDs with the size range of ≈3–3.75 nm, and pure QCE for 3.75–5.8 nm QDs. In contrast, the second group contends that QCE is not the mechanism at all and instead attributes this behavior to other fluorescent sources. Here, we explain why the arguments presented by the second group are more convincing.

**Figure 15:**
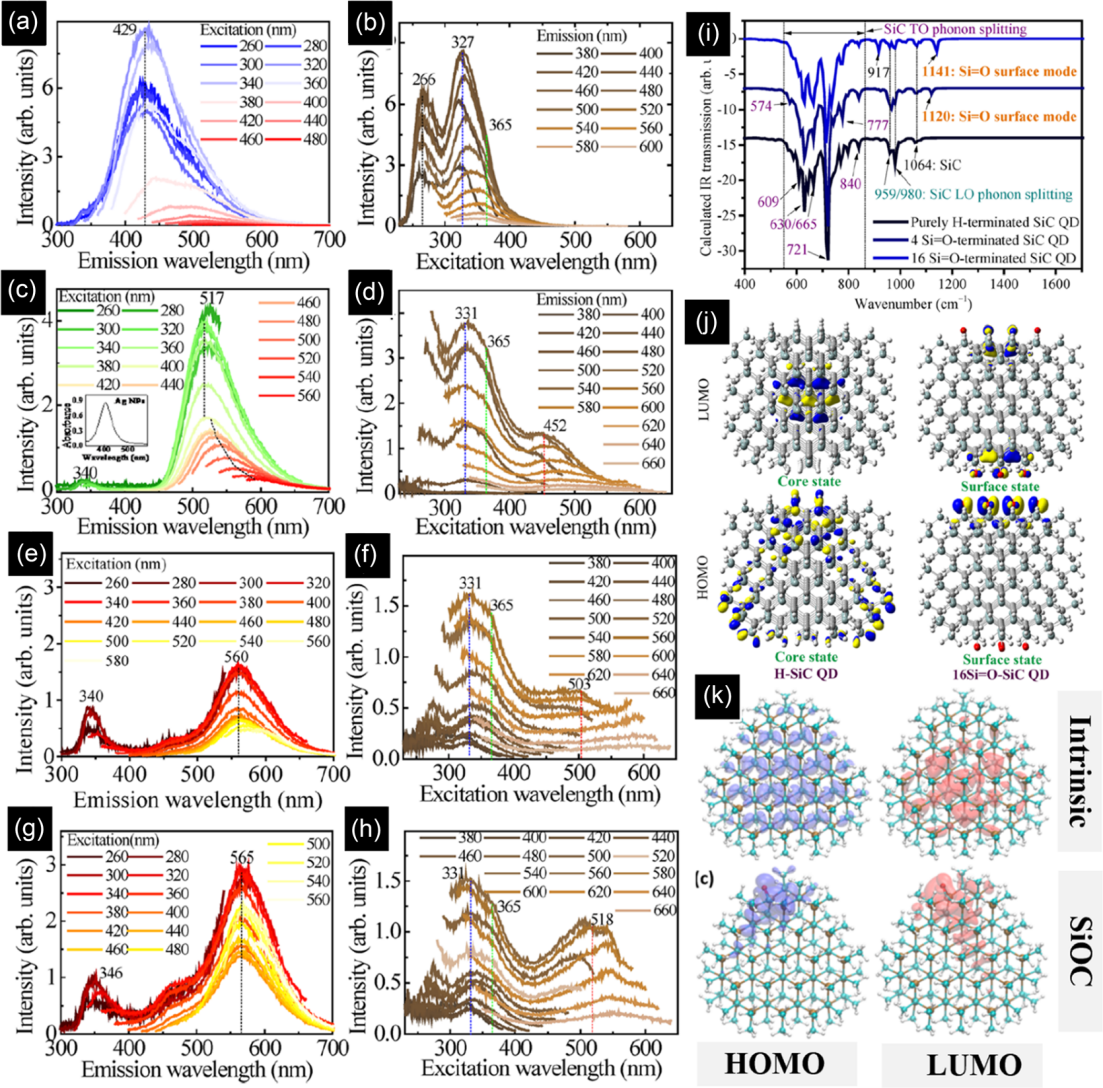
PL a,c,e,g) and PLE b,d,f,h) spectra of (a,b) the 3C‐SiC NC aqueous suspension, (c,d) 3C‐SiC NC suspension + Ag NCs, (e,f) acid‐treated SiC NCs + Ag NCs, and (g,h) aged acid‐treated 3C‐SiC NCs + Ag NCs for 15 days. i) Calculated IR transmission spectra of purely H‐terminated and Si═O‐terminated 3C‐SiC QDs. j) TDDFT [HSE06/6‐31 G(d)] calculated the HOMO and LUMO of H‐SiC QD and 16 Si=O−SiC QDs. Reproduced with permission.^[^ ^179^ ^]^ Copyright 2021, American Chemical Society. k) Orbital charge densities of the HOMO and LUMO energy levels for intrinsic and SiOC oxygen defects. Reproduced with permission.^[^ ^191^ ^]^ Copyright 2023, American Chemical Society.

First of all, the excitation‐dependent PL behavior (PL peak redshift by increasing the excitation wavelength) has been widely used as a proof for the presence of QCE in larger particles. However, this explanation often fails to accurately describe the optical properties. For instance, the redshift observed in 4 H‐SiC QDs can surpass 545 nm or 2.25 eV, which is below the bandgap of 4 H‐SiC (3.26 eV) and even lower than that of 3C‐SiC (2.4 eV). Beke et al.^[^ ^178^ ^]^ demonstrated that a mixture of two particle populations, each with narrow size distributions and excitation‐independent emissions, exhibits a redshift when the excitation energy is reduced, showing this red‐shift does not arise from excitation‐dependent sampling or QCE, but rather from the convolution of emissions from two distinct particle populations. To demonstrate this, following the synthesis of the NCs suspension (as‐prepared sample), SiC NCs with an average size of 1.5 nm (Sample I) were isolated by filtration, while larger SiC NCs ranging from 5 to 40 nm (Sample II) were obtained by washing the filtered suspension. The PL spectrum of the as‐prepared sample shows two trends: 1) no shift in the PL peak at ≈450 nm when the excitation wavelength is 310–370 nm, 2) redshift in the PL peak when the excitation wavelength range is 370–450 nm (Figure 14b). While only the excitation‐independent emission peak at 450 nm was observed in Sample I, Sample II, with a broader size distribution, exhibited excitation‐independent PL centered at 530 nm (2.39 eV), showing the origin of emission in larger particles is not QCE (Figure 14c). Moreover, the PL spectrum of sample II reveals distinct emission peaks at 408 nm (3.02 eV), 460 nm (2.67 eV), and 492 nm (2.52 eV). While the emission at 408 nm is most likely attributed to band‐edge luminescence from 6H‐SiC polytype inclusions, the peaks at 460 and 492 nm are presumed to arise from their stacking faults. This also attributes the emission to defects rather than QCE. In another study, the excitation‐independent PL band of SiC QDs (*D* = 3.2–9.7 nm) at 420 nm was quenched by introducing Ag nanoparticles into the solution, revealing a hidden excitation‐independent PL band at 517 nm.^[^ ^178^, ^179^ ^]^ Interestingly, researchers found another emission band superimposed with the low‐energy shoulder of the 517 nm peak, which appeared to redshift by reducing the excitation energy. Upon further treatment, the 517 nm band was also quenched, unveiling another excitation‐independent PL band at 560 nm (**Figure** **15**a,e,c). If we consider the first group's interpretation, the PL peak's shift to longer wavelengths with reducing excitation energy should have been corresponded to QCE, however, it is now evident that this arises from a near‐continuum of surface‐related emission states within the confined energy gap, which are selectively activated by varying excitation energy, rather than as evidence of QCE. Therefore, these observations confirm that the emission is not controlled by the QCE.

Another point is that, although semiconductors such as SiC and Si possess indirect bandgaps in their bulk form, studies have reported a possible transition from indirect to direct bandgap in Si QDs.^[^ ^180^, ^181^ ^]^ However, despite the bright fluorescence of SiC QDs, it was revealed that the bandgap energy remains indirect even in ultrasmall QDs. The relation between absorption coefficient (*α*) and excitation energy (*h*ν) for direct and indirect semiconductors is written as follows

(16) $$\alpha \;=\;B\;{\left({h\nu ‐E}_{g}\right)}^{0.5}$$

(17) $$\alpha \;=\;B\;{\left({h\nu ‐E}_{g}\right)}^{2}$$

Considering larger SiC NCs, direct and indirect values of *E*_g_ for 10 nm SiC NCs were 5.5 and 2.4 eV, respectively, similar to direct (5.3 eV) and indirect (2.3 eV) values of *E*_g_ for bulk 3C–SiC.^[^ ^170^ ^]^ The same result was also observed for smaller particles. For instance, SiC NCs with two size distributions of 3.7 and 11.4 nm (prepared by electrical discharge), and 2.3 nm (modified by laser ablation) had indirect values of *E*_g_ in the ranges of 2.5–3.5 and 3.0–3.6 eV, respectively.^[^ ^125^ ^]^ Could ultrasmall SiC QDs, with sizes ranging from 1.8 nm down to 2 Å, exhibit a direct bandgap? The answer is negative, as the indirect band dispersion curve was more like a straight line than the direct band curve, demonstrating that the SiC NCs have an indirect *E*_g_ similar to the bulk material. Therefore, while the bandgap of 6.7 Å sized SiC NCs reaches 5.1 eV, it is still indirect.^[^ ^168^ ^]^

One challenge that SiC QDs face in solid‐state applications is their tendency to aggregate, which deteriorates their optical properties. For instance, a droplet of ultrasmall SiC QDs was dried on a Si surface, and PL was measured during drying.^[^ ^100^ ^]^ In the first step of evaporation, the hydrated SiC NPs, substantially surrounded by molecules of water, are formed, showing the same PL as SiC NPs colloidal suspension. In the second step, the hydrated shell around the NPs is removed, and the initial, blue‐shifted emission peak begins to redshift, giving rise to NP aggregation and the termination of the QCE. This may be explained by the wavefunction overlap of adjacent SiC NCs, transforming the optical properties due to NC‐size quantum confinement to bulk‐like optical properties.^[^ ^178^ ^]^ Therefore, the presence of a matrix or shell around the NCs is crucial. One route to mitigate the agglomeration problem of SiC QDs is by adding polystyrene to a toluene suspension of the SiC NCs, followed by casting on a Si wafer. By this means, a transparent SiC QDs/polystyrene film with blue luminescence was prepared. Despite the partial decrease of film emission intensity, the emission wavelength remained almost constant with a slight blueshift (15 nm) due to surface oxidation.

### 5.2 Effect of Functional Groups on Surface State Recombination

Compared to various ligand‐passivated semiconductor QDs,^[^ ^182^ ^]^ SiC NCs benefit from a ligand‐free synthesis process. While surface‐bonded ligands have been capable of enhancing optical properties, their insulator characteristics significantly impede carrier transport in solid‐state electroluminescent optoelectronic devices.^[^ ^183^ ^]^ However, naked QDs undergo aggregation in the solid‐state thin film fabrication, destroying the desired quantum properties. In terms of passivation, preparing a core/shell structure was found to be effective in boosting the PL intensity of QDs.^[^ ^184^ ^]^ However, some plasma‐generated SiC QDs coated with SiO_2_ failed to show any emission probably due to the formation of numerous defects at the core/shell interface, inducing the non‐radiative recombination.

Among various methods, functionalization had great potential to tune the fluorescence properties in small SiC NCs. For instance, in one of the earliest studies, 1–3 nm SiC QDs were modelled using DFT methods.^[^ ^185^ ^]^ It is predicted that thermodynamically stable, hydrogenated SiC QDs will lead to two specific types: dots with (2 × 1)‐reconstructed Si‐terminated surfaces or (1 × 1) C‐terminated surfaces. 1) C‐terminated and H‐rich QDs have the largest gaps, and for a diameter of about 2.5 nm, they are expected to be UV emitters. 2) If the surfaces are Si‐terminated or H‐poor, the gaps of SiC QDs systematically decrease. 3) The smallest gaps correspond to Si‐terminated and Si‐rich QDs. In fact, these gaps are as small as those of reconstructed pure Si dots. Therefore, the surface‐bonded functional groups do influence optical gaps, which are highly dependent on the type of termination. The explanation for surface states influencing core states, core absorption, and core emission is through changes in the core wavefunctions. For instance, a DFT modeling survey comparing the effect of H‐, OH‐, and F‐terminations on the HOMO and LUMO positions of ultrasmall QDs (1–2 nm).^[^ ^186^ ^]^ Notwithstanding that size reduction that contributed to the blueshift of H‐terminated NCs absorption from 2.5 to 4.4 eV, no size dependency was observed for F‐ and OH‐terminated SiC NCs. Electronic density of state (DoS) calculations showed that despite the same LUMO energies (1.0–1.1 eV) for all terminations, there is a great difference between the HOMO energies of H‐, F‐, OH‐terminated NCs at −1.23, −0.76, and −0.51 eV, respectively, corresponding to the degree of wave function localization at the surface. Although OH‐termination and F‐termination HOMOs linked to surface C *p*‐orbitals and oxygen or F lone‐pairs show a degree of localization, H‐termination failed to show significant localization (Figure 14d). Therefore, while QD size does not remarkably impact the OH‐ and F‐ lone‐pair energies due to surface localization, giving rise to size‐independent behavior, H‐termination reduces the influence of surface states on PL, enabling a size‐tunable bandgap to be maintained over a wide range of emission wavelengths.^[^ ^186^ ^]^ Therefore, understanding the impact of the type of surface termination on the fluorescence properties of ultrasmall NCs is important. This surface engineering opens an effective way to engineer the band structures, boosting the performance of optoelectronics.

Regarding surface chemistry, fourier transform infrared spectroscopy (FTIR) of suspended 3C and 6H‐SiC QDs in polar solvents, prepared by the ultrasonication‐assisted etching process, showed various functional groups, including COOH, C=O, Si‐H, and Si‐OH.^[^ ^93^, ^102^ ^]^ In addition, FTIR of NPs formed by laser ablation in water detected H and OH groups, originating from water dissociation. Although it is believed that these functional groups are responsible for the excitation‐independent emission of SiC NCs, the effect of each functional group should be distinguished.

Researchers have attempted to find those surface functional groups responsible for SiC NCs’ optical properties. In one study, after the deconvolution of asymmetric PL and PLE spectra of ultrasmall NCs (0.67 nm) by multiple Gaussian functions, two symmetric PL and three symmetric PLE bands appeared.^[^ ^168^ ^]^ By taking advantage of alkali/acid treatments, it was revealed that two symmetric PLs/PLEs at 3.3/4.1 and 3.1/3.8 eV may correspond to oxygen‐related defects. Another defect with an excitation band at 4.8 eV was insensitive to the alkali/acid treatment, attributed to surface Si—Si bonds of NCs, and this contributed to absorption but not emission. In another study, it was revealed that the HO…C═O interaction can be responsible for strong light absorption/blue emission in ultrasmall 3C‐SiC QDs (1.83 nm).^[^ ^187^ ^]^ The bare SiC QDs PL spectrum showed two excitation‐independent and excitation‐dependent emission peaks at 438 and 470 nm, respectively, corresponding to surface‐state and QCE, respectively. Three PLE peaks, located at 267, 326, and 365 nm, were also observed. By adding KOH and increasing pH to 14, 326 (absorption)/438 (emission) peaks were diminished and removed, respectively, while 365 (absorption)/470 (emission) nm peaks were maintained and intensified, respectively. After introducing HCl to the above suspension, the initial results were regained, showing that while KOH reduces the surface states, HCl restores them. These 326 nm (absorption)/438 nm (emission) peaks are related to the HO…C═O [n(OH) to π*(CO)] interactions between the nonbonding electron of hydroxyl and π* orbitals of carbonyl groups (C═O (in COO)) on the surface of QDs. FTIR testing showed that by introducing KOH, the 1764 cm^−1^ peak attributed to the C═O (in COO) was diminished and resulted in a new absorption peak at 1668 cm^−1^, related to the ester potassium (COO^‐^K^+^). This changes the C═O energy level by interrupting HO…C═O interactions. When HCl makes a reaction with COO^‐^K^+^, COO and KCl are generated, followed by the recovery of the HO…C═O interaction. In another study, elevating the temperature above 370 K formed acid anhydride groups by inducing water condensation between neighboring carboxyl groups.^[^ ^188^ ^]^ As a result of this functional group transformation by increasing the temperature to 370 K, the PL peak of bare SiC NCs at 500 nm was replaced by two peaks at 410 and 450 nm. By further increasing of temperature to 450 K, only one peak in the UV region (400 nm) was observed, showing the effect of the anhydride groups on optical properties.

The effect of various oxygen‐containing groups on ultrasmall SiC NCs was also investigated. The as‐prepared 1‐ 4 nm SiC NCs were synthesized with a high concentration of carboxyl groups.^[^ ^189^ ^]^ The −OH‐terminated SiC NCs were prepared by the reduction of an as‐prepared sample in NaBH_4,_ while the preparation of −H‐terminated SiC QDs was performed by the reduction of an as‐prepared sample in HCl and Zn powder. The Si−O‐terminated SiC NCs were also produced by illuminating the H‐terminated SiC QDs with 320 nm wavelength light for 2 h, followed by storage for a few days in water. Based on PL results, there is a clear blue shift from 450 nm for as‐prepared to 435 nm and 380 nm for OH‐terminated and H‐terminated NCs, respectively (Figure 14e,f). In the aspect of H‐terminated SiC NCs, theoretical calculations of H‐terminated ultrasmall NCs (1.5 nm) indicated the bandgap energy is 5.97 eV, showing the PL results’ insensitivity to H groups. However, the emission of H‐terminated samples experiences a redshift from 380 to 410 nm during the reoxidation process. Decay‐associated spectroscopy (DAS) was used to distinguish a defect‐related recombination center from the main emission center, being overlapped in the PL spectrum. According to DAS results in Figure 14g, two emission centers with low and high intensities were found in all samples. Regarding the lower one, the intensity and lifetime become weaker and shorter by increasing the reduction degree of NCs, thereby removing carbon−oxygen groups. This peak is related to Si—O bonds, as the peak is intensified after storage in water. Therefore, in spite of the low contribution of Si—O bonds in the overall emission for COOH, OH, and H‐terminated NCs, they become dominant in re‐oxidized samples when fresh Si atoms on the greatly reduced surfaces are transformed to Si—O bonds during water exposure. In this case, Si—O defects form localized states with individual absorption and emission peaks.^[^ ^189^ ^]^

However, the effect of functional groups on fluorescent properties is not restricted to only the UV and blue regions. It was revealed that ultrasmall SiC QDs have green and yellow emitting centers dominated by strong blue emission (excitation/emission at 327/429 nm), corresponding to C═O groups (Figure 15a,b).^[^ ^179^ ^]^ By taking advantage of an overlap between SiC QDs’ blue emission (donor) and Ag NPs’ absorption band (acceptor), inducing the nonradiative energy transfer from the excited donor to the ground‐state acceptor, the blue emission was quenched, and a hidden peak with excitation/excitation‐independent emission at 365/517 nm appeared (Figure 15c,d). However, the authors failed to discover the surface state corresponding to this greenish emission. Then, the surface of QDs was treated with HF, followed by mixing with Ag NPs to lessen the effect of the blue and green emissions. As is seen in Figure 15e,f, another peak with excitation/excitation‐independent yellow emission appears at 503/560 nm. This emitting center has a lower photon energy (2.39 eV) than the corresponding 6 nm QD *E*_g_ (2.54 eV), indicating this peak is related to the surface states. As the intensity of yellow emission doubled after storage for 15 days Figure 15g,h, this band should correspond to oxygen‐related surface states. In order to further identify the origin of this surface state, phonon modes for QDs terminated with different functional groups, including C═O, C—OH, Si—OH, C—O—C, or Si—O—Si, and Si═O, were modeled by the TDDFT method. By comparing with the calculated IR transmission results, the conclusion was that the Si═O bond is involved. This was verified by the observation of 1120–1141 cm^−1^ phonon mode in 4 Si═O‐terminated (C_168_Si_216_H_240_O_4_) and 16 Si═O‐terminated SiC QDs (C_168_Si_216_H_216_O_16_), increasing as the oxidation degree of the QD surface increases (Figure 15i).^[^ ^179^ ^]^ Although an H‐SiC QD suffers from delocalized HOMO and LUMO orbitals, mainly distributed in the QD core, by increasing the oxidation degree, both HOMO and LUMO orbitals become highly localized around the Si═O bonds, controlling the emission (Figure 15j). Based on the PLQY results, the blue emission has the highest PLQY of 0.22% while the PLQY for green and yellow emissions were recorded at 0.13 and 0.16%. However, according to the time‐resolved photoluminescence (TRPL) results, the yellow emission had a longer lifetime (more than 70 ns) than blue‐green fluorescence (<15 ns).^[^ ^179^ ^]^

In another study, amine‐terminated cubic SiC NPs (SiC‐NH_2_) with partial oxygen‐containing groups were synthesized by using the Hofmann degradation process.^[^ ^190^ ^]^ Briefly, the amide is formed after the NPs are chlorinated and treated with ammonia. The sodium hypobromite in the next step transforms the primary amide into an intermediate isocyanate, followed by primary amine hydrolysis. The chlorination step in the process substitutes —OH groups for —Cl groups, and Cl groups are exchanged to —NH_2_ groups in the presence of ammonia. Compared to functionalized SiC QDs with oxygen‐containing groups, amine‐terminated SiC QDs had significantly lower emission fluorescence intensity. However, after conjugation of BSA to NH_2_‐terminated SiC QDs, the emission remarkably reappears as the amino groups are transformed to amide and hydrogen bonds are broken.^[^ ^190^ ^]^ This surface modification suggests the use of SiC QDs for the next generation of biosensors as well as for drug delivery applications.

Another emitting source producing a blue color is Si—C—O. This group is formed by laser ablation of SiC NCs as a modification process. The as‐prepared SiC NCs by electrical discharge in liquid with two size ranges of 3.7 and 11.4 nm showed an emission peak at 410 nm, attributed to the bandgap transitions. Compared to this, laser irradiation of SiC NCs (2.3 nm) not only increased the intensity of the emission peak but also broadened the emission band and formed a shoulder at higher wavelengths around 450–550 nm. The lower size distribution of laser‐ablated NPs should have narrowed the emission peak; therefore, this shoulder was attributed to the intensified surface oxidized groups such as Si—C—O, Si—O—Si, and C—O—C observed in the FTIR results.^[^ ^125^ ^]^ In another survey, the comparison between the ns and femtosecond lasers revealed that surface Si—C—O bonds are responsible for the emission. These bonds may form additional levels near the VB or CB, influencing the bandgap energy.^[^ ^125^ ^]^

The effect of various oxygen impurities, including SiOCH_3_, SiOC, COH, SiOSi, SiOH, and intrinsic (no defect), was investigated by using first‐principles modeling and density matrix dissipative dynamics, governed by the Redfield equation of motion. The Redfield equation describes the time evolution of the reduced density matrix of a strongly coupled quantum system that is weakly coupled to an environment. The calculated DOS results were the same for all samples, except for SiOC, and failed to show any localization in oxygen impurities, indicating almost identical bandgap energy (Figure 15k). However, in the case of SiOC, a new HOMO with small degeneracy was formed and the bandgap energy significantly decreased. In addition, PLQYs above 40% are predicted for LUMO to HOMO transitions for intrinsic SiC NCs as well as those containing SiOCH_3_ and SiOH, while the PLQY for SiOC is about 7%. Although the presence of oxygen in the synthesis environment is inevitable, it is predicted that in favorable configurations, high PL can be realized in both intrinsic and oxygen‐containing SiC NCs.^[^ ^191^ ^]^

**Figure 16:**
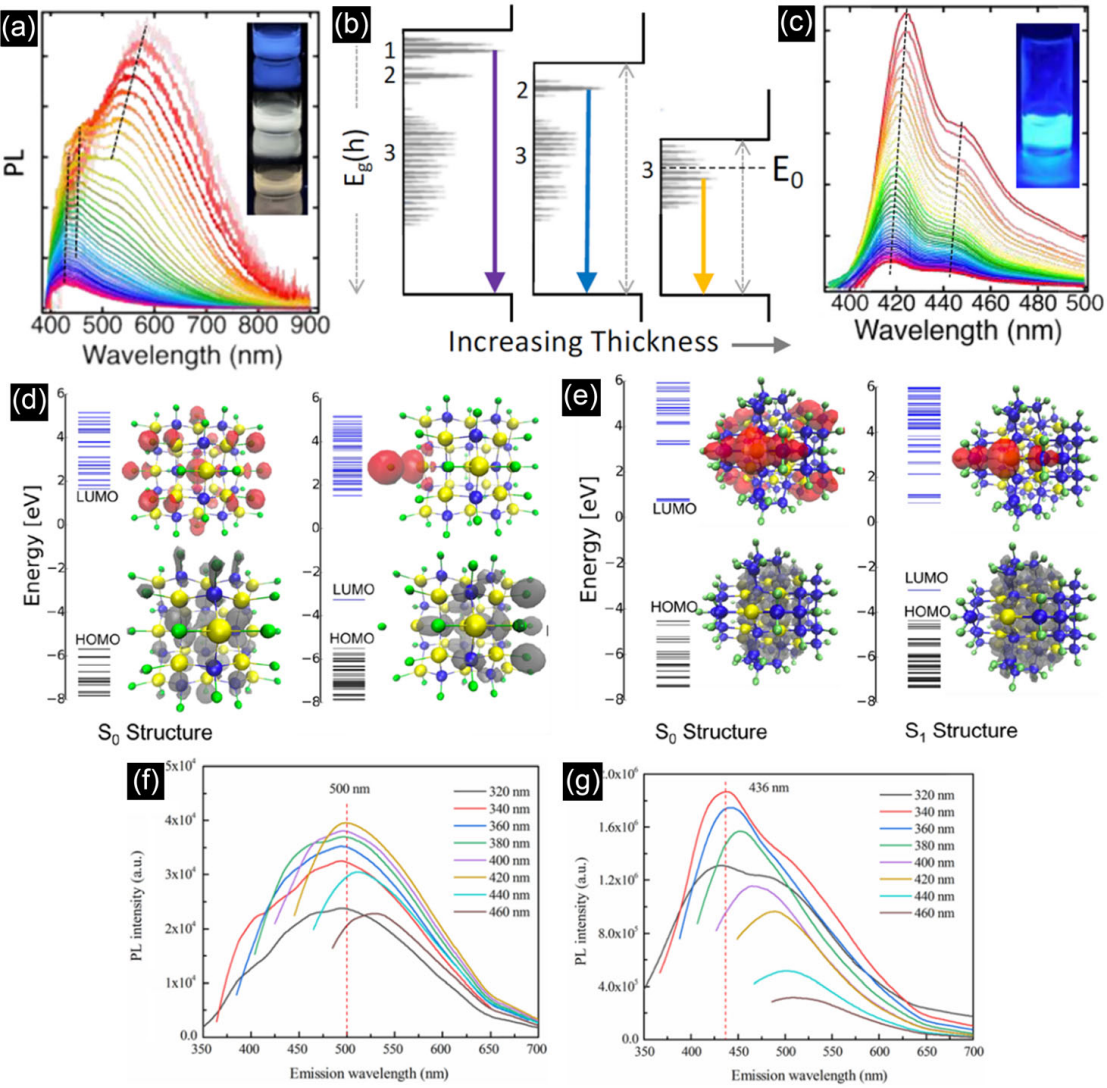
a) The effect of 2D SiC QDs concentration (dilution with centrifugation) on the PL spectra of oxygen‐unshielded 2D SiC QDs with a b) schematic of surface impurity emission modulated by over confinement. c) The effect of 2D SiC QDs concentration (dilution with centrifugation) on the PL spectra of plasma‐treated, surface‐passivated 2D SiC QDs. Reproduced with permission.^[^ ^192^ ^]^ Copyright 2024, American Chemical Society. Electronic energy levels and charge density distributions of HOMOs and LUMOs for S_0_ and S_1_ structures of d) pristine SiC QDs with radius of 0.5 nm and e) carbon‐coated SiC QDs with radius of 0.5 and 0.1 nm thickness (C, Si, and H atoms are blue, yellow, and green). Reproduced with permission.^[^ ^202^ ^]^ Copyright 2017, American Chemical Society. PL spectra of NCs formed by nonthermal plasma f) without and g) with thermal treatment at 1200 °C. Reproduced with permission.^[^ ^113^ ^]^ Copyright 2022, Elsevier.

Salim A. Thomas et al. made a significant breakthrough in SiC research area by synthesizing 2D layered SiC QDs (5–10 nm in diameter, 1–6 atomic layers thick) using a nonthermal plasma method, followed by tip sonication.^[^ ^192^ ^]^ To achieve this, a narrow reactor with high precursors flow rate was employed. Taking advantage of plasma‐annealing process after synthesis and sonication in nonpolar solvents in a very controllable oxygen‐shielded environments, 2D SiC QDs (with four atomic layers) with a narrow blue emission and highest PLQY of ≈60% were achieved. It is interesting to note that in their previous work, the unpassivated spherical SiC NCs were nonluminescent. Compared to oxygen‐shielded, plasma‐annealed 2D SiC QDs, unshielded ones exhibited broad blue/green/white PL with 10−15% QY. To investigate the PL origin, the QDs were grafted with 1‐dodecene and separated via ultracentrifugation. Based on **Figure** **16**a, showing the effect of colloidal concentration (prepared by ultracentrifugation) on PL spectrum of unshielded NCs, 2D SiC QDs show size dependent impurity/surface emission, related to excess surface carbon and oxygen formed during synthesis and processing. Based on these and FTIR results, three fluorescent channels at 420, 455, and 550 nm were detected, corresponded to SiCH_3_ and SiOCH_3_ (were predicted by first‐principle modeling to show high PLQYs above 40%; please refer to the previous paragraph), SiOCH_2_CH_3_ and SiOCH_2_CH_2_CH_3_, and higher order complex O and C bonds, respectively. They believe compared to spherical ≈2 nm SiC QDs does not show QCE (NC size is close to Bohr diameter), the thickness of 2D SiC QDs (≈1 nm) is well below the Bohr diameter, enhancing the probability of lateral confinement. Therefore, thicker 2D SiC QDs have lower bandgap energy, eliminating the high energy impurity‐emission states, shifting the PL peak to higher wavelengths (Figure 16b). In contrast, oxygen‐shielded NCs showed bright, narrow blue PL with minimal size dependence, pointing to a highly optimized impurity/defect‐driven PL mechanism in clean, strongly confined SiC. Oxygen shielding reduces oxygen incorporation, and plasma annealing removes excess carbon, forming a balanced Si:C stoichiometry on the outer surfaces (Figure 16c).

Due to its inherent large bandgap, SiC QDs inherently emit light in the green‐blue‐UV spectrum without any modifications. While surface state emissions can flatten bands and reduce the bandgap, contrary to some theoretical predictions, modifications to the surface have not successfully shifted the PL wavelength into the red‐infrared spectrum, a shift that would be advantageous for various applications, particularly in the life sciences. To address this shortfall, researchers have concentrated on doping and creating color centers in SiC NPs. Color centers have attracted much attention in a variety of applications, from single photon emitters or spin qubits to in vivo deep‐tissue imaging and drug delivery.^[^ ^193^, ^194^ ^]^ To achieve this, mm‐size 6H‐SiC crystals are exposed to neutron irradiation for 5 h to produce Si vacancies (*V*_Si_) of concentration on the order of 10^15^ cm^−3^, followed by the synthesis of SiC NCs (10–250 nm) via a mechanochemical process.^[^ ^195^ ^]^ However, this process decreased the concentration of Si vacancies to 10% of the initial concentration. Regarding the temperature‐dependent PL, although there is a broad emission band from 850 nm to above 1000 nm at 300 K, three distinct emission peaks related to V_1_ (865 nm), V_2_ (887 nm), and V_3_ (906 nm) zero phonon lines of 6H‐SiC *V*_Si_, were observed at 40 K in particles larger than 300 nm. Apart from neutron irradiation, by taking advantage of 2 MeV electron irradiation of n‐type 4H‐SiC in water and ultra‐short pulsed laser ablation, Castelletto et al.^[^ ^196^ ^]^ were successful in preparing SiC NPs 40–50 nm in diameter, emitting near‐IR light in the range of 850–950 nm. This emission stems from the silicon‐vacancy color centers. Notably, the color centers are preserved after PLA of NPs with a PL lifetime of 3.3 ns, and the SiC NPs colloidal suspension remained stable for several months. Beke et al.^[^ ^197^ ^]^ induced divacancies in SiC nanoparticles ranging from 4 to 6 nm by synthesizing SiC powder enriched with defects and subsequently annealing the NPs produced via wet chemical etching. Although they successfully demonstrated an optically detected magnetic resonance (ODMR) signal from drop‐cast nanoparticles, the luminescence intensity remained relatively low. Nevertheless, the successful production of SiC NPs with sizes of 10 nm or smaller, featuring color centers, holds significant promise. Drawing parallels with the success of nanodiamonds containing nitrogen‐vacancy (NV) centers, this development could pave the way for the realization of robust, quantum bit‐associated optical properties in SiC NPs.

### 5.3 Effect of Synthesis Parameters on Optical Properties

A number of synthesis parameters may be varied to tune the optical properties of SiC NCs. In this section, the impact of different factors on the emission wavelength, intensity, and PLQY is discussed.

#### 5.3.1 Ultrasonication

Among all synthesis methods, the ultrasonication‐assisted chemical etching process recorded the highest PLQY for SiC NCs at 17%.^[^ ^198^ ^]^ However, it has a serious drawback. It does not matter what type of SiC polytype is used; the ultrasonication process induces phase transformation, affecting the optical properties. In one of the studies, it was observed that the SiC QDs (1–8 nm) synthesized from different 3C, 4H, and 6H SiC sources exhibited the same trend and luminescence origins.^[^ ^199^ ^]^ The PL peak wavelengths remained nearly constant at around 452 nm when the excitation wavelength was lower than 320 nm (defect‐assisted recombination). By increasing the excitation wavelength to 400 nm, the emission wavelengths increased (a combination of defect‐assisted and QCE). Further increase in excitation wavelength led to the emission wavelength increase and became more remarkable (QCE). However, when the excitation wavelength reached about 540 nm, the PL wavelengths of the three curves approached the bulk 3C‐SiC bandgap energy (2.2 eV), indicating a phase transformation from 4H or 6H to 3C. Ultrasonication is capable of providing the system with enough driving force to overcome the polytypic transformation energy barrier, especially in QDs. Therefore, as the cubic structure has the most stable phase, 6H and 4H can be converted to 3C under ultrasonication waves.^[^ ^199^ ^]^

#### 5.3.2 Solvent

Another important parameter is the ultrasonication solvent. To assess this, 3.8 nm SiC NCs were prepared by ultrasonication in different solvents (pH = 7) after the etching process.^[^ ^200^ ^]^ By increasing the excitation wavelength from 300 to 340 nm, where surface state recombination was dominant, the PL emission position of DI water suspension remained almost unchanged at 433 nm, however, the PL emission peaks of methanol, ethanol, and chloroform suspensions redshifted from 414, 412, and 410 to 434 nm, respectively. This was rationalized by referring to the chemical formula of the solvents. Compared to methanol, ethanol, and chloroform, water can be dissociated into hydrogen and hydroxyl groups, functionalizing the surface atoms more effectively. This can also be related to the dielectric constant of different solvents. Comparing the dielectric constant of dichloromethane (*ε*_static_ = 9.1), ethanol (*ε*_static_ = 24), and water (*ε*_static_ = 80), the lower dielectric constant polar solvents, the higher number of energy states form inside the bandgap, red‐shifting the PL wavelengths as well as increasing the FWHM.^[^ ^93^ ^]^

It was observed that the absorption spectrum of SiC QDs can be affected by using different solvents as the medium of ultrasonication. For example, the suspension in n‐heptane comprising ultrasmall SiC NCs (1.4 nm) with discrete and sharp UV absorption peaks at 5.66, 5.71, 4.79, 4.92, and 4.55 eV shows a dramatic energy‐level transformation from bulk to molecular‐like behavior.^[^ ^201^ ^]^ In contrast to this, 4 nm SiC NCs in water had a continuous absorption pattern with a rising trend and higher intensity, maintaining the indirect bandgap energy. The oxygen‐passivated SiC NCs prefer to stay highly dispersed in polar aqueous solvent, while they undergo agglomeration in nonpolar n‐heptane solvent, therefore, n‐heptane is capable of stabilizing only the smallest SiC NCs. The SiC NCs in dichloromethane exhibited a smoother absorption spectrum with weaker absorption intensity than that of an aqueous suspension but stronger than that of an n‐heptane suspension. This is caused by the higher dispersion of SiC NCs in dichloromethane than n‐heptane due to the larger polarity of the former.^[^ ^201^ ^]^ The type of solvent also influences the TRPL properties. The smaller 3C‐SiC QDs in water with a more blue‐shifted PL peak (surface state recombination) had a higher portion of fast PL emission decay; however, larger QDs in ethanol with a more red‐shifted PL peak (QCE) had a higher portion of slow decay.^[^ ^103^ ^]^

Although many researchers believe the solvent in the ultrasonication process changes the optical properties of the final product, a study revealed that this may not be true. To explore it, the PL tests were carried out under two different conditions, after storage and immediately after sonication.^[^ ^103^ ^]^ In the case of long‐term storage, SiC NCs in water indicate a size and excitation‐independent emission peak at around 434 nm, while SiC QDs in ethanol possessed a size or excitation‐dependent wavelength, redshifting by increasing the excitation wavelength. However, the PL and PLE results of freshly prepared SiC QDs in water and ethanol exhibited the same results. This demonstrates that the long‐term storage in different solvents differentiates the optical properties.

#### 5.3.3 pH

Another parameter that influences SiC NCs optical properties is the pH of the suspension. For instance, the emission wavelength (463 nm) of 2.7 nm 4H QDs was found to be insensitive to pH changes in the range of 2.5–7.^[^ ^171^ ^]^ However, the maximum emission intensity was obtained when the suspension pH was 3.5, followed by a substantial drop above a pH value of 4.5. The scenario is different when the pH of the SiC QDs is assessed in a wider range of pH at different excitation wavelengths. Raising the pH of 3.8 nm SiC NCs in alkaline solutions increases the emission wavelength.^[^ ^200^ ^]^ This trend was more obvious in the excitation wavelength range between 300 and 320 nm due to the surface state recombination mechanism. This redshift corresponded to abundant hydroxyl groups in the suspension with higher pH, mostly bonded to the Si terminations on the surface of NPs, decreasing the bandgap energy. This feature provides SiC NPs suspension with strong potential to be used for pH‐sensor systems.^[^ ^202^ ^]^

#### 5.3.4 Coatings

The best way to enhance the optical properties of SiC NCs is surface passivation of cores with appropriate shells. Core–shell structures can have several advantages. First, a shell acts as protection against a variety of destructive environmental factors.^[^ ^203^ ^]^ Second, it passivates surface dangling bonds of the core that may form non‐radiative recombination sites.^[^ ^204^ ^]^ Third, a shell acts to mitigate bulk‐like NP behavior due to agglomeration.^[^ ^205^ ^]^ Fourth, band engineering can be applicable as the CB and VB of the shell can be tuned.^[^ ^206^ ^]^ Finally, in optoelectronic devices such as LEDs, the shell is capable of decreasing the negative effect of Auger recombination on performance.^[^ ^207^ ^]^

A carbon shell is one candidate for tuning the optical properties of SiC QDs. In one simulation study, DFT modeling explored the influence of a variety of hydrogen‐terminated carbon‐based shells surrounding SiC QDs with different configurations on bandgap energy.^[^ ^208^ ^]^ The electronic energy levels of bare, ultrasmall QDs (0.5 nm) show the distribution of LUMO level charge densities in SiC QDs with S_0_ and S_1_ structures (Figure 16d).^[^ ^208^ ^]^ In terms of the S_0_ configuration, the HOMO and LUMO charge densities are distributed inside and on the surface of QDs, respectively. Regarding the S_1_ structure, the LUMO energy level is greatly separated from the other unoccupied energy levels due to the strong localization of LUMO charge density on the stretched bond in the S_1_ structure, decreasing the bandgap energy, resulting in a large Stokes shift. In the case of carbon‐coated SiC QDs (0.5 nm) with a thickness of 0.1 nm, the HOMO charge densities also prefer to distribute over both the core and shell parts while the charge densities of the LUMO level are mainly localized in the shell parts, which are highly dependent on the carbon layer thickness (Figure 16e). It is predicted that carbon shells may cause optical properties to fundamentally deviate from the expected quantum confinement behavior of SiC QDs. The proposed explanation for this is via self‐trapped excitons that form due to strong interactions between excitons and phonons in the vicinity of the carbon shell. This results in a large Stokes shift due to the highly localized vibrational energies associated with exciton self‐trapping.^[^ ^208^ ^]^

Researchers found other coatings more effective than a carbon shell such as silica. To replace the carbon shell with silica in SiC NCs prepared by nonthermal plasma synthesis, different methods were used.^[^ ^29^, ^113^ ^]^ One of them is heat treatment at high temperatures, inducing SiO_2_ shell formation. As seen in Figure 16f, a 500 nm PL emission maximum of bare 3C‐SiC QDs is observed for an excitation wavelength of 420 nm. However, by subsequent heat treatment temperatures of 400, 800, and 1200 °C, the PL peaks shift to 465, 450, and 436 nm (Figure 16g), respectively, and the corresponding excitation wavelengths decrease to 380, 360, and 340 nm, respectively. The main reason behind this data was attributed to surface passivation. The greenish color of bare carbon‐coated SiC QDs is related to many carbon surface defects in SiC. However, heat treatment removes the carbon layer and replaces it with SiO_2_ layers, blueshifting the PL by introducing more oxygen‐related defects to SiC QDs. In another study, it was revealed that the epitaxial growth of SiO_2_ on 6H–SiC QDs (2.3–8.3 nm) by a thermal oxidization method doubled the PLQY of QDs.^[^ ^209^ ^]^ After the epitaxial growth of silica on SiC QDs, the intensities of carboxyl‐related Surface Defect II and Surface Defect III with absorption/emission peaks at 325/427 and 365/501 nm, respectively, increased. This was confirmed as the surface silica layer enhanced the radiative transition rate of the core SiC QDs, by reducing the radiative recombination lifetime from 2190 to 1534 ns, and lessened the nonradiative transition rate, by increasing the non‐radiative recombination lifetime from 4.6 to 9 ns. This originates from the reduction of the surface dangling bonds due to the epitaxial growth of silica on the SiC QD surface (lattice mismatch of the SiC (108) and SiO_2_ (022) planes was 4.6%). When the surface silica shell was removed, the dangling bonds reappeared, which weakened the luminescence intensity, confirming the positive effect of silica epitaxial growth of the silica shell on SiC QDs.^[^ ^209^ ^]^ In contrast, there is a contradictory study showing that a silica shell coating quenched the optical properties of SiC QDs. Regarding this survey, 2–4 nm SiO_2_‐passivated 3C‐SiC NCs, which should have benefited from strong QCE or surface state recombination, failed to show any PL peak. It is believed that the passivating oxide shell creates numerous negative surface charge groups as defects, increasing the nonradiative recombination and causing a negative impact on the PL results.^[^ ^120^ ^]^

In addition to shells, molecules can be used to be conjugated to SiC NCs to modify the optical properties. By using a top‐down hydrothermal process, the fluorescence intensity of SiC/BSA NCs increased almost five‐fold relative to that of the pure SiC QDs, showing the substantial impact of BSA in SiC passivation.^[^ ^159^ ^]^ In addition, 85.2% of the initial fluorescence intensity was maintained after 12 weeks, indicating good long‐term stability of SiC/BSA NPs.

#### 5.3.5 Doping

**Figure 17:**
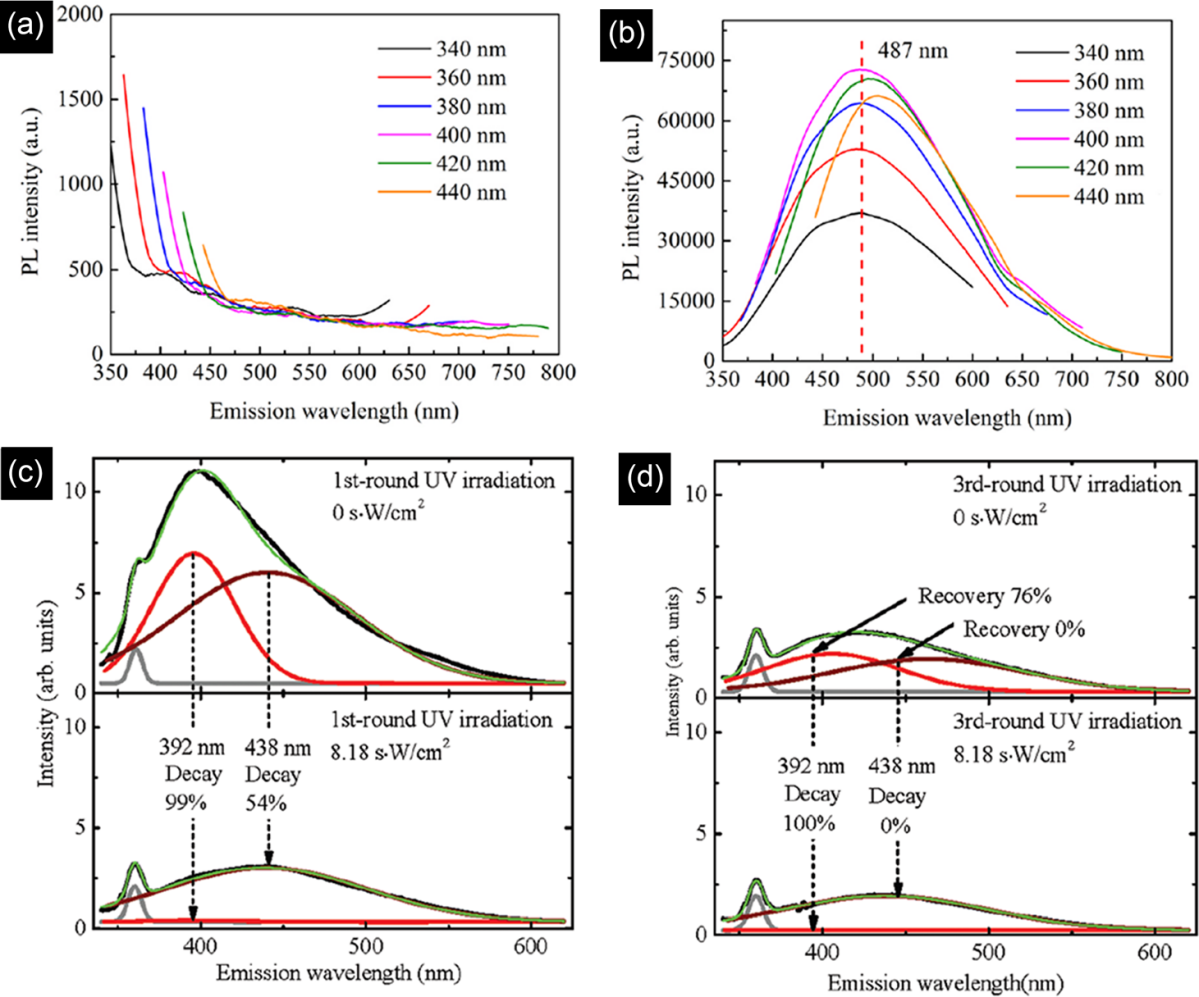
PL spectra of prepared QDs under a) Ar and b) N_2_. Reproduced with permission.^[^ ^118^ ^]^ Copyright 2023, Elsevier. PL spectra and Gaussian decompositions of 3C‐SiC QDs suspension during c) first and d) third rounds (with a time interval of 2 h) of UV irradiation (wavelength: 320 nm, dose: 8.18 s W cm^−2^). Reproduced with permission.^[^ ^211^ ^]^ Copyright 2019, John Wiley and Sons.

By using an atmospheric thermal plasma, N‐doped fluorescent SiC NPs producing blue‐green emission were synthesized. For sample preparation, Ar, Ar‐N_2_, and N_2_ atmospheres were used. As a result of the atmosphere change from Ar to N_2_, the NC size changed from 5–20 to 30‐200 nm.^[^ ^118^ ^]^ Under the excitation wavelength range of 340–440 nm, there was a slight difference between the PL peak position of SiC NCs prepared in Ar‐N_2_ (450–500 nm) and pure N_2_ (460–510 nm). Although the PL of carbon‐coated SiC NCs was extremely weak in the Ar atmosphere **Figure** **17**a, the NCs formed in N_2_ atmosphere had a remarkably stronger PL intensity than that of the Ar‐N_2_ atmosphere, showing the outstanding role of high N‐doping level in the fluorescence intensity enhancement (Figure 17b). It is suggested that the formed SiC NCs in the N_2_ atmosphere may contain fewer carbon layers, lessening the light absorption and improving PL intensity.

#### 5.3.6 UV exposure (photobleaching)

Despite PLQY enhancement by the QCE, QDs may undergo photobleaching, caused by photochemical alteration of the fluorophore, leading to the permanent loss of emission.^[^ ^210^ ^]^ In a study to assess this effect in SiC QDs, samples were exposed to UV irradiation.^[^ ^211^ ^]^ Based on the PL spectrum of bare QDs before UV exposure, there were two emitting centers, including carboxyl (COOH)‐ and ester (COOR)‐surface defects with absorption/emission peaks at 310/392 and 325/438 nm, respectively. After exposure, although the PL intensity of the 392 nm band, highly sensitive to ions and light, was remarkably reduced, this decrease was not considerable for the 438 nm band, insensitive to ions and light. Moreover, the COOH‐related surface defects were photobleached by 100% after three rounds of 20 min of UV irradiation, and 61%–72% of photobleaching was reversible (Figure 17c,d). Regarding the COOR‐related surface defects, the first, second, and third rounds of UV irradiation gave rise to their degradation by 54%, 21%, and 0%, respectively, undergoing almost 100% irreversible photobleaching. The reason behind this phenomenon is that UV light contributes to COOH ionization into COO^−^ and H^+^; however, after terminating UV irradiation, the ions can react and reform COOH, reviving a portion of fluorescence. This phenomenon can become more obvious by the introduction of K^+^ into colloidal suspension, paving the way for the ionization of COOH by UV irradiation.^[^ ^211^ ^]^

#### 5.3.7 Laser Pulse Duration and Flux

In addition to the effect of laser pulse duration on the physical properties of QDs, it can also influence the chemical and optical properties of SiC NPs. Compared to femtosecond lasers, a nanosecond laser irradiation system was found to be more effective in preparing blue‐white emitting SiC NCs, from a mixture of Si and C NCs, with higher luminescence and PLQY of about 3%, in the spectral region of 350–600 nm.^[^ ^150^ ^]^ According to XPS of Si 2*p*, ns irradiation formed smaller cubic SiC NCs with higher portions of Si—C and Si—C—O components, which appeared at 101.32 and 101.86 eV, respectively. However, NPs synthesized by the femtosecond laser had Si or SiO_2_‐based phases, confirmed by the presence of Si—Si (99.96 eV) and Si—O bonds (102.27 eV). Among all transitions that may occur, the most probable reason behind the emission corresponded to the Si—C—O groups. Therefore, the higher concentration of Si—C—O bonds in the nanosecond laser confirms the higher luminescence and PLQY. Laser flux can also change the optical properties of SiC NCs. While the emission peak of SiC NPs synthesized by a laser flux of 1.5 J cm^−2^ was centered at 334 nm, increasing the laser fluence shifted the emission peak to longer wavelengths.^[^ ^147^ ^]^ This can be attributed to the QCE as the particles became larger as a result of the increase in laser fluence. Moreover, the intensity of PL spectra increased with increasing laser fluence due to an increasing concentration of SiC NPs. In contrast to the above study, another study performed a survey showing that increasing the laser power from 115 to 460 mW failed to change the emission wavelength. This was attributed to the narrow size distribution of NCs and surface state recombination in ultrasmall NCs.

#### 5.3.8 Hydrothermal Time and Design

A top‐down hydrothermal synthesis was capable of achieving outstanding ultrathin SiC QDs with the longest PL lifetimes (2.59 μs), comparable to that of SiC QDs (4.3 ns), graphene QDs (7.52 ns),^[^ ^212^ ^]^ and MoS_2_ QDs (4.66 ns).^[^ ^213^ ^]^ The 2D SiC QDs showed an excitation‐independent PL wavelength at 405 nm with a high PLQY of about 8%.^[^ ^158^ ^]^ The more blue‐shifted emission wavelength of 2D SiC QDs compared to SiC nanosheets was related to higher QCE in three dimensions and edge effects. Moreover, the fluorescence intensity of 2D SiC QDs exhibited only slight quenching after a month, representing excellent stability.

The hydrothermal design also influences photoluminescence results. A closed chamber compared to an open container showed a narrower size distribution for the closed system (3 nm,1–8 nm) than the open system (6 nm, 1–15 nm), resulting in a narrower closed system emission peak with a slight emission maximum blueshift.^[^ ^169^ ^]^ Moreover, the PL intensity was about 30% stronger for the closed reaction chamber, attributed to better QCE and enhanced particle size control.

#### 5.3.9 Electron Beam Irradiation Dose and Time

SiC NCs formed by electron beam irradiation also showed both QCE and surface state recombination mechanisms in terms of fluorescent properties. For instance, based on the PL and PLE results of SiC NCs synthesized using electron beam radiation dose 50 kGy, two wavelength ranges at 325/363 nm and 398/445 nm (absorption/emission) were observed, relating to QCE and surface state (carboxyl and hydroxyl groups) recombination mechanisms, respectively.^[^ ^142^ ^]^

There are various factors capable of tuning the optical properties. In terms of radiation dose, the SiC QDs obtained from different radiation doses (50, 80, and 100 kGy) had almost the same *E*_g_ and PL properties, indicating these SiC QDs had similar size and crystal structure. However, the prepared SiC QDs by 50, 80, and 100 kGy radiation doses had different PLQYs of 8.62%, 8.38%, and 4.22%, respectively, showing that high radiation doses destroy the SiC QDs structure during growth.^[^ ^142^ ^]^

Regarding the silane coupling agent, concentration is highly important. For instance, when a low concentration of silane coupling agent is used, the absorption and emission peaks of 326 and 355 nm are removed. The lower the silane concentration, the lower the number of carboxyl and hydroxyl functional groups, removing surface state recombination centers. Moreover, the obtained SiC NCs at 0.4, 1.0, and 1.4 g silane coupling agent concentrations had PLQYs of 6.92%, 8.38%, and 12.39%, respectively. Raising the silane coupling agent concentration raises radiation products of silicon atoms and carboxyl and hydroxyl‐related groups, positively affecting crystal growth and enhancing PLQY.^[^ ^142^ ^]^

Choosing ionic liquids with different alkyl chain lengths in a microemulsion also changes the PL properties of SiC QDs. By making use of [OMim]PF_6_ instead of [BMim]PF_6_ microemulsions, the emission peak wavelength experienced a blue shift from 445 nm to about 431 nm. Although the size of SiC QDs formed from [OMim]PF_6_ is larger than that of [BMim]PF_6_, the energy gap value of SiC QDs obtained from [OMim]PF_6_ (5.10 eV) is smaller than that of [BMim]PF_6_ (5.14 eV) due to the smaller average droplet size of [BMim]PF_6_ (7.8 nm) compared to [OMim]PF_6_ (15.5 nm).^[^ ^142^ ^]^

#### 5.3.10 Dielectric Barrier Discharge (DBD) Design

In one study, NCs, formed by dielectric barrier discharge (DBD) emitted a blue color (432 nm) with an average fluorescence lifetime and PLQY of 1.37 ns and 6.1%, respectively.^[^ ^126^ ^]^ Since OH radicals played an important role in the DBD synthesis of fluorescent SiC NCs, the OH radical concentration should be optimized to achieve the best optical properties. First, the consumption of H_2_O_2_ produces hydroxyl radicals. Therefore, in the absence of H_2_O_2_, no fluorescent NCs were obtained. Using oxygen gas was also found to be efficient in increasing the fluorescent properties, due to the production of ^•^OH. In addition, as ^•^OH tends to be generated under alkaline conditions, a pH of 10 indicated the best optical properties. Increasing both the reaction time and input voltage (up to 50 V) satisfies ^•^OH production, giving rise to fluorescence intensity improvement of SiC NCs. However, when the voltage exceeds 50 V, abundant ^•^OH and H_2_O_2_ oxidants are formed, leading to severe oxidation of APTMS, which contributes to a substantial reduction in the fluorescence intensity of the final products.^[^ ^126^ ^]^ In terms of materials selection, the comparison between APTMS, [3‐(2‐aminoethyl) aminopropyl] trimethoxysilane (AEAPTMS), and 3‐[2‐(2‐aminoethylamino) ethylamino] propyl‐trimethoxysilane (AEAEAPTMS) with varying amounts of amino groups and chain lengths revealed that although all three precursors were capable of forming SiC NCs, APTMS showed the highest fluorescence intensity. This can be related to the lower chain length of APTMS, which facilitates the synthesis reactions.^[^ ^126^ ^]^ Moreover, the DBD process has greater potential than others like Fenton reactions in rapid and large‐scale production of QDs. Although a higher content of H_2_O_2_ was used in the Fenton process, not only is the fluorescence intensity of formed SiC NCs by DBD remarkably higher but also there is a significantly decreased time required for ^•^OH generation, accelerating the reactions.^[^ ^126^ ^]^

## 6 Applications

### 6.1 Biomedical Applications

#### 6.1.1 Cell Imaging

**Figure 18:**
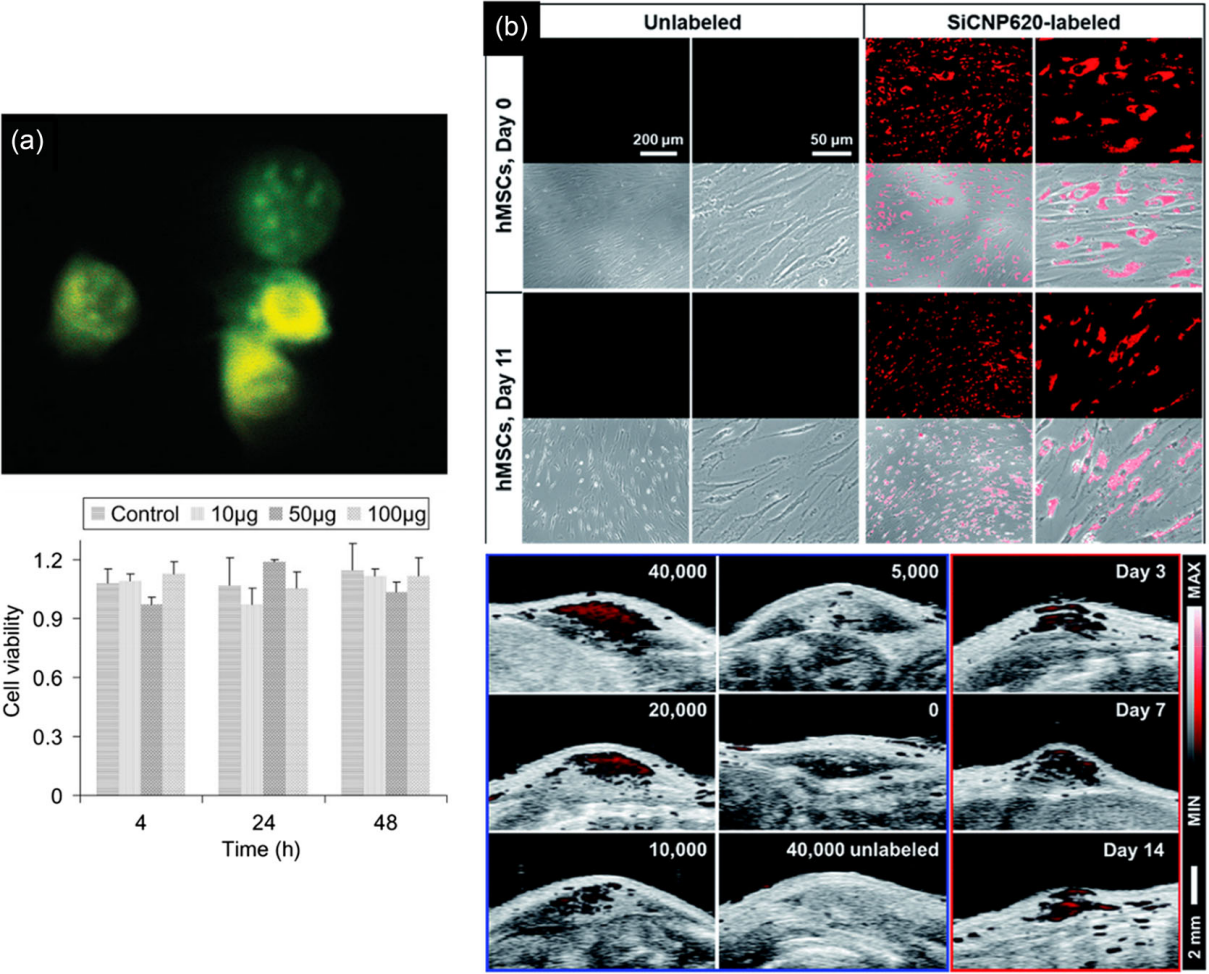
a) Top: Bright fluorescence signal from the hFOB cells cytoplasm and nuclei after 3C‐SiC nanocrystal uptake for 2 h; Bottom: Results of cell viability from MTT assay. Reproduced with permission.^[^ ^198^ ^]^ Copyright 2008, John Wiley and Sons. b) Top: Fluorescence image and overlay with bright field images of labeled and unlabeled cells after 11 days of incubation with 620 nm 3C‐SiC NPs; Bottom: in vivo tracking of SiC‐labeled mouse mesenchymal stem cells in different cell concentrations (left) and during two weeks after injection into animal subjects (right). Reproduced with permission.^[^ ^218^ ^]^ Copyright 2019, Royal Society of Chemistry.

SiC QDs have been utilized in a broad range of applications, including biomedicine, sensing, optoelectronics, and energy conversion, with particular attention drawn to their biomedical applications (**Table** **4**). Taking advantage of their small size, bright and stable fluorescence, and biocompatibility, SiC QDs have been extensively studied for biological applications, specifically in cell bioimaging and detection platforms. In one of the first attempts at using SiC QDs in biological applications, Fan et al.^[^ ^198^ ^]^ used 3C‐SiC NCs as fluorescent biological labels. The uptake of these 3.9 nm SiC NPs by human fetal osteoblast (hFOB) cells via endocytosis resulted in a bright fluorescence signal detected from everywhere within the cell membrane, including the nuclei, indicating the presence of NCs in the cell nuclei, which is consistent with the small size of the QDs (**Figure** **18**a). Regarding the outstanding stability of the SiC NCs fluorescence signal, having a photoluminescence decay exponential component of 10.9 ns, the authors were able to mitigate the effect of autofluorescence from cells. In cell autofluorescence, some molecules in cells and tissues, such as proteins containing aromatic amino acids, Nicotinamide adenine dinucleotide phosphate (NADPH), flavins, and lipopigments can naturally emit fluorescence when exposed to light.^[^ ^214^, ^215^ ^]^ Although enabling some autofluorescence‐based imaging techniques to assess valuable morphological and physiological information about cells, cell autofluorescence can interfere with the detection of signals from exogenously applied fluorophores.^[^ ^216^ ^]^ In order to assess the possible cytotoxic effects of the SiC NCs on living cells, phase contrast microscopy, as well as methyl thiazolyl tetrazolium (MTT) assay, were utilized, indicating no significant effect in the viability of human cervical carcinoma cells HeLa after being exposed to SiC concentrations up to 100 μg mL^−1^ (or 10^15^ particles) for 48 h.

In another attempt, Botsoa et al.^[^ ^99^ ^]^ incubated electrochemically etched 3C‐SiC QDs having an average size of 1.6 nm with 3T3‐L1 fibroblast cells for 15 h to evaluate the bioimaging capability of these QDs. The cellular uptake of 3C‐SiC QDs resulted in a strongly localized fluorescence signal from the area within the cell membrane as well as very strong signals from the cell nucleus, indicating a high probability of QD penetration within the nucleus membrane. This penetration can be related to electrostatic interactions between QDs and charged biomolecules within the nucleus membrane. The autofluorescence from untagged cells was almost not visible in the fluorescence microscopy images. In an alternative approach, the effect of interaction with QDs on the viability of cells was assessed by imaging the cells one week after the incubation, which indicated the absence of any cytotoxic effect in 3C‐SiC concentrations ranging from 0.1 to 2.0 g L^−1^.

In a more recent attempt to use SiC in super‐resolution microscopy, Castelletto et al.^[^ ^217^ ^]^ used 40 nm 4H‐SiC NPs in the confocal microscopy of both inside and outside of a cell microenvironment. After overnight incubation of cells with NPs at a concentration of 20 mg mL^−1^, successful cellular uptake was confirmed by observing them within the cell by comparing images from different depths across the cell dimension. Two images taken using distinct excitation wavelengths of 561 and 638 nm were superimposed in order to achieve a super‐resolution image of the 4H‐SiC NP with a resolution of 18 nm. Extending the application of SiC NPs to in vivo imaging of live cells, Chen et al.^[^ ^218^ ^]^ compared the photoluminescent and photoacoustic performance of differently sized SiC NPs to find the optimal NP size for stem cell labeling and tracking both in vitro and in vivo. After comparing 30, 80, and 620 nm SiC NPs and concluding that the 620 nm NPs have superior performance, both human and mouse mesenchymal stem cells were used for cell labeling tests. Using 3‐(4,5‐dimethylthiazol‐2‐yl)‐5‐(3‐carboxymethoxyphenyl)‐2‐(4‐sulfophenyl)‐2H‐tetrazolium (MTS) and Resazurin assays for cytotoxicity assessments showed that 620 nm SiC NPs have a negligible effect on both the viability and proliferation of mesenchymal stem cells in a wide range of NP concentrations. Labeling mesenchymal stem cells with 620 nm SiC NPs was shown to not only result in strong and stable photoluminescence signals from tagged cells, as shown in Figure 18b, but also to not affect stem cell differentiation. This subsequently made tracking the mesenchymal stem cell‐derived adipocytes and osteocytes possible. Additionally, these NPs were shown to be able to enter lipid vesicles, which can be beneficial in studying adipogenic cells. In order to verify the feasibility of using SiC NPs for in vivo cell labeling, labeled mouse mesenchymal stem cells were injected into a nude mouse, and authors were able to track the labeled cells for 14 days in vivo down to a concentration of 7800 cells per μL as shown in Figure 18b.

Having different surface functional groups as a result of different synthesis methods is shown to have an important impact on the behavior of SiC NPs in their interactions with live tissues. In one study, the effect of different SiC NPs surface functional groups, as immunomodulatory agents, on the metabolic activity of cells was investigated.^[^ ^218^ ^]^ Apart from a small change (about 0.3 nm) in the average size of NPs as a result of altering surface functional groups, the colloidal stability of NPs was significantly dependent in some cases on the NPs termination in different buffers. The interaction of these NPs with live cells is also governed by the surface functional groups due to the biomolecular corona surrounding the colloidal NPs. While “as‐prepared” NPs caused almost complete cell death, both amino‐ and hydroxyl‐terminated NPs gave rise to the metabolic activity enhancement of monocytes as well as significant changes in the cell morphology after incubation for a week. Since NH_2_‐SiC NPs lead to differentiation of monocytes to adherent macrophage‐like cells and OH‐SiC NPs result in both macrophage‐like and dendritic‐like cell morphologies, these NPs can be used as ex‐vivo stimuli for immune cell therapies.

In contrast, Beke et al.^[^ ^169^ ^]^ used 3 nm SiC NCs synthesized by an electroless wet chemical etching method in the presence of HNO_3_ as an oxidizing agent and reported no indication of QDs penetrating the cell membrane upon 48 h of incubation with HeLa cells. Authors hypothesized that this inconsistency in the cellular uptake behavior of QDs corresponded to the surface functionalization of SiC NCs, which is highly dependent on the type of precursors in the electrochemical etching process.^[^ ^99^ ^]^ Cytotoxicity assessment using HeLa cells and the alamarBlue™ Cell Viability Assay Kit also showed that these QDs have negligible effects on cell growth in concentrations below 100 μg mL^−1^. Furthermore, by observing a red shift in the emission spectra of QDs by moving the excitation wavelength toward 400 nm, the possibility of using a two‐photon excitation microscopy method for live tissue imaging was studied. Considering the ability of red or near‐infrared short pulses for deep penetration in organic tissue, using two‐photon excitation microscopy enabled deep tissue imaging in depths beyond 10 μm.

In a similar approach to utilize multiphoton microscopy for cell imaging, Boksebeld et al.^[^ ^219^ ^]^ used surface‐modified SiC NPs to specifically label and image cancer cells. After aminating the surface of SiC NPs with 3‐aminopropyltriethoxysilane (APTES), the synthetic molecule of poly(‐ethylene glycol)‐folate (PEG‐folate) was conjugated to the aminated surface of NPs to functionalize them for interacting with human hepatocarcinoma‐derived Huh‐7 cells, used here as cancer cells. The PEG molecule was utilized as a spacer to enhance the interaction efficiency between the functionalized NPs and cancer cells. After incubating cells with functionalized NPs and fixing the cells, samples were colored with Nile Red enabling multiphoton microscopy by exciting at 790 nm and capturing the fluorescent signal from Nile Red at 607 nm and the second‐harmonic generation signal from SiC NPs at 395 nm, authors were able to distinguish HuH7 cancer cells from other cell types with high efficiency. No cytotoxic effects from SiC NPs on the proliferation of healthy cells were observed.

**Figure 19:**
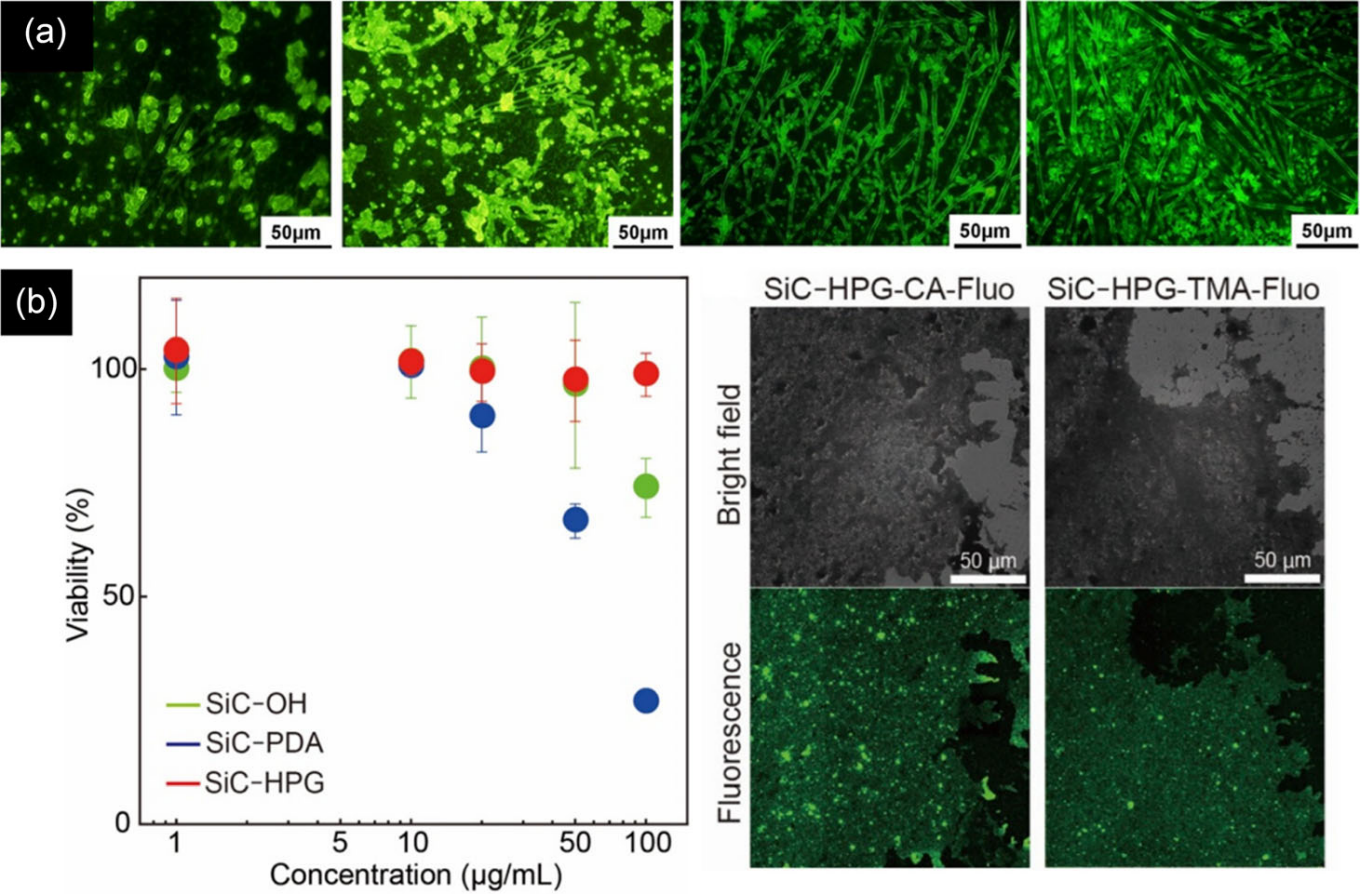
a) Fluorescence images of Aureobasidium pullulans cells after incubation with 3C‐SiC QDs for 3–20 days. The excitation wavelength was 340 nm. Reproduced with permission.^[^ ^95^ ^]^ Copyright 201, Scientific Research. b) Cytotoxicity test and cell imaging results using a 488 nm laser. Reproduced with permission.^[^ ^41^ ^]^ Copyright 2023, American Chemical Society.

As another example of demonstrating the effect of SiC NP surface functional groups on their interaction with live cells, Song et al.^[^ ^95^ ^]^ studied the interaction of electrochemically etched 2.5 nm SiC QDs with cells over a 20‐day period of time. These QDs benefit from a maximal photoluminescence intensity under excitation wavelengths of 320–360 nm as well as a high Stokes shift of 110 nm, almost three times higher than that of fluorescein isothiocyanate, as one of the most widely used fluorescent dyes. In addition, the QCE of QDs enables multi‐color fluorescence from a mixture of QDs with different sizes using one single excitation wavelength. In order to study the interaction between SiC QDs and live cells, as well as the live cell imaging capabilities of these NPs, Aureobasidium pullulans cells were incubated with SiC QDs for several days. As shown in **Figure** **19**a, at the early stages of incubation (3 days), the QDs were observed to accumulate on the surface of cells. This indicated that the QDs are bound to the *Aureobasidium pullulans* cells’ outer membrane via the coupling of organophilic functional groups on the surface of QDs (i.e., carboxyl, hydroxyl, and oxy groups) with the cell membrane proteins and binding sites. As the incubation time extended to 5 days, the fluorescence signal from QDs was observed to come from the interior of the cell membrane, including nuclei, indicating the uptake of these NPs by cells via endocytosis. The cytotoxicity assessment of the QDs was also carried out by extending the incubation period further to 20 days, which did not result in any change in the form and growth of live cells, demonstrating the absence of any cytotoxicity.

In another survey, different coatings such as polydopamine (PDA) and hyperbranched polyglycerol (HPG) were used to extend the biomedical applications of SiC NPs by improving their surface chemistry characteristics.^[^ ^41^ ^]^ To achieve this, first, dispersed SiC NPs were made by de‐aggregating hexagonal 3C SiC NPs through a thermal‐oxidation chemical‐etching method. Then, these NPs were incubated with PDA and HPG suspensions to form different coating layers of NPs. Characterizing the resultant 120 nm NPs revealed that using different coatings not only affects the biochemical properties of these NPs but also affects their optical properties. Although both OH‐SiC and HPG‐SiC had OH functional groups on their surface, SiC‐HPG demonstrated an almost stable absorption at 400 nm in PBS, while both OH‐SiC and PDA‐SiC showed a significant decline in absorption intensity within an hour, indicating precipitation of the NPs. The HPG‐coated SiC NPs also demonstrated high stability over a wide range of pHs, from 1 to 13. The HPG coating provided superior hydrophilicity and colloidal stability as well as reduced nonspecific biomolecule absorption while also providing OH terminations on the outermost layer of the coating, enabling potential for further modification of the surface with another functional group, such as a carboxyl, amine, alkyne, or azide. The cytotoxicity tests of the CCK‐8 assay were carried out using HeLa cells with an incubation time of 22–26 h at 37 °C and subsequent absorbance measurement at 450 nm. The results, as shown in Figure 19b, showed that using HPG coating significantly reduces the toxicity of SiC NPs, even at concentrations as high as 100 μg mL^−1^, which might be required in imaging applications. To label cells and assess the bioimaging potential of these SiC NPs, fixed HeLa cells were first incubated with a biotinylated anti‐CD‐44 antibody suspension. After adding streptavidin to the suspension, the SiC−HPG−Bio−Fl complex was added to the reaction mixture to conjugate the fluorescence‐labeled SiC‐HPG to the cells. Finally, SiC‐PDA‐GNP complexes were synthesized taking advantage of PDA's ability to reduce metal ions on its surface, which resulted in very uniform SiC‐PDA‐GNP structures compared with direct conjugation of SiC and GNPs. This SiC‐PDA‐GNP complex can be used in photothermal applications as nano heaters with controllable heat by changing the excitation laser power.

In another attempt to study the role of SiC QD surface functional groups on the behavior and performance of SiC QDs in cell imaging and biosensing applications, Cao et al.^[^ ^158^ ^]^ used polyethylene glycol (PEG) for surface modification of QDs in order to improve their stability and biocompatibility. In the first report of the synthesis and biomedical application of 2D SiC QDs, two‐dimensional ultrathin QDs were utilized for cell imaging and as an intracellular transporter of hybridization chain reaction (HCR) probes for low‐abundance micro‐RNA detection. After synthesizing these monolayered QDs by hydrothermal stacking of SiC powder as uniform sheets, TEM, HRTEM, and fast Fourier transform (FFT) were used for characterizing the SiC nanosheets as well as the resultant 3 nm SiC QD NPs. AFM results indicated a single‐layered atomic crystalline structure by measuring the monolayer thickness to be around 0.5–0.8 nm. The superior optical properties of these 2D SiC QDs were also studied and shown to have a high quantum yield of 7.95%, as well as a long fluorescence lifetime of 2.59 μs with no significant change in their fluorescence intensity after 30 days, representing high fluorescence stability. After modifying the QDs with PEG, UV–vis spectrometry and FTIR were used to verify the successful conjugation of PEG on the surface of SiC QDs. Observing no significant change in the diameter of QDs in different biological media after PEG modification indicated the high suspension stability of SiC‐PEG QDs, which is crucial in any biological application. Additionally, the cytotoxicity of these QDs was studied by MTT viability assays, which showed no significant cytotoxic effects on live cells. In order to study the potential of these QDs for bioimaging applications, three types of cells were used. After 4 h of incubating QDs with A549, HeLa, and normal human dermal fibroblasts (NHDF) cells, all groups showed intense blue fluorescence from the membrane and cytoplasm of cells in 450 nm excitation, indicating successful uptake of QDs by cells. The absence of a fluorescence signal from the nuclear region verified that these QDs can easily enter the cell membrane without passing through the nuclei membrane and causing genetic disruption. Taking advantage of this special characteristic of SiC‐PEG QDs, authors have used these NPs as intracellular transport agents for transferring two hairpin DNA probes, as HCR probes, into the target cells for detecting miRNA‐21, one of the most frequently upregulated genes in cancerous cells. Since the entrance of DNA probes into the nuclei region can cause changes in the genetic information of target cells, the capability of SiC QDs for easily entering the cell membrane while not entering the nuclei is vital for this biosensing application. In the miRNA‐21 detection process, first, the two stabilized hairpin probes for detecting miRNA‐21 by HCR were loaded on SiC‐PEG monolayers via van der Waals forces. After confirming the formation of a long double‐stranded DNA in the presence of miRNA‐21 using Gel Electrophoresis, a successful amplification process was further verified by comparing the fluorescence signal from cells incubated with SiC‐PEG QDs modified with only one and with both HCR hairpins. The abundance of miRNA‐21 in both cancerous A549 and HeLa cells resulted in intense fluorescence signals from these two cell lines after incubation with two HCR probes. Furthermore, the absence of a fluorescence signal in the case of non‐cancerous NHDF cells indicated effective selectivity of this simultaneous cell imaging and miRNA detection design.

#### 6.1.2 Biosensors

Si‐based QDs can contribute to improving photoluminescent systems, such as optical biosensors, by providing a stronger response in comparison with traditional dye molecules or fluorescent proteins.^[^ ^220^ ^]^ In contrast with,^[^ ^158^ ^]^ which only used the good cellular uptake of SiC QDs, and not their superior optical properties, other researchers have utilized QDs in biosensing systems as signaling units. For instance, Li et al.^[^ ^159^ ^]^ used fluorescence quenching of SiC@BSA NPs for the detection of *S. salivarius* for saliva identification in forensic applications. The 14 nm SiC@BSA NPs were formed by adding BSA to the suspension of hydrothermally synthesized SiC QDs, which resulted in the enhancement of both intensity (5‐fold) and stability (<15% over 12 weeks) of the fluorescence signal from NPs in comparison with the original SiC QDs. After characterization of these NPs using TEM, UV–vis, and fluorescence spectroscopy, GH12 peptides with a sequence of GLLWHLLHHLLH‐NH2 (98%) that can specifically bind to *S. salivarius* were conjugated to the SiC@BSA NPs by N‐hydroxysuccinimide/1‐ethyl‐3‐(3‐dimethylaminopropyl)carbodiimide (NHS/EDC) chemistry. The principle of this detection system relies on the quenching of the fluorescence signal from SiC@BSA NPs upon binding to the target when excited at 320 nm. Detection performance assessment tests were carried out using different concentrations of *S. salivarius* ranging from 10^2^ to 10^7^ cells mL^−1^, and an incubation time of 25 min at 37 °C. Additionally, the selectivity of this biosensing design was evaluated using samples spiked with several non‐target bacteria, including *E. coli* and *S. aureus*. Besides achieving a low limit of detection (LOD) of 25 cfu mL^−1^ in a linear range of 10^2^–10^7^ cells mL^−1^ in only 40 min of detection time, SiC@BSA NPs modified with GH12 peptides were shown to have excellent antibacterial efficacy after culturing samples using the Agar plate assay. Similarly, Mognetti et al.^[^ ^221^ ^]^ used 3CSiC QDs to specifically kill cancer cells. The 2 nm SiC QDs synthesized through an electrochemical etching process were incubated with two cancerous cell lines, as well as one immortalized oral epithelial cell line, as the control group to see the effect of SiC QD treatment on cell morphology and viability among different cell types. In both scenarios of incubating QDs with control and cancerous cells for 18 and 72 h, the cytotoxic effect assessed through the MTT assay was shown to be significantly higher for the two cancerous cell lines compared with the control group of non‐cancerous cells.

In a similar design, Yao et al.^[^ ^160^ ^]^ used SiC QDs synthesized through a one‐pot hydrothermal method for the detection of *P. mirabilis*, a potentially fatal foodborne pathogen, in a biosensing system design based on the fluorescence quenching of SiC QDs upon binding to target cells. After characterizing the 14 nm SiC QDs using TEM, amino‐functionalized aptamers were conjugated to the carboxyl groups on the surface of QDs via NHS‐EDC chemistry. The successful conjugation was confirmed by observing the hyperchromic effect‐induced blue shift in the absorbance spectrum of SiC‐aptamer in comparison with SiC QDs in UV–vis spectroscopy results. The fluorescence stability of the SiC‐aptamer complexes was also assessed by measuring the fluorescence intensity of QDs over 8 weeks, which resulted in an intensity decline of only 6.43%, indicating enduring stability of SiC QDs. After optimizing the detection time to 35 min, the detection performance was studied in pure milk samples spiked with 10^3^–10^9^ cells mL^−1^ *P. mirabilis*. Achieving a LOD of 526 cfu mL^−1^ with detection accuracies higher than 87% when used with real forensic sample media such as milk powder, blood, and urine indicated the potential for using such aptameric biosensors in forensic applications of food poisoning incidents.

In another example of SiC QD applications in biosensing systems, Sun et al.^[^ ^161^ ^]^ used SiC QDs synthesized through a one‐step hydrothermal method for the detection of ambient particulate matter. In contrast with the previously mentioned designs that used peptide sequences^[^ ^159^ ^]^ and aptamers^[^ ^160^ ^]^ for functionalizing SiC QDs for detection purposes, the detection principle of this study relies on the injection of holes by hydroxyl radicals on the surface of ambient particulate matter onto SiC QDs. This takes advantage of the fact that hydroxyl radicals have the highest standard redox potential among all reactive oxygen species.^[^ ^171^ ^]^

#### 6.1.3 Ion Detection

In another similar design, Chen et al.^[^ ^171^ ^]^ used the reaction between Fe^3+^ ions and the oxygen‐containing groups of 4H‐SiC QDs for the highly selective detection of Fe^3+^ ions. Considering the vital role of Fe^3+^ ions in numerous biological processes such as oxygen uptake and metabolism by cells, monitoring abnormal levels of Fe^3+^ ions in biological fluids can be of great importance. As with the abovementioned reaction results in the fluorescence quench of 4H‐SiC QDs, the fluorescence signal from QDs was shown to linearly decrease when the concentration of ions changed from 1 to 20 μM in the detection samples. Considering the vital role of Fe^3+^ ions in numerous biological processes such as oxygen uptake and metabolism by cells, monitoring abnormal levels of Fe^3+^ ions in biological fluids can be of great importance. Similarly, Yang et al.^[^ ^126^ ^]^ used aggregation‐induced fluorescence quenching of 20 nm SiC NPs in the presence of Au^3+^ ions in order to address the environmental pollution and biological toxicity issues related to industrial usage of gold in catalysis, electrochemistry, and medicine. Total elimination of a fluorescence signal from SiC NPs, as well as the formation of a yellowish precipitate upon incubation with Au^3+^ ions, indicated the successful detection with a LOD of 10^−5^ m and a high specificity among 16 other ions.

### 6.2 Optoelectronics

#### 6.2.1 Light Emitting Diodes

**Figure 20:**
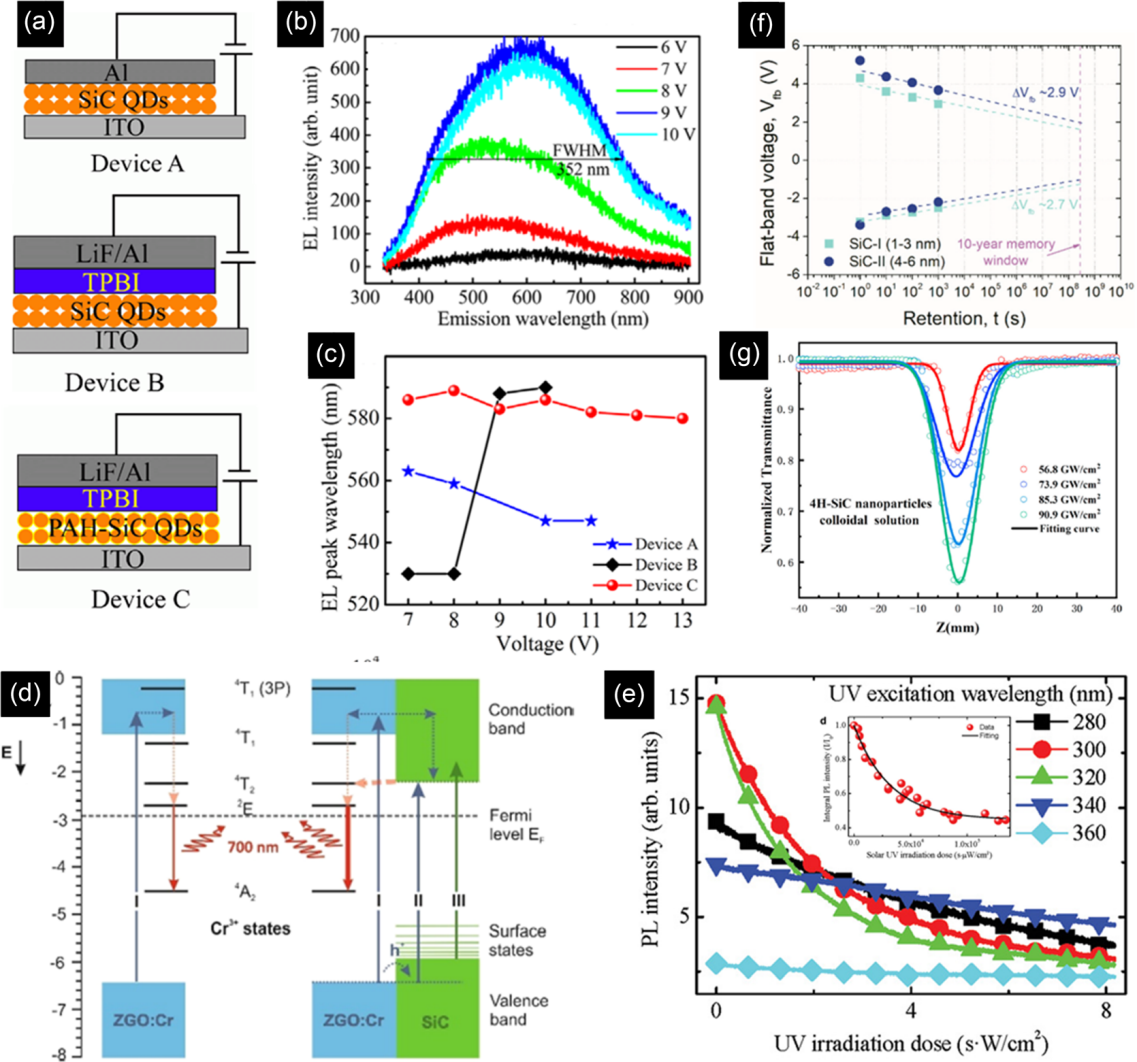
a) Electroluminescence WLEDs with three A, B, and C configurations. b) The EL spectrum of device C. c) Performance of EL devices at different voltages. Reproduced with permission.^[^ ^107^ ^]^ Copyright 2018, John Wiley and Sons. d) Proposed band diagram and a series of excitation pathways that are enabled by the core–shell structure. Reproduced with permission.^[^ ^254^ ^]^ Copyright 2021, American Chemical Society. e) 392 nm emission peak intensity of 3C‐SiC QDs under various excitation wavelengths as a function of UV irradiation dose (Inset shows the normalized integrated PL intensity as a function of accumulated solar UV irradiation dose). Reproduced with permission.^[^ ^211^ ^]^ Copyright 2019, John Wiley and Sons. f) Memory as measured by flatband voltage retention. Reproduced with permission.^[^ ^225^ ^]^ Copyright 2020, MDPI. g) Z‐scan results indicated a sizeable nonlinear effect causing a dip in the transmitted intensity. Reproduced with permission.^[^ ^42^ ^]^ Copyright 2022, Elsevier.

The use of green and red QDs in the backlights of wide color‐gamut liquid crystal displays is now widespread. Blue‐emitting nitride‐based LEDs illuminate the QDs.^[^ ^222^ ^]^ However, preparing an electrically driven white‐emitting LED (WLED) based on single phosphor QDs, which are capable of covering the entire visible (RGB) spectrum, is a challenging issue. Recently, lead‐free perovskites have been introduced to be used for WLEDs due to their wide PL FWHM, related to self‐trapped excitons.^[^ ^223^ ^]^ Surprisingly, SiC QDs could also be used as a novel candidate for electrically driven WLEDs. By taking advantage of design engineering, three electroluminescent white LEDs based on SiC QDs (*D* = 3.1 nm with size distribution of 1.9–4.5 nm) with three configurations (Device A, B, and C in **Figure** **20**a) were fabricated. The EL spectrum of the best device (C) in Figure 20b shows a wide emission FWHM (340–360 nm), covering the visible region (350–900 nm). While the shorter emission wavelengths stem from surface state recombination, the longer wavelengths are associated with the recombination of separately injected holes and electrons into energy levels within the QDs. According to Figure 20c, although Device A suffers from low EL performance at high voltages due to the direct contact of anode and cathode with QDs and increasing non‐radiative recombination, introducing TPBI with high electron mobility between QDs and LIF/Al enhances device performance. However, unbalanced charge injection weakened the EL results of Device B at low voltages. This problem was addressed by embedding QDs in PAH as an HTL which decreases not only the probability of non‐radiative recombination but also mitigates imbalanced charge injection due to indirect contact with electrodes and an improvement in hole injection. In this model, the carrier transport mechanism is dominated by quantum tunneling at a low bias (<5.6 V) and direct injection at a high bias (>7.5 V). However, the thickness of TPBI should be optimized since slight changes in thickness from 12 to 16 nm increased the turn‐on voltage from 6 to 8 V, decreasing the electroluminescence intensity significantly.^[^ ^107^ ^]^

#### 6.2.2 Solar Cells

The purpose of the 2022 annual report was to explore the potential suitability of terminated SiC QDs for various roles in dye‐sensitized solar cells. The first principle DFT‐based simulation work studies surface‐hydroxylated and ‐carboxylated SiC QDs for the photoanode, the counter electrode, and the sensitizer. By using perturbation theory and the GW approximation, it is predicted that the complete functionalization of the surface atoms leads to a decrease in the HOMO‐LUMO gap, while for a 50% surface functionalization, the variation of the HOMO‐LUMO gap depends on the position of molecular substituents. This finding is attributed to the hybridization of surface molecular orbitals.^[^ ^224^ ^]^

#### 6.2.3 Heterojunctions

A study of enhanced X‐ray and UV‐excited NIR emission of core–shell nanoparticles was undertaken. After the synthesis of a 1–3 nm SiC core, this core is shown to facilitate the nucleation and growth of SiC core/ZnGa2O4:Cr shell NCs by a hydrothermal method.^[^ ^107^ ^]^ Cr^3+^ is a substitutional impurity on the Ga^3+^ sites in the shell. A Type I heterojunction between core and shell is supported by PLE and UPS measurements. Figure 20d shows the proposed band diagram and a series of excitation pathways that are enabled by the core–shell structure. 700 nm emission from the excited ^2^E level to the ground ^4^A_2_ level of Cr^3+^ may be directly excited within the shell (Pathway I). Excitations that produce electrons and holes in SiC bands (Pathways II and III) must also be considered. A close match between the ^4^A_2_ level and the SiC CB is noted as a possible explanation for the enhancement of the PL processes.

#### 6.2.4 UV Light Detection

The photobleaching of 3C‐SiC QDs was found to be effective in detecting the dose of the UV part of solar light. Regarding the sensitivity of the 392 nm emission band, corresponding to COOH‐related surface states of SiC QDs, to UV light, the PL emission intensity was monitored under various UV irradiation wavelengths (280–360 nm, dose = 8.18 s μW cm^−2^).^[^ ^211^ ^]^ Based on Figure 20e, the reduction of emission intensity was highly dependent on excitation wavelength since the curves corresponding to the 280–300 nm interval rapidly dropped while the trend was slower in the range of 320–360 nm. No photobleaching effect was observed at ≈360 nm as the excitation source loses its power to form photogenerated carriers, indicating the specific relevance to 280–360 excitation wavelengths. Figure 20e inset also shows the potential of the photobleaching property of the SiC QDs in sensing solar UV irradiation with high accuracy (fitting accuracy = 0.97).

#### 6.2.5 Memory Devices

The potential for 3C SiC NCs in MIM charge‐storage‐type memory applications was also proposed and studied. The concept is to enable very low‐temperature deposition of a charge storage material on the gate stack using the nanocrystals applied to CMOS devices. 3C SiC powder is synthesized by a bottom‐up process and is then etched and subsequently fragmented by ultrasonication, yielding 2–5 nm SiC NCs.^[^ ^225^ ^]^ The colloidal NCs are then incorporated into HFO_2_‐based MIM or MIS with different structures. By programming potential differences across the electrodes and then measuring capacitance over a frequency range between 0.01 and 1 MHz, conducting filaments are stated to cause charge transfer and subsequent charge storage on the NCs. This alters band bending, and the charge storage may be captured by plotting the flat band voltage versus the initial bipolar program/erase voltage. Retention is extrapolated to exist over 10 years (Figure 20f).

#### 6.2.6 Nonlinear Optics

In order to study nonlinear optical characteristics, a colloidal suspension comprising 2.8 nm NCs, formed by femtosecond laser pulses, and the well‐known *z‐scan* method was employed.^[^ ^42^ ^]^ Probe laser light was focused and directed through the prepared colloidal nanoparticle suspension. By moving the sample along the z‐axis, changes in the sample position relative to the probe laser focal point took place, and transmitted intensity was observed, indicating a nonlinear optical effect. Sample refractive index is a constant in the absence of the nonlinear effect, and therefore, the changes in refractive index inferred from the observed intensity drop are fundamentally and directly a result of nonlinear optical behavior (Figure 20g). Samples measured under the highest probe laser fluence exhibited the highest nonlinear optical effects as expected.

#### 6.2.7 Quantum Sensing

The realization of Si‐vacancy and divacancy in SiC NPs paves the way for the development of SiC‐qubit‐based sensor devices. As discussed in the chapter “Beyond Quantum Confinement,” all cited examples involve quantum bits within SiC NPs. It is crucial to distinguish between the detection of defect‐related photoluminescence and the realization of addressable spins. For instance, while infrared emission from Si‐vacancy has been observed in particles as small as 10 nm, zero‐phonon lines at low temperatures, along with optically detected magnetic resonance (ODMR) signals, have only been detected in particles sized 300 nm or larger. A single study reported the ODMR signal from divacancy spins in 5‐nm diameter SiC NCs, although the results of the paper were later criticized.^[^ ^197^ ^]^ Indeed, further studies are needed to explore single‐digit SiC NCs with divacancy spins to elucidate the existence and properties of divacancy qubits in SiC NCs. To this end, noninvasive methods for creating paramagnetic defects in SiC are a promising route to generate high‐quality SiC embedding qubits.^[^ ^226^ ^]^ The ODMR signal appears because of optical spin polarization and spin‐selective fluorescence intensity (optical spin readout), which turns these paramagnetic color centers into qubits. The surface passivation techniques demonstrated to successfully eliminate surface defects.

### 6.3 Energy Conversion

#### 6.3.1 Photocatalytic Activities

It has been inferred that the size and shape of a semiconductor particle play a major role concerning photocatalysis. In bulk semiconductor photocatalysts, either holes or electrons are available for reaction due to band bending. However, in colloidal semiconductor particle suspensions, electrons and holes may simultaneously be present on the surface, allowing several reaction routes. When the spatial dimensions of the material are comparable to the de Broglie wavelength of carriers, quantum confinement leads to a transition from continuous to discrete energy levels that could significantly affect photocatalytic activity. The gap between occupied and unoccupied energy levels increases, yielding larger redox potentials that, together with an unchanged solvent reorganization‐free energy, are expected to increase photocatalytic activity. Colloids are often described as uniformly dispersed particles in a continuous phase, neglecting particle–particle interactions. By studying semiconductor NPs in the quantum confinement regime where size has a huge impact on the photocatalytic properties, it was found that surface moieties regulate the interaction between NPs in suspension that result in size‐distribution‐dependent photocatalytic activity. SiC was used as a model material.^[^ ^227^ ^]^ Particles with a diameter just above twice the exciton Bohr radius have significant photocatalytic efficiency comparable to ZnS, but SiC NPs with a diameter below showed negligible activity. However, such molecular‐sized SiC NPs can enhance the efficiency of the larger particles, demonstrating the importance of the size distribution. These findings, together with the mentioned size‐dependent photocatalytic activity, prove that the photocatalytic efficiency of SiC is extremely size‐dependent. To find out more, NPs with diameters below 2aB (1–4 nm, SiC‐I) were prepared and separated from larger particles by centrifugation. The retentate contained the particles larger than 4 nm (4–16 nm, SiC‐II).^[^ ^227^ ^]^

With the development of NCs and QDs, the small size of photocatalysts has received strong interest due to the outstanding physical properties, such as the increased number of surface sites and enhanced charge recombination rates.^[^ ^228^ ^]^ However, the measured properties of QDs photocatalysts are widely influenced by specific aspects of material properties, including NC size, surface chemistry, bandgap, and carrier mobility.

SiC QDs have strong potential to be applied as common photocatalysts and are verified to activate the photocatalytic process and improve dynamics.^[^ ^229^ ^]^ By reviewing the application of SiC QDs with different sizes, it is found that particle size directly relates to the material properties and eventually determines the applications of SiC QDs. To further explain this, the effect of surface moieties on the surface‐mediated energy transfer of SiC QDs is studied and connected with photocatalytic behavior.^[^ ^227^ ^]^ On one hand, the SiC QDs exhibit photocatalytic activity only for 4–10 nm, which is explained by the energy transfer between different QDs with different sizes and corresponding bandgaps. On the other hand, surface chemistry can affect the interaction between QDs. As an example, it is shown that the carboxylic‐terminated surface provides higher photocatalytic activity than the hydroxyl‐terminated surface. For heterogeneous photocatalysts, SiC QDs can be composited with common materials to sensitize catalytic behavior. For instance, SiC QDs are applied with inverse opal (IO) TiO_2_ to achieve better performance for water purification.^[^ ^230^ ^]^ By comparing photocatalysts with and without SiC QDs, it was verified that SiC QDs can improve photocatalytic activity. In the heterojunction of SiC QDs and IO TiO_2_, electrons can be excited easily from the valence band of SiC QDs, and the charges can then be transferred to TiO_2_, where the potential is appropriate for chemical reactions. In conclusion, the key effect of SiC QDs in heterogeneous photocatalysts is to create effective charge separation, which can increase the concentration of photoinduced carriers and can eventually achieve a higher activity of photocatalysts.

#### 6.3.2 CO_2_ Reduction

**Figure 21:**
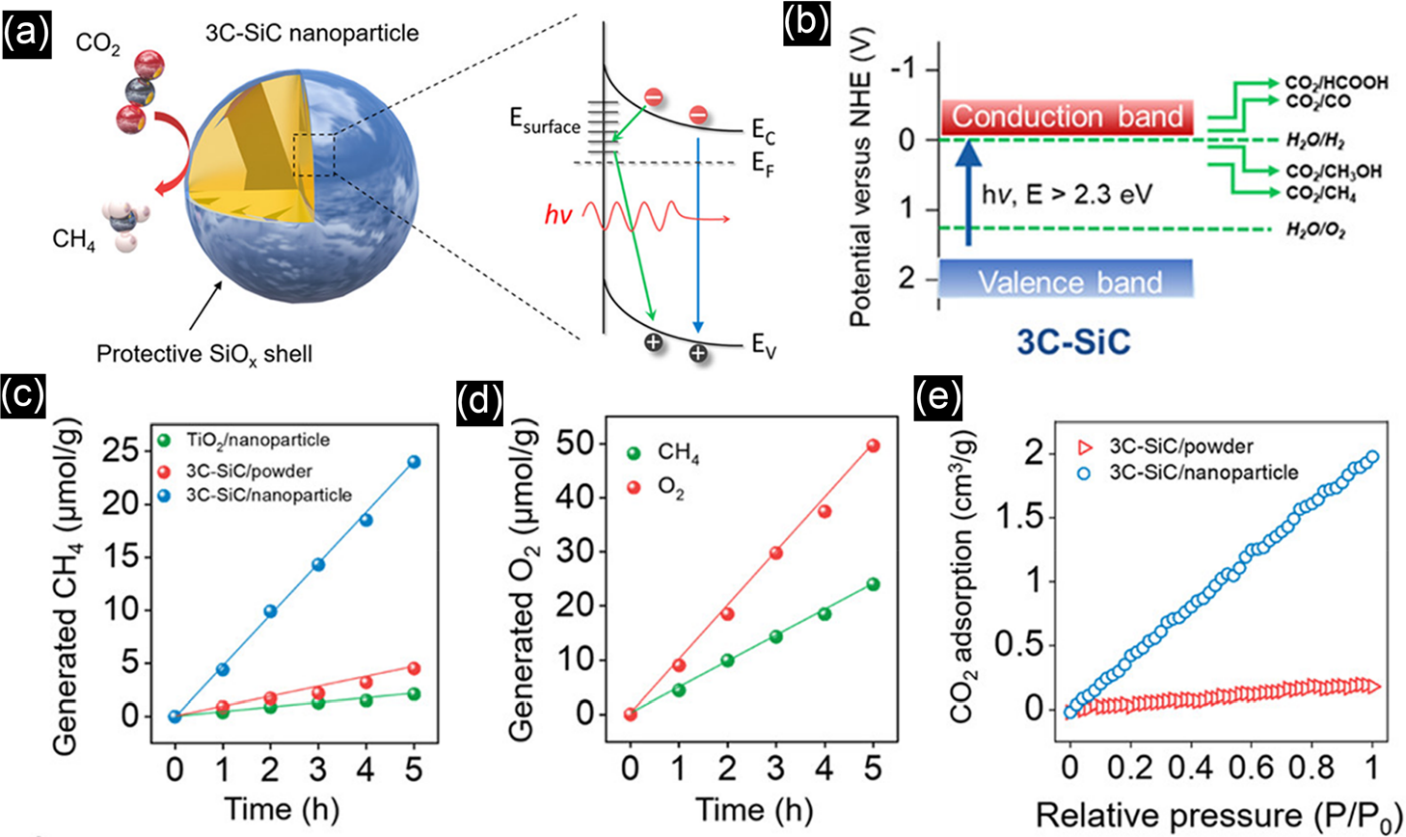
a) Illustration of SiC@SiO_*x*_ photocatalyst for CO_2_ reduction. b) Chemical potential of CO_2_ reduction and H_2_O oxidation with the required band structure. Evolution of c) CH_4_ and d) O_2_ with reaction time compared with other photocatalytic materials. e) Comparison of the CO_2_ adsorption rate of SiC NPs and powders. Reproduced with permission.^[^ ^232^ ^]^ Copyright 2021, American Chemical Society.

Benefiting from the appropriate bandgap of 2.36 eV, 3C‐SiC has the potential to be applied in CO_2_ reduction as a photocatalyst. According to the reduction potential of CO_2_, the reaction can be catalyzed only by the catalysts with a proper energy band structure to reach thermodynamic requirements, where the satisfied semiconductor bandgap should be 1.23–3.00 eV.^[^ ^231^ ^]^ In this case, NPs of 3C‐SiC are designed as the photocatalyst for CO_2_ reduction with high selectivity and efficiency.^[^ ^232^ ^]^ For platform construction, silicon oxide SiO_*x*_ is formed on the surface of SiC NPs, which can not only trap photoinduced carriers but also prevent the core SiC from photo‐corrosion, enabling a longer lifetime as demonstrated in **Figure** **21**a. The catalyzing CO_2_ gas reacts with H_2_O liquid, where CO_2_ is reduced into CH_4_ while water is oxidized into O_2_ according to the chemical potential of each reaction shown in Figure 21b. According to the characterization of reactant formation, the photocatalytic effect is verified to be promoted via SiC@SiO_*x*_ materials. The adsorption rate of CO_2_ is observed to be larger, and the formation of O_2_ and CH_4_ can be measured as in Figure 21c–e.

**Table 4:** Applications based on SiC QDs.

Application	Platform	Device characteristics	Mechanisms	Reference
Cell imaging & Biosensing	2D SiC QDs‐PEG (3 nm)	Strong bright blue fluorescence in the cell membrane and cytoplasm after QD incubation Low cytotoxicity of SiC‐PEG QDs assessed by MTT assay Detection of miRNA‐21	Blue‐fluorescent QDs enter cell membrane by an endocytosis process and used as HCR reagent transporter for low‐level miRNA‐21 detection	[158]
Biosensing	SiC@BSA NPs (14 nm) conjugated to peptides	Target: *S. salivarius* Linear range: 10^2^–10^7^ cfu mL^−1^ LOD: 25 cfu mL^−1^ Detection time: 40 min Detection accuracy >88% QD signal decline less than 15% after 12 weeks	Fluorescence quenching upon binding of target cells to peptide chains on SiC@BSA NPs	[159]
Biosensing	SiC QDs (14 nm) conjugated to aptamers	Target: *P. mirabilis* Linear range: 10^3^–10^9^ cfu mL^−1^ LOD: 526 cfu mL^−1^ Detection time: 35 min Detection accuracy > 87% QD signal decline of 6.43% after 8 weeks	Fluorescence quenching upon binding of target cells to aptamers on SiC@BSA NPs	[160]
Live cell labeling and imaging	SiC QDs (2.5 nm)	Strong green fluorescence on the cell membrane after QD incubation No cytotoxic effects after 20 days in incubation	QD uptake into cell membrane	[95]
Immunomodulatory agent	SiC QDs (1.5 nm) SiC‐OH QDs (1.7 nm) SiC‐NH_2_ QDs (1.8 nm)	QDs affect cell morphology and differentiation of monocytes No significant cytotoxic effects from SiC‐OH and SiC‐NH_2_ QDs assessed by MTS assay	The interaction of QDs with cells is governed by QD surface functional groups and biomolecular corona of colloidal NPs	[256]
Bioimaging	SiC‐PDA and SiC‐HPG QDs (120 nm)	Strong green fluorescent signal from cell membrane after QD incubation Lower cytotoxic effects from SiC QDs with HPG modification Superior hydrophilicity and colloidal stability after HPG modification Reduced nonspecific biomolecule absorption after HPG modification	Using streptavidin‐biotin for conjugating SiC‐HPG QDs to cells modified with anti‐CD‐44 Using PDA's ability of reducing metal ions on its surface to make uniform SiC‐PDA‐GNP structures	[41]
WLED	SiC QDs (3.1 nm)	Improved charge injection balance by embedding QDs in HTL material PAH and inserting TPBI between QDs and LIF/Al	TPBI with high electron mobility between QDs and LIF/Al enhances device performance.	[107]
UV light detection	SiC NCs	Sensing the solar UV irradiation by photobleaching property of 392 nm emission band, corresponds to COOH‐related surface sates of SiC QDs	Emission most sensitive to prior excitation in 280–300 nm range.	[211]
Memory Devices	2–5 nm SiC NCs in HfO_2_‐based MIM or MIS structures	Programmed state detected by determining flat band voltage 10 years extrapolated estimate of memory retention	Charge storage	[225]
Quantum sensing	Divacancy defect in SiC NC	Detects magnetic field using magnetic resonance 1180 nm fluorescence dips in intensity due to magnetic field.	Optically detected magnetic resonance spectroscopy	[226]
Photocatalysts	SiC QDs@IO TiO_2_ composites	Improve photocatalytic activity Apparent kinetic constant: larger Radicals participate degradation: higher	Photogenerated charge carriers transfer from SiC QDs to another catalyst	[230]
Photocatalysts	SiC@SiO_*x*_ core–shell NPs	CO_2_ selectivity: 94% CH_4_ generation rate:4.9 μmol g^−1^ h^−1^ Average life time: 25.02 ns High selectivity	CO_2_ adsorption and then reduction by photogenerated charge carriers	[232]

## 7 Conclusion and Outlook

After presenting a general overview of strategies to improve radiative recombination in indirect bandgap SiC, we comprehensively focused on the optical properties of SiC QDs. There is a strong consensus among researchers that the excitation‐independent PL band of SiC QDs corresponds to surface states of particles smaller than 3 nm. Although some researchers have attributed the excitation‐dependent PL band of SiC NCs to QCE in larger particles (3–5.8 nm), others have shown that the PL peak redshift with reducing excitation energy is not related to QCE or size‐dependent emission. Instead, they provided strong evidence that this behavior originates from other sources, such as defects (e.g., stacking faults), and abundant surface‐related emission states. These fluorescent sources may form a near‐continuum of states inside the confined energy gap, which are activated by different excitation energies. Therefore, it can be concluded that the radiative recombination from SiC QDs may come from only surface and or defects states.

Researchers have widely studied the effect of various oxygen‐containing surface functional groups and organic molecule/shell passivation of SiC QDs on the PL peak wavelength and PLQYs. Despite attempts, the resulting PLQY has generally remained lower than 18% for several years. Recently, synthesis of 2D SiC QDs with significantly enhanced PLQY as high as 60% using plasma annealing modification technique in the oxygen‐shielding environment which removes excess carbon and forms a balanced Si:C stoichiometry on the outer surfaces is claimed.

It is now more evident that oxygen‐containing surface groups create emission states within the bandgap, which cause a redshift in the PL peak and reduce the PLQY. Additionally, a carbon‐rich surface weakens and broadens the emission, while a silicon‐rich surface reduces the activity of key emitting surface groups. To improve the PLQY of spherical SiC QDs, it is suggested to (i) perform synthesis in an oxygen‐shielded environment, (ii) use precursors that limit excess surface carbon, and (iii) apply post‐treatments that establish a balanced Si:C surface stoichiometry with favorable terminations such as SiCH_3_ and SiOC_*x*_H_*y*_, confirmed by first‐principles modeling.

Since controlling the surface of SiC QDs can be challenging, doping or co‐doping strategies are highly recommended for further investigation. For example, nitrogen doping has shown great capability to intensify the PL peak of SiC QDs, although the PLQY was not reported. This approach was highly efficient in boosting the PLQY of MXene, graphene, and lead‐free perovskite QDs from 1% to 95%. In certain cases, doping has been shown to effectively modify the electronic structure of QDs, potentially converting an indirect bandgap into a direct one.^[^ ^233^ ^]^ To explore and predict such transitions, simulation models such as DFT and hybrid functional approaches (e.g., HSE06) can be employed to analyze the band structures of singly and co‐doped QDs.

The current microLED industry devotes extensive efforts to achieve surface passivation, and it focuses on the mitigation of surface‐assisted nonradiative emission. The current QD industry is likewise dominated by direct gap semiconductors in which surface states are managed by core–shell structures and ligands. As the sizes of semiconductor nanoparticles decrease, surface management becomes increasingly critical. In contrast, SiC surface‐enabled radiative recombination becomes more prevalent, more favorable, and more practical as particle size decreases. Moreover, SiC QDs benefit from various fascinating advantages. They are substantially chemically, structurally, and optically stable compared to Pb‐ and Cd‐based QDs suspended in highly toxic organic solvents. Moreover, these toxic QDs require solid or liquid ligand exchange purification for solid‐state thin film preparation, severely diminishing device performance and increasing production cost, while SiC QDs can be prepared by ligand‐free synthesis methods. More importantly, thanks to recent breakthroughs in enhancing the PLQY of blue‐emissive SiC QDs to over 60%, and with continued progress expected in the coming years, these cost‐effective materials may become strong competitors to commercial GaN in next‐generation electroluminescent LEDs. Moreover, fluorescent, SiC QDs with a sharp emission peak and high PLQY can also be targeted as a next generation of materials for use in LCD display backlights and particularly microLED displays, making them highly attractive for advanced display applications.

UV detectors play a critical role in environmental monitoring by enabling the assessment of air quality, ozone depletion, and UV‐induced ecological effects. SiC QDs with a strong UV absorbance edge and excellent stability can be promising candidates for such applications. By forming a heterojunction with monolayer graphene, flexible and high‐sensitivity planar UV devices can be developed, offering a robust platform for next‐generation UV sensing technologies.

Despite the successful detection of Fe^3+^ ions using SiC QDs, the potential of surface‐functionalized SiC QDs for sensing a broader range of metal ions, including Ag^+^, Cu^2+^, and Cr^3+^, which pose significant health risks when present above critical concentrations, remains largely unexplored. In contrast to the extensive research on carbon‐based, BN, graphene, and MXene QDs for the detection of proteins, organic acids, mRNAs, and various environmental pollutants, there is a noticeable absence of sensors developed specifically using SiC QDs.^[^ ^234^, ^235^, ^236^, ^237^ ^]^ Furthermore, owing to the presence of multiple fluorescent centers with varying emission wavelengths and pH sensitivity, SiC QDs exhibit considerable potential for use in pH sensing applications. This highlights a promising opportunity for their application in advanced sensing technologies.

Thanks to their biocompatibility and ultrasmall size, SiC QDs can pass cell membranes without any cytotoxic effects and can act as signaling agents for cell labeling and bioimaging, immunomodulatory ex vivo stimuli for immune cell therapies, and in biosensing platforms as signaling agents. Although bare QDs can be used in some bioimaging platforms, specific applications in targeted bioimaging, diagnostic, therapeutic, and drug delivery^[^ ^193^, ^194^ ^]^ applications require modification of the surface of QDs with either a shell,^[^ ^238^ ^]^ small molecules with reactive functional groups,^[^ ^239^ ^]^ or biomolecules such as proteins and aptamers.^[^ ^160^ ^]^ By surface functionalizing SiC QDs and loading them with therapeutic agents, these nanoparticles can be engineered to remain stable during circulation and deliver drugs specifically to the tumor microenvironment or even directly to the cell nucleus. The inherent fluorescence of SiC QDs allows real‐time monitoring of drug release, enabling precise control over the therapeutic process. Similar strategies have been demonstrated using carbon‐based QDs, where drug molecules such as doxorubicin were successfully loaded onto the QD surface and released in response to intracellular pH changes or enzymatic triggers.

While SiC QDs have shown promise for in‐vitro biomedical applications such as biosensing and bioimaging, further applications in near‐infrared (NIR‐I, 750–900 nm) imaging for applications like vascular imaging and fluorescence labeling, as well as NIR‐II (1000–1700 nm) deep tissue imaging, remain limited due to several challenges. Although different methods, such as dual photon excitation, have been used to overcome this limitation, thus enabling SiC QDs to be used for deep tissue imaging (above 10 μm), there is still room for further research in this direction. One possibility is utilizing neutron‐irradiated SiC QDs having Si vacancies that can be excited by wavelengths above 800 nm and that emit light about 900 nm. Another approach can be utilizing lanthanide‐doped upconversion nanoparticles in SiC QD systems. In this system, the lanthanide‐doped SiC QDs are excited with NIR light, enabling the conversion of low‐energy NIR photons into higher‐energy visible or ultraviolet emissions.^[^ ^240^ ^]^ This process is particularly advantageous for biological imaging, as NIR excitation within the biological window (700–1000 nm) enables deep tissue penetration while minimizing autofluorescence and photodamage, resulting in high signal‐to‐noise ratios. By combining the intrinsic chemical stability and biocompatibility of SiC with the unique optical tunability of lanthanide ions, this approach could yield a new generation of high‐performance, nontoxic probes for deep‐tissue bioimaging and theranostic applications. However, assessing any potential cytotoxic effects of these lanthanide‐doped SiC QDs is crucial prior to using them in any biological applications. Overall, the results reported in the literature for utilizing lanthanide‐doped nanoparticles in biological applications seem promising^[^ ^241^, ^242^, ^243^, ^244^ ^]^ and can be considered a direction for further work on SiC QDs.

Despite the fact that cellular cytotoxicity assays such as MTT and MTS have been extensively utilized for SiC QDs,^[^ ^245^ ^]^ the assessment of organ toxicity in the case of in vivo studies seems to be overlooked. For example, the liver is always considered the main organ for the accumulation and metabolism of QDs, causing many health issues.^[^ ^246^, ^247^ ^]^ Regarding the possibility of carbon‐based nanocarrier degradation before entering the target cells, the effect of functionalized groups should be considered. In the case of using SiC QDs in detection applications, more sophisticated biosensing designs can be employed to improve the detection sensitivities. For example, various sensitivity‐enhancing strategies such as silica‐based mesoporous nanostructures^[^ ^248^, ^249^, ^250^ ^]^ can be used in combination with SiC QDs in order to lower the LOD of biosensing systems. Moreover, there are still many opportunities in biosensing applications since a broad spectrum of targets exists that can be detected by SiC QDs.

SiC QDs with a high surface‐to‐volume ratio have great capability to be used in energy‐based applications such as photo/electrocatalysis, degradation of pollutants, hydrogen production, and CO_2_ reduction. On one hand, SiC exhibits a strong visible‐light response. On the other hand, SiC also possesses conduction and valence band positions that are well suited for photocatalytic water splitting. Compared with other wide‐bandgap semiconductors, its CB is more negative, giving its photo‐generated electrons stronger reducing power.^[^^]^ However, the wide bandgap also limits the application of SiC regarding the chemical potential of chemical reactions. This can be modified by doping or producing appropriate heterojunctions with other well‐known materials such as Pt and Au.

Given the compelling case for SiC QDs to play a significant future role in a broad range of fields, SiC QDs will undoubtedly continue to enjoy widespread attention and vigorous further development.

## Conflict of Interest

The authors declare no conflict of interest.
